# Molecular switch of the dendrite-to-spine transport of TDP-43/FMRP-bound neuronal mRNAs and its impairment in ASD

**DOI:** 10.1186/s11658-024-00684-5

**Published:** 2025-01-15

**Authors:** Pritha Majumder, Biswanath Chatterjee, Khadiza Akter, Asmar Ahsan, Su Jie Tan, Chi-Chen Huang, Jen-Fei Chu, Che-Kun James Shen

**Affiliations:** 1 PhD Program in Medical Neuroscience, Taipei Medical University, Taipei, Taiwan (R.O.C.); 2 Institute of Molecular Medicine, College of Medicine, National Chen Kung University, Tainan, Taiwan (R.O.C.); 3 Institute of Molecular Biology, Academia Sinica, Nangang, Taipei, 115 Taiwan (R.O.C.)

**Keywords:** TDP-43, pFMRP (S499), mRNP granule, RNA binding protein (RBP), Posttranslational modification, Kinase, Phosphatase, DHPG, Potentiation, Long-term depression (LTD), Translation status, Immunofluorescence staining, Live cell imaging, High-resolution imaging

## Abstract

**Background:**

Regulation of messenger RNA (mRNA) transport and translation in neurons is essential for dendritic plasticity and learning/memory development. The trafficking of mRNAs along the hippocampal neuron dendrites remains translationally silent until they are selectively transported into the spines upon glutamate-induced receptor activation. However, the molecular mechanism(s) behind the spine entry of dendritic mRNAs under metabotropic glutamate receptor (mGluR)-mediated neuroactivation and long-term depression (LTD) as well as the fate of these mRNAs inside the spines are still elusive.

**Method:**

Different molecular and imaging techniques, e.g., immunoprecipitation (IP), RNA-IP, Immunofluorescence (IF)/fluorescence in situ hybridization (FISH), live cell imaging, live cell tracking of RNA using beacon, and mouse model study are used to elucidate a novel mechanism regulating dendritic spine transport of mRNAs in mammalian neurons.

**Results:**

We demonstrate here that brief mGluR1 activation-mediated dephosphorylation of pFMRP (S499) results in the dissociation of FMRP from TDP-43 and handover of TDP-43/*Rac1* mRNA complex from the dendritic transport track on microtubules to myosin V track on the spine actin filaments. *Rac1* mRNA thus enters the spines for translational reactivation and increases the mature spine density. In contrast, during mGluR1-mediated neuronal LTD, FMRP (S499) remains phosphorylated and the TDP-43/*Rac1* mRNA complex, being associated with kinesin 1-FMRP/cortactin/drebrin, enters the spines owing to Ca^2+^-dependent microtubule invasion into spines, but without translational reactivation. In a VPA-ASD mouse model, this regulation become anomalous.

**Conclusions:**

This study, for the first time, highlights the importance of posttranslational modification of RBPs, such as the neurodevelopmental disease-related protein FMRP, as the molecular switch regulating the dendrite-to-spine transport of specific mRNAs under mGluR1-mediated neurotransmissions. The misregulation of this switch could contribute to the pathogenesis of FMRP-related neurodisorders including the autism spectrum disorder (ASD). It also could indicate a molecular connection between ASD and neurodegenerative disease-related protein TDP-43 and opens up a new perspective of research to elucidate TDP-43 proteinopathy among patients with ASD.

**Supplementary Information:**

The online version contains supplementary material available at 10.1186/s11658-024-00684-5.

## Background

Dendritic spines protruding from the dendritic surface are subcompartments reflecting the maturation of neurons. Spine plasticity is regulated by different functional proteins, actin filaments, and synaptic transmissions from the excitatory synapses [1]. During synaptic transmission, neurotransmitters, such as L-glutamate, are released from the axon terminals to activate the metabotropic glutamate receptors (mGluR), α-amino-3-hydroxy-5-methyl-4-isoxazole-propionic acid receptor (AMPAR), and *N*-methyl-d-aspartic acid receptors (NMDAR), which alter the Ca^2+^ influx, induce structural changes in the post-synaptic spine, and elicit the generation of new spine [2, 3]. During short-term potentiation (STP), a synapse reverts to the resting state after a brief, or immediate, synaptic transmission [4]. In contrast, signal transmission of high-frequency pulsing stimulation for a long duration, termed long-term potentiation (LTP), causes persistent synapse strengthening and is important for memory formation [5]. However, for synapses to effectively store important information, old memories need to be selectively eliminated by the decrease of spine number, synapse volume, and synaptic strength through the process of long-term depression (LTD) [6–9]. LTP and LTD together modulate synaptic plasticity/memory formation in the brain, and they are regulated by differential protein expression and complex protein–protein interactions within the dendritic spine regions [10, 11].

Immediate early activation of group 1 mGluR (gp1 mGluRs), including mGluR1 and mGluR5, is known to increase spine translation of several spinogenesis-related mRNAs [12]. In contrast, prolonged activation of mGluR1/5 causes mGluR-mediated LTD through a significant decrease in dendritic translation and active synapse formation [13]. In neurons, mRNAs are transported from the soma to the distal dendrites or axons in the form of membrane-less messenger ribonucleoproteins (mRNPs), via active transport on microtubule tracks using motor proteins, such as kinesin 1 [14, 15]. Recruitment of mRNPs to the microtubule motor proteins is performed either directly by RNA-binding proteins (RBPs) or indirectly by adaptor proteins [16]. In resting neurons, mRNPs are trafficked across the dendrites, while translation of the mRNAs remains at the repressed state until being transported into the nearby spine [17]. Relatedly, the actin motor protein myosin V has been shown to translocate TLS (translocated in liposarcoma)-bound mRNP cargo into the dendritic spine regions [18]. However, the exact molecular mechanistic switch(es) for the dendrite-to-spine transport of mRNAs, instead of transporting across the dendrites, under different synaptic transmissions have remained elusive.

Impairment of mGluR-dependent signaling pathways and spine shrinkage have been documented in both neurodegenerative diseases, e.g., Alzheimer’s disease (AD) [19], and in neurodevelopmental disorders, e.g., autism spectrum disorder (ASD) [20] In particular, ASD is closely associated with the malfunction of mGluR- and NMDAR-mediated LTD and other synaptic transmissions, leading to changes in the spine structure, learning/memory deficiency, social impairment, hyperactivity, etc. [20, 21]. ASD refers to a spectrum of disorders that are heterogeneous in etiology and phenotypes. Although the exact causes of more than 70% of ASD cases are still unknown [22], genetic mutations and/or environmental factors play essential roles in the pathological initiation and progression of the disease [23]. Among the factors closely associated with ASD is the Fmr1 gene product FMRP. This is an RNA-binding protein (RBP), the loss of function of which can cause fragile X syndrome (FXS), as well as ASD [24, 25]. In fact, FXS is the most common monogenic subtype of ASD, accounting for 1–6% of total patients with ASD [26]. FMRP is a translational regulator of a wide range of mRNAs [27] and thus essential for proper synaptic architecture and plasticity of neural dendrites [24] and axon growth cones [28]. Moreover, it also binds and carries a subset of mRNAs, such as *PSD-95* mRNA, into the dendritic spine [29, 30]. Consequently, patients with the subtype of ASD with overlapping FXS, as well as Fmr1 knock-out (KO) mice, with no or a very low level of FMRP in the brain tissues, such as the hippocampus and cortex, exhibited compromised dendritic and axonal arborization, immature spine and synapses, learning/memory difficulty, hyperactivity, and cognitive impairment [31]. However, in patients with another subtype of ASD, the level of FMRP phosphorylated at S499, i.e., pFMRP (S499), is reduced in the cerebellar vermis and superior frontal cortex regions of their brain [32]. Consequently, a subpopulation of FMRP-targeted neuronal mRNAs are dissociated from the FMRP/CYFIP1 translation inhibitory complex, resulting in upregulation of translation of these mRNAs in the brain of those patients [33]. Interestingly, a significant portion of patients with ASD also show neuron loss and other neurodegenerative disease-like pathology [34, 35], the molecular basis of which is still not clear.

The neurodegenerative disease-associated RNA-binding protein TDP-43 is required for regulation of neuronal functions in vivo [36–40]. Mechanistically, it collaborates with FMRP to regulate the transport/translation of a subset of dendritic mRNAs that are important for spinogenesis and learning/memory development, e.g., *Rac1*, *Map1b*, and *GluR1* mRNAs [41, 42]. These RNAs are recruited to FMRP-associated translation and transport complexes by TDP-43 through a physical interaction between these two proteins [43, 44]. Interestingly, similar to the dendrites, both TDP-43 and FMRP play important roles in axonal mRNA transport and translation that regulate the structure and function of presynapses [45, 46]. Notably, a loss of function of TDP-43, including the regulation of axonal [47] and dendritic [43, 48] transport of mRNAs in neurons, would lead to the development of neurodegenerative diseases, including amyotrophic lateral sclerosis (ALS), frontotemporal dementia (FTD), etc. [40, 49–51]. Furthermore, bioinformatics analysis indicates that many mRNAs associated with both TDP-43 and FMRP are involved in ASD-related aberrant spine morphology and increased spine translation [44, 52]. Whether and how the reduction of pFMRP (S499) level contributes to the neuronal dysfunction of patients with ASD remain to be investigated.

Although it has been known that mRNAs trafficking along the neuronal dendrites enter the spine as needed upon synaptic transmissions, the underlying signaling cascades are still unexplored. In this study, we used molecular and imaging approaches to analyze the mechanistic roles of TDP-43 and FMRP in the regulation of dendrite-to-spine transport of TDP-43-bound mRNAs, e.g., *Rac1* mRNA, during brief gp1 mGluR-mediated activation and LTD, respectively. We show that changes in the phosphorylation status of FMRP act as the molecular switches of dendrite-to-spine transport of TDP-43-bound *Rac1* mRNA in the hippocampal neurons. Experiments using a valproic acid (VPA)-treated ASD mouse model further suggest that reduced FMRP phosphorylation, and consequent dissociation of TDP-43 from FMRP, leading to dysregulated dendrite-to-spine transport of TDP-43-bound mRNAs under gp1 mGluR-mediated activation and mGluR-mediated LTD, could be one of the major causes of ASD pathology.

## Methods

### Experimental model and subject details

Fourteen-day pregnant FVB mice were purchased from the National Laboratory of Animal Center (NLAC), Taipei, Taiwan to prepare the primary hippocampal neuron culture. Seven-day pregnant FVB mice were purchased from NLAC for peritoneal injection of VPA to establish the ASD mouse model. HEK293T cells were obtained from ATCC.

### Method details

#### Plasmid construction

pRFP-TDP-43 encoding RFP-tagged mouse TDP-43 (Tardbp, GenBank: NM_145556.4) and pGFP-FMRP (WT) encoding GFP-tagged mouse FMRP (GenBank: NM_008031.3) have been described previously [43]. Using the primers detailed below, point mutations were inserted into the wild-type FMRP construct to generate the dephosphomimetic mutant FMRP(S499A) and phosphomimetic mutant FMRP(S499D) constructs. The constructs were ultimately cloned into pGFP C1 plasmid at EcoRI/SalI sites to generate pGFP-FMRP (S499A) and pGFP-FMRP (S499D). Primers used in plasmid constructions were as follows:

FMRPEcoRI F: 5′-GATCGAATTCATGGAGGAGCTGGTGGTG-3′

FMRPS499A R1: 5′-TCTGTTTCAGCAGCATTTGA-3′

FMRPS499D R: 5′-TTCATCAGCATTTGATGCTTCA-3′

FMRPS499A F: 5′-TGCTGCTGAAACAGAATCTGACCA-3′

FMRPS499D F: 5′-TGCTGATGAAACAGAATCTGACCA-3′

FMRPSalI R: 5′-GTGTCGACAGGGTACTCCATTCACCA-3′

#### Primary hippocampal neuron culture

Fourteen-day pregnant FVB mice were purchased from the National Laboratory Animal Center of Taiwan, and were used to prepare primary neuronal culture according to a standard procedure [44]. Procedures for mouse dissection and handling were approved by the relevant authority of NCKU, Tainan (IACUC number 110194). Primary hippocampal neuron culture was maintained in a 37 ℃ incubator containing 5% CO_2_ for 3 weeks. For brief mGluR activation and to establish mGluR-mediated LTD, DIV14 primary hippocampal cultures were subjected to 50–100 µM DHPG (Sigma) treatment for 30 s to 1 min (brief DHPG treatment) [53] or 100 µM DHPG treatment for 5–7 min (DHPG-LTD) [54], respectively. Co-treatments with 100 nM okadaic acid (OA, Abcam) for 30 s, 2.5 µM ionomycin (Sigma) for 10 min, or 10 mM EGTA (Merck, Germany) for 15 min were also performed occasionally along with DHPG treatment of the primary hippocampal neurons. Occasionally, neuronal culture was treated with 10 µM CX-4945 (Sigma) for 12 h.

For RNAi knockdown, DIV12 cultured hippocampal neurons were transfected with TDP-43 RNAi oligo (TDPsi, Ambion), FMRP RNAi oligo (FMRPsi, Sigma), or control oligo (Sc, Dharmacon) using Lipofectamine 2000 (Invitrogen) according to a previously described protocol [44]. These siRNAs were used several times before with minimum or no off-target effects [44, 55]. After the transfection and treatment procedures described above, primary neuron cultures were subjected to FISH/IF according to a standard protocol [44] described below. Occasionally, synaptosome extracts were also prepared from these neurons using SynPer reagent (Thermo Scientific, USA) following the user manual, and these were used to carry out IP and RNA-IP experiments according to procedures described previously [43], and also summarized below.

Primary hippocampal neurons grown on coverslips were co-transfected with 2 µg of pRFP-TDP-43 and 2 µg of pGFP-FMRP (WT), pGFP-FMRP (S499A), or pGFP-FMRP (S499D) using Lipofectamine 2000 (Invitrogen) according to a standard protocol [44]. Forty-eight hours (48 h) after transfection, the cells were fixed before performing fluorescence microscopy.

For live cell imaging, primary neurons transfected with pRFP-TDP-43 were incubated with 10 nM live cell FISH probe against *Rac1* mRNA for ~ 16 h prior to starting the imaging experiment. For TRICK reporter RNA experiments, 12-day primary hippocampal neurons were co-transfected with pPCP-GFP (Addgene, U.S.A.), pMCP-RFP (Addgene, U.S.A.), and pTRICK-*Rac1* 3′ UTR. Forty-eight hours (48 h) after transfection, the transfected cells were used for live cell imaging according to a previously described procedure [43].

#### HEK293T cell culture

HEK293T cells were bought from ATCC and stored. They wre cultured in DMEM supplemented with 10% fetal bovine serum (FBS) and penicillin/streptomycin solution in a 37 ℃ incubator containing 5% CO_2_ and transfected with pRFP-TDP-43 and pGFP-FMRP (WT), pGFP-FMRP (S499A), or pGFP-FMRP (S499D). Total proteins were isolated from the transfected HEK293T cells using lysis buffer containing 150 mM sodium chloride, 1.0% Triton X-100, 0.5% sodium deoxycholate, 0.1% SDS, and 1% protease inhibitor cocktail, before conducting IP analysis as described below.

#### Establishment of VPA-ASD mouse model and tissue sectioning

The pregnant FVB mice (IUPAC approval no. 110194) were maintained in the usual light/dark cycle. These mice were intraperitoneally injected twice with either two doses of VPA (Sigma) solution, each of 300 mg/kg in 0.9% of NaCl (saline), or with only saline solution at gestational day 11 and day 13, respectively, following instructions described in previous literature [56]. The volume of injection was kept below 10 mL/kg. Mice were dissected at gestational age 16.5 days to isolate the embryos. Freshly harvested embryo brains were fixed in 10% formalin, processed for paraffin embedding, sectioned at thickness of 5 microns, and mounted on slides. Finally, immunohistochemical analysis was done as described below.

From a different set of VPA-injected and control embryos, primary hippocampal neuron cultures were prepared. Synaptosomal mRNAs were prepared from DIV 12 primary neurons and analyzed by quantitative reverse-transcription polymerase chain reaction (qRT-PCR). When needed, the control and VPA-treated primary neurons at DIV 12 were treated with DMSO (mock), DHPG for 30 s (DHPG), or DHPG for 5 min (DHPG-LTD). Cells were then fixed, and IF staining was carried out. pGFP-actin transfected neurons were also sometimes used for characterization of the spine.

#### Immunohistochemical staining

Mice sections were deparaffinized with xylene and rehydrated. Sections were next pretreated with citrate buffer and formic acid for 20 min at 95 °C to enhance immune reactivity. Primary antibodies included anti-TDP-43 (1: 400), FMRP (1:250), and pFMRP (1:300) followed by probing with Alexa Fluor 488-, 555-, and 647-conjugated secondary antibodies followed by cover slipping with VECTASHIELD Anti fade-DAPI mounting medium (Vector Laboratories). During confocal microscopy, we randomly selected different regions from hippocampus to take images for further analysis.

#### RNA-IP and IP assay

Detailed procedures for the RNA-IP and IP assays were described previously [43, 44]. In brief, the synaptosome extracts or cell lysates were incubated with anti-TDP-43 (GenezTex), anti-FMRP (Milipore), anti-pMRP(S499) (Abcam) [57], anti-myosin V (Cell Signaling), anti-cortactin (Santa Cruz Biotechnology), anti-drebrin (Abcam), anti-GFP (Proteintech), or control IgG antibodies (Jackson Laboratories) at 4 ℃ overnight to immunoprecipitate the RNA–protein complexes. Next, agarose beads (GE Healthcare) were added to the mixture to pull down the RNA–protein complexes. RNAs were extracted from the complexes using TRIZOL (Invitrogen). Real time RT-PCR was conducted using primers specific to *Rac1* mRNA [43].

For IP analysis, the pulled-down protein complexes were extracted from the agarose beads by heating at 100 ℃ using sample buffer (50 mM Tris–HCl pH 6.8, 2% SDS, 10% glycerol, 12.5 mM EDTA, 0.147 M β-mercaptoethanol, and 0.02% bromophenol blue), and they were separated by 10–12% SDS-PAGE, transferred to membranes, and analyzed by Western blotting using anti-FMRP (Abcam), anti-phospho-FMRP (phospho S499) (Abcam), or as indicated in the figures.

#### FISH/IF assay

RNA FISH was carried out using Alexa 488-conjugated 5′-TTGACTGGTTCATTGGTTCA-3′ (Life Technologies, Japan). The design of the *Rac1* RNA FISH probe is detailed in our previous publications [43, 44]. In short, it was designed using a program on the Biosearch Technology website, and was tested by using OLIGOWALK and mFold software. The specificity of *Rac1* mRNA FISH probe was previously demonstrated using corresponding sense RNA probe [44]. Simultaneous imaging of *Rac1* mRNA by FISH and proteins by IF staining was carried out using standard protocols described in the Biosearch Technology manual. The FISH probes and antibodies, e.g., anti-TDP-43 (Proteintech), anti-myosin V (Cell Signaling), anti-RPL6 (Santa Cruz Biotechnology), anti-KIF5A (Millipore), anti-FMRP (Milipore), or anti-pFMRP (S499) (Abcam) [57], anti-αTubulin (Proteintech), or F-actin (Abcam), were incubated with the neurons overnight at 37 ℃. The cells were then further incubated with AlexaFluor 350-, AlexaFluor 488-, AlexaFluor 546-, and/or AlexaFluor 647-conjugated secondary antibodies (Invitrogen). FISH-IF images were acquired by LSM780 (Zeiss, Germany) confocal microscopy equipped with a 63× oil immersion objective lens and analyzed by using Zen2010 (Zeiss, Germany), Metamorph (Molecular Devices), and Imaris software. In all cases, corresponding differential interference contrast (DIC) images were also taken and compared with the fluorescence channels to identify localization of proteins and RNA in dendrites and spine regions. The approximate 3-µm region in the dendrites across the spine were considered as spine base regions. Beyond these spine bases, dendritic regions without spine regions were considered as nonbase dendritic regions. Dendrites with strong fluorescence intensities and taken from a similar neuron arbor were exemplified, as well as considered for different analysis modules. As also observed by others [58, 59], mRNP granules are heterogeneous in size owing to the presence of different RBPs.

Co-localization percentages of the confocal images representing the overlapping area of two or more fluorescence channels representing different protein/mRNA puncta intensities above the threshold within the dendritic spine regions of primary hippocampal neurons were obtained using Zen 10 software and are presented as the average co-localization percentage from 25–30 puncta. Meanwhile, co-localization analyses of high-resolution images are presented as the number of co-localized particles within the mRNP granules in the spines (granule size within 200–800 nm), as compared with the total number of co-localized particles within the 10 µm length of dendrites by using Imaris 9.1 software.

To determine the dendrite and spine localization and boundaries, DIC images of the same field view were used to identify and draw the spine and dendrite boundaries on fluorescence images of primary hippocampal neurons. Note that, while the expression and localization of GFP-actin or other proteins tagged with fluorophore have been used to categorize the spine types or measure different substructures of the spines, DIC images are also used frequently to identify and localize the spines or the spine-like structures [60, 61]. On some occasions, spine and dendritic regions identified using DIC images were further confirmed by GFP-actin expression and localization in the same hippocampal neurons.

### Puromycin assay

DIV14 primary hippocampal neurons in culture were treated with 10 µM puromycin (Sigma) for 3–5 min to detect the translating *Rac1* mRNP in situ. Following the standard fixation protocol for IF-RNA FISH co-staining with anti-TDP-43 (Proteintech), anti-puromycin (a gift from Dr. Yen), and *Rac1* FISH RNA probes (Invitrogen), the association between *Rac1* mRNA and puromycin was quantified using high-resolution imaging (3D-SIM, Zeiss). *Rac1* mRNA granules associated with puromycin are the translating ones [62].

To minimize the effects of ribosome dissociation from mRNA that could give false positive result [63], we set the threshold for the analysis of the sizes of *Rac1* mRNP granules within 200–800 nm. The interdistance between proteins or protein–RNA was also quantified.

#### Live cell imaging

Time-lapse imaging experiments were carried out using a spinning-disc confocal microscope (Nikon, Japan) and described in a previous publication [43]. It is equipped with a 647-nm solid-state laser (Cobolt), a 561-nm solid-state laser (Cobolt), and a 488-nm solid-state laser (Coherent) for simultaneous imaging of more than one fluoresce channels. A Nikon 60× or 100× 1.4NA objective lens was used. During experiments, cells were maintained at 37 °C and 5% CO_2_ (FC-5N CO_2_ controller) inside of an LCI TC chamber (Korea). The autofocus system was turned on during the recording of time-lapse images.

Live cell FISH of Rac1 mRNA was performed using a beacon harboring Cy5-conjugated 5′-GCUCAUUGACUGGUUCAUUGGUUC A[BtndT]GAGC-BHQ3-3′ with a nuclease-resistant backbone and specifically binding to endogenous *Rac1* mRNA [43]. The beacon was introduced by incubating neuron cells with a 50 nM probe containing medium at 37 °C for approximately 16 h.

The TRICK biosensor consists of three plasmids: pTRICK-*Rac1* 3′-UTR, pPCP-GFP, and pMCP-RFP. pTRICK-*Rac1* 3′-UTR expresses a RNA transcript consisting of the orthogonal bacteriophage PP7 (12X) and MS2 (24X) repeat sequences, forming stem-loop structures within the coding sequence and 3′ untranslated region, respectively, followed by the 3′-UTR of Rac1 RNA downstream of MS2. pPCP-GFP and pMCP-RFP express GFP-tagged and RFP-tagged proteins capable of specifically binding at PP7 and MS2 sequences, respectively. This biosensor system has been used previously as an Rac1 reporter to follow transport and translation of Rac1 reporter RNA in dendrites and spine regions [43].

For tracking of TRICK-Rac1-3′-UTR reporter RNA granules, MCP-RFP and PCP-GFP co-movements were tracked by simultaneous excitation by two lasers. The criteria for assigning granules for tracking time-periods have been described in detail previously [43]. Imaris 9.1 software (Bitplane Science, South Windsor, CT) equipped with a manual tracking module was used to analyze the time-lapse movies.

To determine translation dynamics and for time of translation measurement, color changes of TRICK reporter granules from yellow or green (untranslating) to red (translating) were analyzed within a 3-min time period inside of the spine and within spine base regions. Approximately 50% or more (by area) change in color to red was considered as a translation event. To follow the translation dynamics, the number of translation evens were recorded every 10 s in mock and DHPG for 3 min and 2 min, respectively. To measure the time of translation, the average time taken for one translation event was calculated.

For tracking of the endogenous Rac1 mRNA along with RFP-TDP-43 and GFP-FMRP protein(s), experiments were carried out by simultaneous excitation with two or three lasers. Movie analysis and the criteria for assigning granule types were as described previously [43]. Multicolored co-localized granules were tracked to record their separation. As also observed by others [58, 59], mRNP granules are heterogeneous in size owing to the presence of different amount of RBPs. During cellular processes, e.g., translation and transport, the protein content of these granules keeps changing, resulting in alteration of their sizes. We always analyzed the granules with diameter below 800 nm.

It is worth noting that, although the optimal scenario would be the analysis of RFP-TDP-43/GFP-FMRP/*Rac1* mRNA transport dynamics from the same dendritic region before and after DHPG treatment, we analyzed/exemplified different dendritic regions to compare control (mock) and DHPG treatment. The reasons are the following:

1. We are endeavoring to analyze the specific phenomenon of a three-colored granule moving in a dendrite under mock conditions versus another under DHPG treatment (i.e., a three-colored granule dissociating, with the resulting red + white granule moving into the spine). It is very difficult to follow the same granule and satisfy all criteria, considering that the number of granules entering into spine upon DHPG treatment only increases by 20–25% compared with the mock condition.
2. Fluorophore signal intensity is highly dependent on how long it is exposed to the laser, thus if we selected the same dendritic region to analyze both mock and DHPG-treatment conditions, the fluorescence intensities could not be compared. Therefore, identification of three colored/two colored granules would be ambiguous.

For the same reasons, different dendritic regions were also selected to analyze and exemplify the transport/translation dynamics of the TRICK reporter system under mock and DHPG treatment conditions.

The mock and DHPG treatment schemes for DIV14 primary hippocampal neuron culture are as follows:
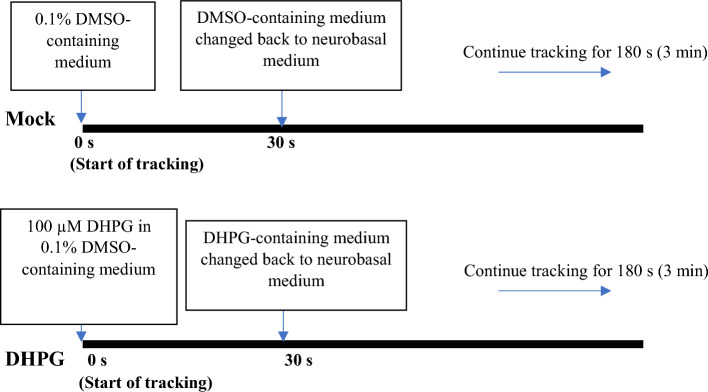


Similar treatment conditions were used for both live cell imaging and high-resolution microscopy of the neurons.

#### High-resolution 3D SIM microscopy

Three-dimensional (3D) structured illumination microscopy (SIM) was conducted using a Zeiss ELYRA PS.1 microscope equipped with a 63×/NA 1.4 oil (Plan-Apochromat, Zeiss) objective lens and iXon 885 EMCCD (Andor). TetraSpeck microsphere beads (0.1 µm, Thermo Fisher) were incubated with ProLong Gold (Thermo Fisher) anti-fade imaging reagent (dilution 1:200) as the internal control. Processing of SIM images to align different channel files and to determine the centroid coordination of the granules was performed using Zen software (Zeiss) on the basis of the internal control. To perform 3D SIM imaging of neurons and to remove background signal from reconstructed SIM images, we selected the following imaging parameters: five rotations, 43–57 *Z*-sections (110 nm step size), and suitable imaging threshold/brightness/contrast.

The high-resolution distance analysis [64, 65] of mRNP granules selected from the distal dendrites was carried out in a specific plane using Bitplane Imaris 10.0 software (Oxford Instruments). The measured distances of protein_RNA pairs > 800 nm were excluded from the analysis.

#### Spine density calculation

Mature (mushroom-like) spine characterization and density calculations were determined from pGFP-actin-transfected neurons [66]. Neurons with strong fluorescence intensity were chosen and included in the analysis to obtain a clear idea of the spine structures. They were further validated by corresponding DIC images. Definition of filopodia, mature spine, and mushroom-like spine follow standard definition from previous literature [67].

#### Statistical analysis

Microsoft Excel and Prism (GraphPad) were used to generate bar diagrams and pie charts. Statistical analyses were performed using GraphPad QuickCalcs (GraphPad Software, Inc., La Jolla, CA, U.S.A.) and Prism. Student *t*-tests were used to compare mean values. Student *t*-test results were further confirmed with Mann–Whitney test. The results of two statistical analysis were similar. One-way anlaysis of variance (ANOVA) was used to compare means from all different experimental conditions.

### Materials availability

Further information and requests of materials used in this research should be directed to Dr. Pritha Majumder (pritham@tmu.edu.tw). Plasmid DNA constructs generated in this study will be made available via material transfer agreement (MTA).

## Results

### TDP-43 is required for dendrite-to-spine transport of Rac1 mRNA in primary mouse hippocampal neurons under brief DHPG treatment

**Fig. 1:**
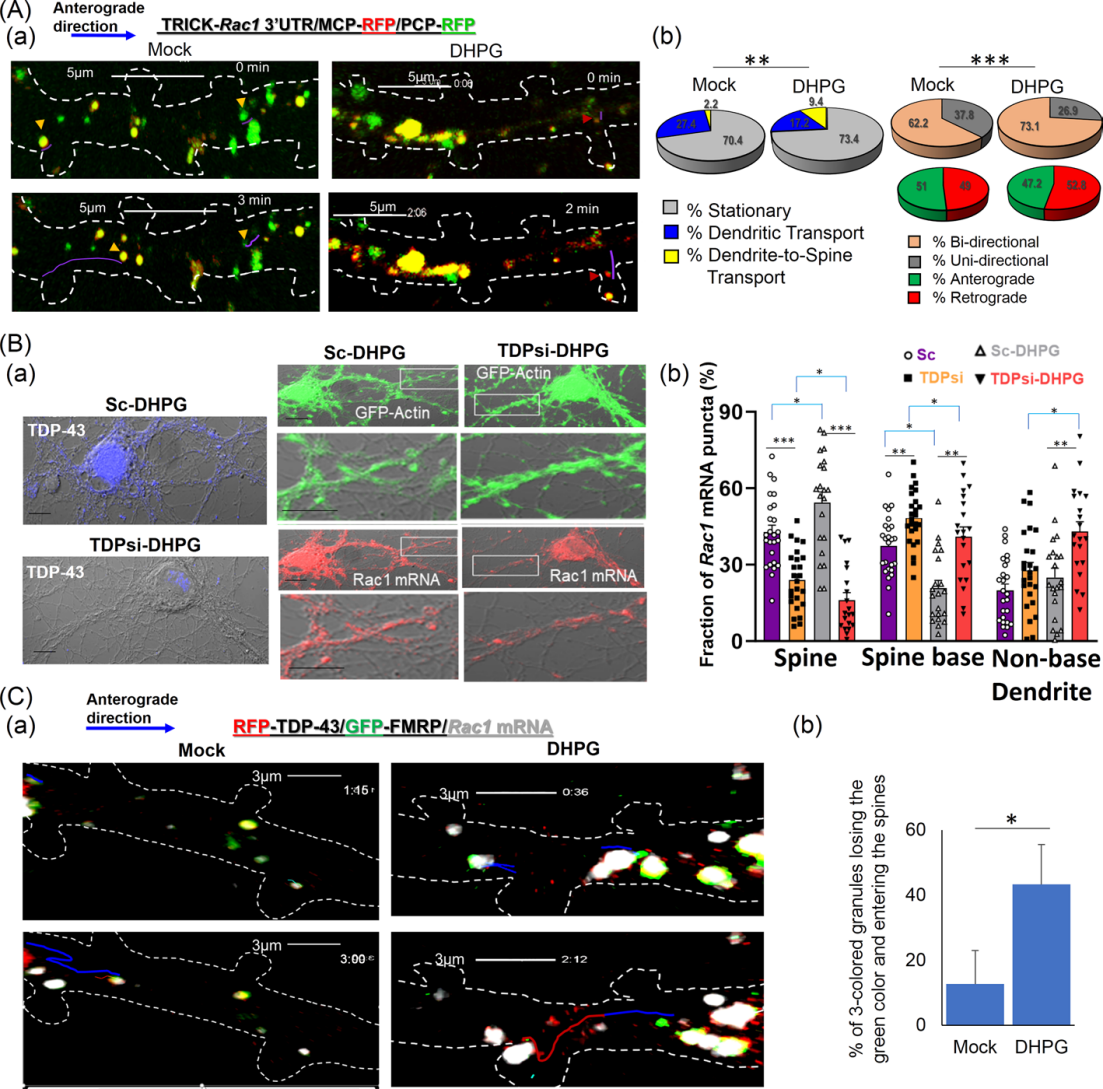
Neuronal activity-induced transport of dendritic *Rac1* mRNA into the spine requires TDP-43 and dissociation from FMRP. **A** Live cell imaging analysis of spine transport and translation of TRICK-*Rac1* 3′-UTR reporter RNA granules under conditions of mock treatment and DHPG treatment for 1 min (DHPG). (a) Representative time-lapse images of the spatial and temporal distributions of translating (red dots) and untranslating (green and yellow dots) reporter mRNA granules (arrowheads with purple tracking lines) in dendrites and spine of DIV14 primary hippocampal neurons. Boundaries of the dendrites and spines were determined from the corresponding differential interference microscopy (DIC) images (data not shown) and are represented by the white dotted lines. Scale bars, 5 µm. Transport and translation dynamics of the TRICK-*Rac1* 3′-UTR reporter RNA granules in the spine area is shown in Supplementary Fig. S1A and S1B, respectively. (b) Left, pie charts representing the proportions (%) of *Rac1* 3′-UTR reporter RNA granules that were stationary, moving in dendrites without entry into the spine, and moving from dendrite into the spine, respectively. Right top, the proportions (%) of dendritic *Rac1* reporter RNA granules that were moving bidirectionally or unidirectionally. Right bottom, the proportions (%) of unidirectionally moving dendritic *Rac1* reporter RNA granules in the anterograde and retrograde directions, respectively. Each set of data is derived from 30–45 granules (technical repeats, *n* = 30–45) from 25–30 dendrites analyzed in three independent experiments (biological repeats, *N* = 3). Student’s *t*-test was used to compare the pie charts, **p < 0.001, ***p < 0.0001. **B** IF/FISH analysis of TDP-43 and *Rac1* mRNA in DIV 14 primary hippocampal neurons co-transfected with pGFP-actin and different siRNA oligos followed by DMSO (mock) or DHPG treatment for 30 s. (a) Hippocampal neurons were subjected to RNA FISH by using *Rac1* mRNA-specific probes (red) and co-IF staining by using anti-TDP-43 (blue). The representative confocal microscopy images of TDP-43, *Rac1* mRNA, and GFP-actin in the dendrites and spine are shown in the same neuron cells here. Scale bars, 10 µm. Magnified dendritic spine region pictures are shown in Supplementary Fig. S1C. (b) Statistical analysis of the distributions of *Rac1* mRNA puncta in different compartments of the neurons. The data are derived from a total of 22–26 different dendritic regions (*n* = 22 to 26, shown as individual data points) from three sets of independent experiments (*N* = 3). Error bars represent SEM. Student’s *t*-test was used to compare different treatment conditions. **p < 0.001, ***p < 0.0001. One-way ANOVA was used to compare the distribution of Rac1 mRNA among different dendritic regions and found to be significant under all treatment conditions (***q < 0.0001, not shown in the figure). **C** Live cell imaging analysis of the transport of *Rac1* mRNA granules (white) and *Rac1* mRNAs associated with RFP-TDP-43 only (white + red) or with RFP-TDP-43 plus GFP-FMRP (white + red + green) in DIV14 primary hippocampal neurons co-transfected with pRFP-TDP-43 and pGFP-FMRP, and under the conditions of mock treatment or DHPG treatment, for 1 min. (a) Representative time-lapse images of the spatial and temporal distributions of white/(white + red)/(white + red + green) granules and their movement (arrowheads with purple tracking lines) in the dendrite or from dendrite into the spine in the hippocampal neurons. Boundaries of the dendrites and spines were determined from the corresponding DIC images (data not shown) and are represented by white dotted lines. Time of recording is 1′45″ (1′15″ to 3′00″) from Video V3 and 1′48″ (0′36″ to 2′12″)″ from Video V4. Scale bars, 3 µm. Note that snapshots from video file representing the mock condition were flipped to represent the anterograde movement. Three-colored granule dissociation is further clarified by presenting magnified images of the granules as well as adding more time point snapshots from Supplementary Video V4 in Supplementary Fig. S1D, E. (b) Bar diagram showing the statistical analysis of the proportion of three colored GFP-FMRP/RFP-TDP-43/*Rac1* mRNA granules in the spine regions that lost the green color (GFP-FMRP) and entered the spine under different experimental conditions. Each set of data is derived from 18–35 granules (*n* = 18–35) from 20–25 dendritic regions analyzed in three independent experiments (*N* = 3). Student’s *t*-test was used to compare different types of treatment conditions, **p < 0.001, ***p < 0.0001. Note that ~ 15–20% of total granules lost the green color but did not enter into the spine in DHPG-treated cells. This number is similar to the number of granules that lost green color after DHPG treatment in the medium (data not shown), and thus indicate a loss of fluorescence due to exposure to the laser. In the mock condition, this number is quite low, under 10%. Interestingly, loss of fluorescence of RFP is much slower

In a previous study, TDP-43, in cooperation with FMRP and Stau1, was found to regulate trafficking of *Rac1* mRNA across hippocampal neuron dendrites [43]. As an extension of that study, we employed the TRICK biosensor system that was used to monitor first round of translation of an open reading frame (ORF) and translation inhibition in presence of puromycin or cyclohexamide [68]. The details of the TRICK biosensor are described in the “Methods” section. Here we used this system to examine the effect of grp1 mGluR agonist DHPG treatment for a brief (~ 30 s to 1 min) period of time on spine trafficking and translation of *Rac1* reporter mRNA in DIV 14 primary hippocampal neurons. Before the live cell imaging, the locations of the dendrites and spine areas as well as their boundaries were identified using corresponding DIC images as done before by us [43] and by other groups [60, 61]. As shown in Fig. 1A, Supplementary Table 1, and Supplementary Videos V1 and V2, TRICK-*Rac1* 3′-UTR RNA granules (yellow, green, and red) exhibited dendritic transport in both the anterograde and retrograde directions in control (mock) cells and cells under brief DHPG-treatment (DHPG). However, under brief DHPG treatment, bidirectional transport of the RNA granules increased significantly [top-right set of pie charts, Fig. 1A(b)], but their dendritic transport velocity and net displacement in the anterograde direction decreased relative to the mock control (Supplementary Table 1). We also detected a significant increase in dendrite-to-spine transport (9.4% in DHPG compared with 2.2% in mock) of TRICK-*Rac1* reporter RNA granules in DHPG-treated neurons, with a concomitant decrease in movement of such granules that remained within dendrites and did not enter spine (17.2% in DGPG compared with 27.4% in mock) (Figs. 1A(b) and S1A). Thus, results described in Fig. 1A(b) and Supplementary Table 1 together demonstrated an altered transport dynamic under brief DHPG treatment with decreased anterograde movement and increased bidirectional movement that presumably would be the preparation phase for the *Rac1* mRNA granules to pause and finally enter into the spines.

Next, we followed the fate of untranslating TRICK-*Rac1* reporter RNA granules (yellow and green) near the spine base and in the spine region. As expected from previous research [43], live cell imaging analysis showed that the RNA granules in both mock and DHPG-treated neurons paused for some time at the spine base before they were transported into the spine. However, DHPG treatment for a brief time period resulted in a significant increase in the proportion of granules pausing near the spine base in conjunction with a decrease in the average pausing time of the granules before entering the spine (Supplementary Table 2). Interestingly, brief DHPG treatment also caused a 1.5- to 3.5-fold increase in the average number of translation events (yellow untranslating reporter RNA granules to become red translating ones; Supplementary Fig. S1B). As previously observed for stationary-state neurons [43], endogenous TDP-43 was also required for dendrite-to-spine transport and spine localization of *Rac1* mRNA in neurons under DHPG treatment (Fig. 1B). For this study, pGFP-actin transfected neurons were used to identify the spines and related substructures of dendritic spine regions. Clearly visible spines as well as endogenous *Rac1* mRNA (red) from Sc control neurons and TDP-43 depleted neurons are exemplified in S1C.

We found that the spine localization of *Rac1* mRNA was reduced approximately twofold by RNAi-mediated depletion of TDP-43 [compare the purple and yellow bars, in the left set of histobars, Fig. 1B(b)]. Upon DHPG treatment for 30 s, spine localization of *Rac1* mRNA increased by ~ 20% in control Sc oligo-transfected neurons [compare the purple and grey bars in the left set of histobars, Fig. 1B(b)]. However, depletion of TDP-43 by RNAi oligo prominently shifted the localization of *Rac1* mRNA in DHPG-treated neurons toward the spine base and non-spine base dendritic regions [exemplified in Fig. 1B(a) and statistically analyzed in Fig. 1B(b)].

We also analyzed co-trafficking of endogenous *Rac1* mRNA as detected by a *Rac1* RNA beacon (for details, please see the Methods section), GFP-tagged FMRP, and RFP-tagged TDP-43 proteins in DIV14 primary neurons co-transfected with pEGFP-FMRP and pRFP-TDP-43 constructs. As depicted in Fig. 1C(a) and Supplementary Videos V3 and V4, three-colored granules (red, TDP-43; green, FMRP; white, *Rac1* mRNA) trafficked predominantly across the dendrites in control (mock), as well as brief DHPG-treated (DHPG), neurons. Interestingly, upon dissociation of these three-colored granules near the spine base, the white-only (*Rac1* mRNA) and white/red co-localized (*Rac1* mRNA + TDP-43) granules moved into the spine, whereas the green-only granules remained at the spine base, where they either stalled or moved further in the anterograde direction. To further clarify the dissociation of three-colored granules and to compare granule colors, magnified images of three-colored, two-colored, and green granules as well as the internal time points from Supplementary Video V4 are shown in Supplementary Fig. S1D, E. Notably, DHPG treatment increased the extent of dissociation of the three-colored granules by ~ twofold (Supplementary Table 3), which resulted in a ~ 3.5-fold increase of the percentage of granules entering into the spine after losing the green color (Fig. 1C(b)).

Together, the data in Fig. 1 illustrate a pivotal role of TDP-43 in regulating dendrite-to-spine transport of *Rac1* mRNA in activated neurons. Moreover, immediate mGluR1/5 activation of the neurons would lead to dissociation of TDP-43 and bound *Rac1* mRNA from FMRP and, presumably, from the FMRP-associated dendritic transport complex, as well [43].

### Dephosphorylation of FMRP acts as a molecular switch to promote dendrite-to-spine transport of TDP-43-associated *Rac1* mRNA

#### Phosphorylated FMRP is the favored interaction partner of TDP-43

**Fig. 2:**
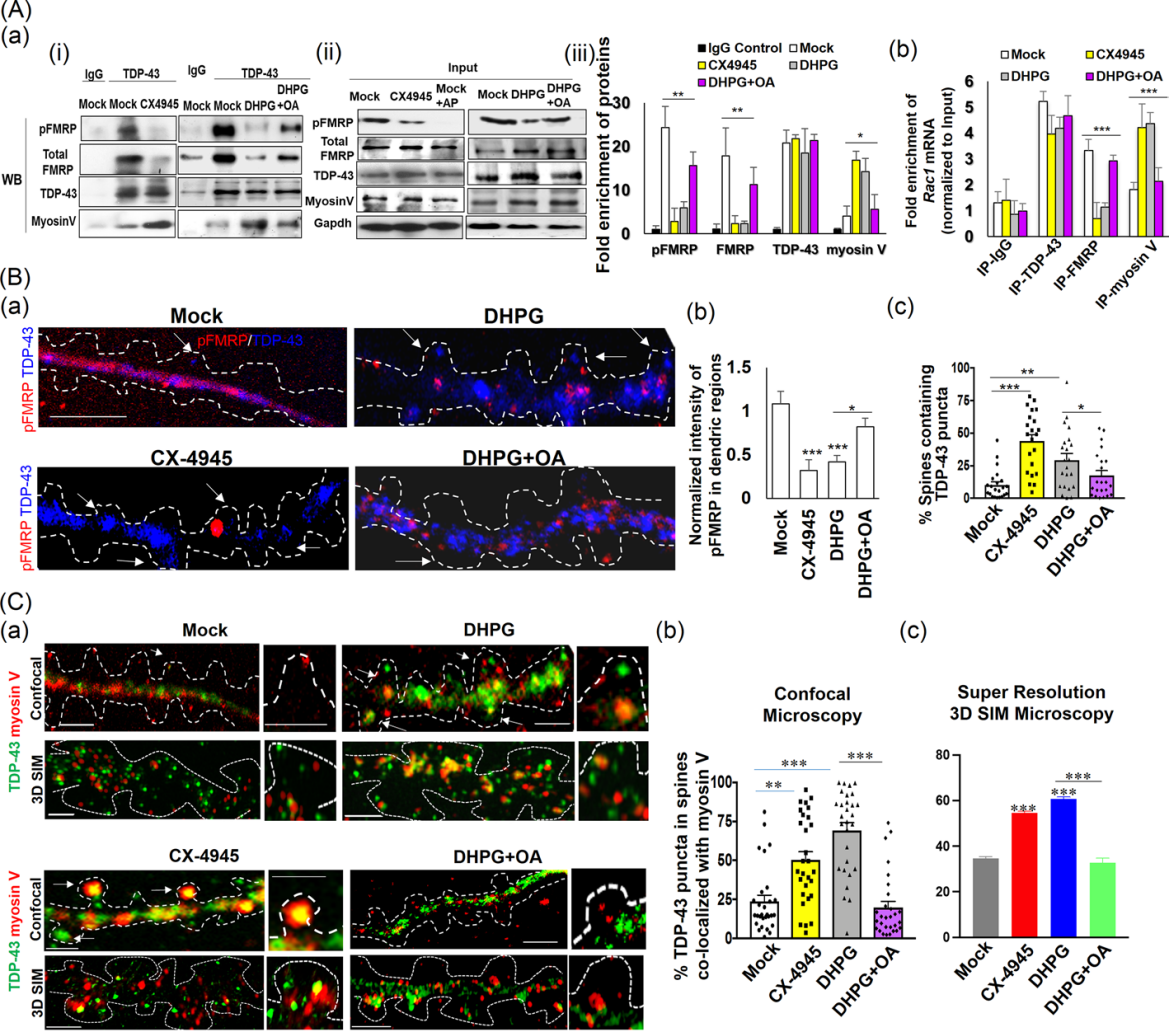
Dephosphorylation of pFMRP (S499) results in the dissociation of TDP-43/*Rac1* mRNP from FMRP and association with myosin V inside the spines. **A** IP and RNA-IP analysis of synaptosome extracts isolated from DIV14 primary hippocampal neurons treated with DMSO (mock), DHPG, CX-4945, or (DHPG + OA). (a) (i) IP analysis. Synaptosome extracts are immunoprecipitated with anti TDP-43, and the immunoprecipitated proteins are analyzed by Western blotting (*N* = 3) to identify associations of pFMRP, total FMRP, and myosin V proteins, respectively, with TDP-43; (ii) Input. Synaptosome extracts are directly analyzed by Western blotting to identify the levels of the indicated proteins without any further treatment. In some cases, extracts from mock condition were treated with alkaline phosphatase (mock + AP). Note that specificity of anti-pFMRP has been established by comparing mock and mock + AP lanes. (iii) Quantification of IP analysis. The bar diagram shows the average fold of enrichment of pFMRP, total FMRP (phosphorylated plus unphosphorylated forms), TDP-43, and myosin V proteins in the TDP-43-IP fractions relative to the IgG control and normalized with input. Error bars represent SEM (*N* = 3). (b) RNA-IP analysis. The synaptosome extracts are immunoprecipitated with different antibodies, and the immunoprecipitated RNAs are analyzed by qRT-PCR using *Rac1* mRNA-specific primers. The normalized values (with respect to the inputs and amount of protein precipitated in each condition) are presented as fold enrichment relative to the IgG-mock sample (*N* = 3). One-way ANOVA was used to compare different treatment conditions; *q < 0.05, **q < 0.001, ***q < 0.0001. **B** Spine localization of TDP-43 in relation to the levels of pFMRP in the dendrites of DIV 14 primary hippocampal neurons under different conditions. (a) Representative confocal microscopy images of co-IF staining using anti-TDP-43 and anti-pFMRP are shown with arrows pointing to TDP-43 protein puncta inside of the spine. Scale bars, 5 µm. (b) Bar diagrams representing the relative average dendritic intensities of pFMRP under different treatment conditions. (c) Statistical analysis of the proportions of spine containing TDP-43 puncta. The data are derived from three sets of independent experiments (*N* = 3) and include a total of 21–28 different spine-containing dendritic regions (*n* = 21–28, shown as the individual data points). Error bars represent SEM. **C** Co-localization of TDP-43 and myosin V in spine of DIV 14 primary hippocampal neurons under different experimental conditions. (a) Representative confocal microscopy images (scale bars, 2 µm) and 3D SIM high-resolution microscopy images (scale bars, 2 µm) are shown with the arrows pointing to TDP-43/myosin V co-localized puncta inside of the spine. Magnified images of specific spine regions are shown (scale bars, 2 µm) beside the low-magnification images of the dendrites. (b, c) Statistical analysis of the co-localization (%) of TDP-43 and myosin V in the spine, respectively. The data consist of a total of 26–28 different spine-containing dendritic regions (*n* = 21 to 28), shown as individual data points in (b) derived from three sets of independent experiments (*n* = 3). Error bars represent SEM. Student’s *t*-test was carried out to compare the means. *p < 0.05, **p < 0.001, ***p < 0.0001. One-way ANOVA was used to compare all different treatment conditions (***q < 0.001, not shown in the figure). Dendrites and spine regions represented as white dotted lines in (**B**) and (**C**) are predicted from corresponding DIC images (data not shown)

We have previously shown that association of TDP-43-bound *Rac1* mRNA with FMRP is required for anterograde transport, as well as for translational inhibition of *Rac1* mRNA, in neuronal dendrites [43]. Even though it has been well established that TDP-43 physically interacts with FMRP in neurons [41, 69], whether any specific posttranslational modification(s) of FMRP are required for this interaction has been unknown. Since neurons possess an abundant level of FMRP phosphorylated at residue Ser499 (pFMRP) by the constitutively active kinase CK2 [53], first we examined if TDP-43 is associated with unphosphorylated FMRP or pFMRP. Immunoprecipitation (IP) of synaptosome extracts prepared from hippocampal neurons indicated that TDP-43 indeed associates with pFMRP (S499) [top left panel, Fig. 2A(a, i)]. Furthermore, treatment of neurons with the compound CX-4945, a known inhibitor of CK2 [54], selectively reduced the level of pFMRP, but not those of total FMRP, TDP-43 [Fig. 2A(a, ii)], or phosphorylated TDP-43 (pTDP-43) (data not shown).

Interestingly, IP and RNA-IP analysis of synaptosomal extracts from mock and CX-4945-treated neurons further uncovered reductions in the association of TDP-43 with pFMRP/FMRP (Figs. 2A(a) and S2A) and the association of *Rac1* mRNA with FMRP, but not with TDP-43 (Fig. 2A(b)), as found for inhibition of FMRP phosphorylation by CX-4945. Since *Rac1* mRNA associates with FMRP through TDP-43 [44], we anticipated that the ~ sixfold reduction in the association of FMRP with *Rac1* mRNA in CX-4945-treated samples (Fig. 2A(b)) is attributable to the impaired interaction between FMRP and TDP-43 proteins under the same condition.

#### TDP-43/*Rac1* mRNA are associated with myosin V upon CX-4945-mediated reduced phosphorylation of FMRP

How are TDP-43-bound mRNAs, such as *Rac1* mRNA, transported into the dendritic spine? The spine contain branches of actin filaments with mature spine formation requiring active transport of mRNAs, proteins, and vesicles into the spine that is facilitated by myosin proteins, including the actin-based motor protein myosin V [70]. As shown by IP and RNA-IP analysis, myosin V was associated with TDP-43 (Fig. 2A(a)) and *Rac1* mRNA (Fig. 2A(b)), but not with FMRP (data not shown), in synaptosomal extracts from DIV 14 hippocampal neurons. Moreover, the association between myosin V and *Rac1* mRNA was significantly decreased in TDP-43-depleted neurons, indicating a TDP-43-dependent association of this mRNA with myosin V (Supplementary Fig. S2B, C). Furthermore, we detected an increased association of myosin V with TDP-43 (Figs. 2A(a) and Supplementary Fig. S2A) and *Rac1* mRNA (Fig. 2A(b)) in synaptosomal extracts from CX-4945-treated neurons relative to the mock controls.

These data indicate that CK2 inhibition causes dephosphorylation of pFMRP and dissociation of TDP-43 and bound mRNAs from FMRP. This in turn promotes the association of the TDP-43/mRNA complexes with actin-based transporter myosin V. Our IF analysis results described below further support this mechanism (Fig. 2B, C). Before that, in the immediate next section, we demonstrated the effect of FMRP phosphorylation at S499 on the above-mentioned mechanism and dendrite-to-spine entry of TDP-43-bound mRNPs.

#### Effects of phosphomimetic and dephosphomimetic FMRP mutants on dendrite-to-spine entry of TDP-43

**Fig. 3:**
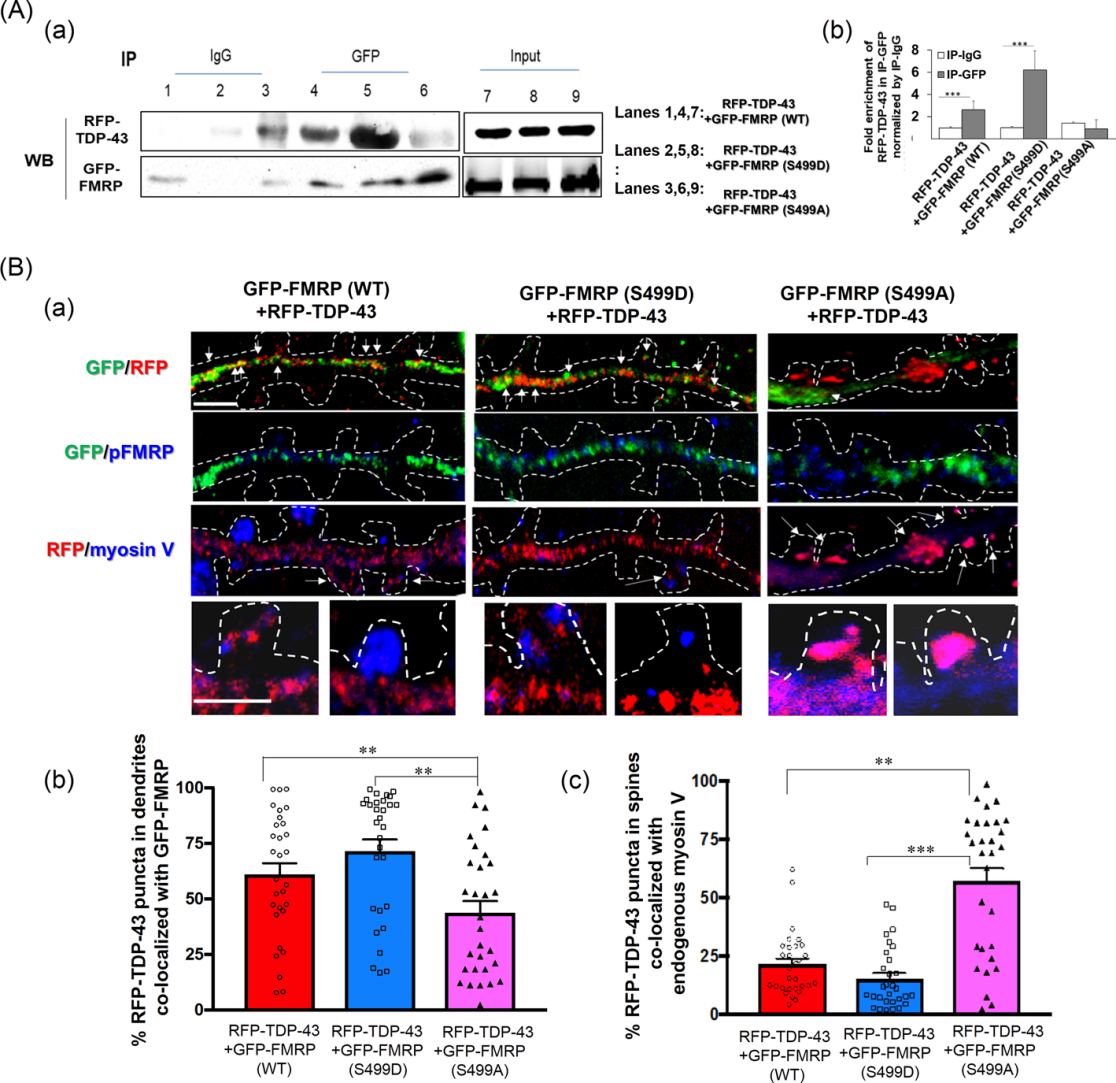
Mutation of the phosphorylation site S499 of FMRP alters its association with TDP-43 and subsequent spine localization of the TDP-43 granules. **A** IP analysis using anti-GFP was carried out with HEK293T cell lysates co-expressing RFP-TDP-43 plus GFP–FMRP (WT), GFP–FMRP (S499D), or GFP–FMRP (S499A) followed by Western blotting using anti-RFP and anti-GFP. (a) Left, representative Western blotting patterns showing the differential association of TDP-43 with FMRP (WT), phosphomimetic FMRP (S499D), and dephosphomimetic FMRP (S499A), respectively. Input panels on the right show the expression levels of different exogenous proteins in the transfected HEK293T cells. (b) Bar diagram from three independent IP experiments using anti-GFP showing the average fold enrichment of RFP-TDP-43 protein relative to the IgG control after normalization with amount of GFP-FMRP. Error bars represent SEM. Student’s *t*-test was performed to compare the mean with the corresponding IgG control and indicated as ***p < 0.0001. One-way ANOVA was used to compare fold enrichment of RFP-TDP-TDP-43 in Wt and mutant GFP-FMRP-expressing cells, *q < 0.05 (not shown in the figure). **B** Co-localization of RFP-TDP-43 with GFP-tagged WT or mutant FMRP proteins in the dendrites and with endogenous myosin V protein in the spine, respectively, of DIV 14 primary hippocampal neurons. The co-IF analysis was performed using anti-RFP, anti-GFP, and anti-myosin V. (a) Representative confocal microscopy images showing the co-localizations of RFP-TDP-43 puncta with WT GFP-FMRP and mutant GFP-FMRP in the dendrites or with myosin V in the spine, as indicated by the arrows. Magnified pictures of specific spine regions are shown in the lower panels. Scale bars, 2 µm. White dotted lines represent the boundaries of the dendrites and spine regions as determined from the corresponding DIC images shown in Supplementary Fig. S9A. Note that, in primary neurons co-expressing GFP-FMRP(S499A) and RFP-TDP-43, dendritic transport of TDP-43 granules in the anterograde direction was severely restricted presumably due to loss of the association between TDP-43 and FMRP [43]. For unbiased comparison, the dendritic spine regions near soma were selected for analysis under all three experimental conditions. (b, c) Statistical analysis of the co-localization (%) represented by the bar diagrams. The data are derived from three sets of independent experiments (*N* = 3). Technical repeats, *n* = 26–30 dendritic and spine regions. Error bars are SEM. Student’s *t*-test was carried out to compare the means and indicated as **p < 0.001, ***p < 0.0001. One-way ANOVA was used to compare Wt and different mutants, *q < 0.05 (a), ***q < 0.0001 (b), (not shown in the figure)

To validate and understand the role of the phosphorylation status of FMRP in spine transport of TDP-43 and its associated *Rac1* mRNA, we conducted site-directed mutagenesis on FMRP to generate FMRP (S499D) (phosphomimetic) and FMRP (S499A) (dephosphomimetic) mutant proteins. Plasmids expressing GFP-tagged FMRP (WT), FMRP (S499A), or FMRP (S499D) were used for cell transfection. IP analysis (Fig. 3A) of extracts prepared from HEK293 cells co-transfected with pRFP-TDP-43 plus pGFP-FMRP (WT), pGFP-FMRP (S499A), or pGFP-FMRP (S499D) revealed enhanced or impaired interactions of RFP-TDP-43 protein with the FMRP (S499D) and FMRP (S499A) mutant proteins, respectively. The same sets of plasmids were co-transfected into primary hippocampal neurons. As anticipated, the dendritic co-localization of the exogenous RFP-TDP-43 with dephosphomimetic GFP-FMRP (S499A) was lower than that with the wild-type GFP-FMRP or with the phosphomimetic GFP-FMRP (S499D) [exemplified in top panels of Fig. 3B(a) and quantified in B(b)]. Overexpression of the dephosphomimetic mutant of FMRP also enhanced co-localization of the exogenous RFP-TDP-43 with myosin V in the spine compared with both the wild-type and the phosphomimetic mutant (Fig. 3B(c)). Interestingly, there were no significant differences between the wild-type GFP-FMRP and the phosphomimetic GFP-FMRP (S499D) in either of these two analyses. This is ascribed to the fact that Wt GFP-FMRP protein becomes phosphorylated by CK2 in 14 DIV primary hippocampal neurons.

Therefore, it appears that phosphorylation of FMRP at S499 is required for its interaction with TDP-43. In the absence of FMRP phosphorylation, TDP-43 and its bound mRNAs are dissociated from FMRP and likely from the dendritic transport, translation complexes associated with FMRP [43] as well.

#### Brief DHPG treatment causes dephosphorylation of pFMRP (S499), dissociation of TDP-43/Rac1 mRNA from FMRP complex, and subsequent association of TDP-43- mRNA cargo with myosin

Brief DHPG treatment-mediated immediate mGluR1/5 stimulation is known to activate PP2A, a phosphatase that dephosphorylates pFMRP (S499) [54]. To validate the role of the phosphorylation status of FMRP in the dendrite-to-spine transport of TDP-43/*Rac1* mRNA granules, we treated primary hippocampal neurons at DIV14 with DHPG for ~ 30 s. Subsequent IP analysis showed that the synaptosomal extract from the brief DHPG-treated neurons indeed had a lower level of pFMRP [top right panel, Fig. 2A(a)(ii)], whereas total FMRP remain constant, and impaired association of TDP-43 with pFMRP/FMRP (Figs. 2A(a) and S2A) compared with the control (mock). Interestingly, upon this treatment, the association of *Rac1* mRNA with FMRP was reduced, and the association of myosin V with TDP-43, as well as with *Rac1* mRNA, was significantly increased (Figs. 2A(a) and A(b)). As expected, the extents of these DHPG-induced changes could be reduced by simultaneous treatment with the PP2A inhibitor okadaic acid (OA) [see Ref. [71] and Fig. 2A].

#### The reduction of pFMRP (S499) level in primary neuron dendrites results in dissociation of TDP-43/Rac1 mRNA granules from FMRP and promotes the subsequent dendrite-to-spine transport of TDP-43/*Rac1* mRNA granules associated with myosin V

As anticipated from the above biochemical study by IP/RNA-IP, immunofluorescence (IF) imaging analysis (Fig. 2B) revealed a significant decrease in pFMRP level in the dendrites and an increase in the number of TDP-43 puncta-containing spines in CX-4945-treated primary hippocampal neurons at DIV14 [compare the left two bars, Fig. 2B(b) and (c)]. Interestingly, IF analysis using confocal and high-resolution 3D structured illumination microscopy (3D SIM) indicated that the increased association of TDP-43 and its bound *Rac1* mRNA with myosin V in CX-4945-treated neurons was accompanied by translocation of the TDP-43/myosin V complex [left panels of Fig. 2C(a), left two bars of (Fig. 2C(b) and (c)] and, presumably, the TDP-43-bound *Rac1* mRNA as well into the spine. IF staining analysis of the endogenous TDP-43/myosin V puncta (Supplementary Fig. S3) and live cell imaging analysis of exogenous RFP-TDP-43/endogenous *Rac1* mRNA (Supplementary Videos V6 and V7 and Supplementary Table 4) further showed an increase in spine trafficking of TDP-43/myosin V co-localized granules and TDP-43/*Rac1* mRNA granules, respectively, upon CX-4945 treatment. Finally, IF analysis of DHPG-treated neurons confirmed the aforementioned biochemical data with the same treatment reducing the level of pFMRP protein in dendrites [exemplified in Fig. 2B(a) and quantified in Fig. 2B(b)] and increasing the proportion of spine with TDP-43 puncta (Fig. 2B(c)), as well as the proportion of co-localized TDP-43/myosin V granules in the spine (Fig. 2C), respectively. Again, co-treatment with OA and DHPG significantly rescued these DHPG-mediated changes in the neurons (Fig. 2B, C).

The above data together provide evidence in favor of a mechanism involving short-term DHPG treatment-mediated dephosphorylation of pFMRP (S499) by PP2A and consequent dissociation of FMRP from the TDP-43-bound mRNAs. These TDP-43/mRNA complexes are then free to associate with myosin V and translocate into the dendritic spine.

### Long DHPG treatment preserves TDP-43/FMRP-kinesin 1 puncta and facilitates their spine transport along with associated *Rac1* mRNA in a calcium-dependent mechanism

**Fig. 4:**
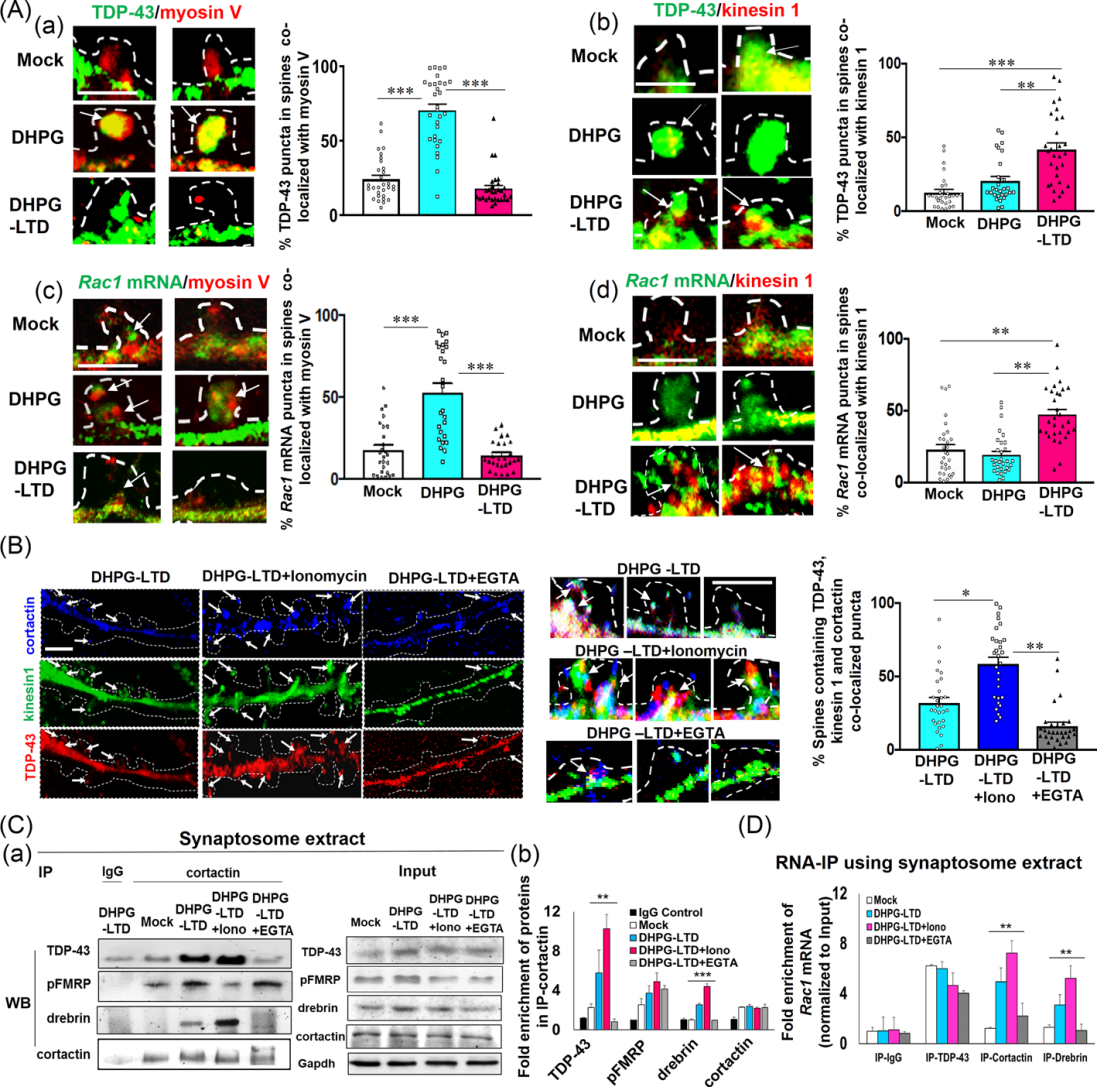
DHPG-induced LTD facilitates cortactin/drebrin-assisted and Ca^2+^-dependent entry of the TDP-43/*Rac1* mRNA/FMRP-kinesin 1 complex into dendritic spine. **A** Co-localization among different proteins and *Rac1* mRNA in the spine of DIV 14 primary hippocampal neurons under mock, brief DHPG (DHPG), and long DHPG (DHPG-LTD) treatment conditions. Co-IF analysis was carried out using (a) anti-TDP-43 and anti-myosin V; (b) anti-TDP-43 and anti-kinesin 1. Representative confocal microscopy images of two spine regions with co-localization (the arrows) of TDP-43 with myosin V or kinesin 1, respectively, are shown. Hippocampal neurons were also subjected to RNA FISH by using probes specific for *Rac1* mRNA and co-IF staining by using anti-myosin V (c) and anti-kinesin 1 (d). Representative confocal microscopy images of two spine regions with co-localization (the arrows) of *Rac1* mRNA with myosin V or kinesin 1, respectively, are shown. Scale bars, 2 µm. Spine regions are magnified from representative confocal microscopy images of the dendrites shown in Supplementary Fig. S5A–D. Boundaries of the dendrites and spine were determined from the corresponding DIC images, also shown in Supplementary Fig. S5. Statistical analyses of the co-localization (%) from 26–28 dendritic spine regions (*n* = 26–30, shown as individual data points) from three sets of independent experiments (*N* = 3) are represented by the bar diagrams beside each set of spine image panels. **B** Co-localization of cortactin, kinesin 1, and TDP-43 in the spine of DIV 14 primary hippocampal neurons after long DHPG (DHPG-LTD) treatment only, long DHPG treatment followed by ionomycin (DHPG-LTD + Iono), or long DHPG treatment followed by EGTA (DHPG-LTD + EGTA). Hippocampal neurons were subjected to co-IF staining using antibodies against the indicated proteins. Representative confocal microscopy images show the distributions of different proteins in the dendrites and spine. The arrows indicate the spine containing the individual proteins. Magnified images of specific spine are shown in the right panels (Merged) with the arrows indicating puncta displaying co-localization of the three proteins. Boundaries of the dendrites and spine were determined from the corresponding DIC images (data not shown). Scale bars, 2 µm. Statistical analysis of the percentage of spine with co-localizations of the three proteins is represented in the bar diagram beside the image panels (*N* = 3; *n* = 25–28 dendrites shown as individual data points). Student’s *t*-test was carried out to compare the means in (**A**) and (**B**). *p < 0.05, **p < 0.001, ***p < 0.0001. One-way ANOVA was used to compare all different treatment conditions (***q < 0.0001, not shown in the figure). The involvement of kinesin 1 in the transport of *Rac1* mRNA into the spine under DHPG-LTD is similar to the functional role of kinesin 3 in driving the neuronal activity-dependent spine entry of syt-IV-associated vesicles [77]. **C** IP analysis of synaptosome extracts isolated from DIV14 primary hippocampal neurons under conditions of mock, DHPG-LTD, (DHPG-LTD + Iono), or (DHPG-LTD + EGTA). (a) Synaptosome extracts are immunoprecipitated with anti-cortactin, and the pulled-down proteins are analyzed by Western blotting (*N* = 3) to identify the association of cortactin with TDP-43, pFMRP, and drebrin, respectively. Input panels show the levels of different proteins in the synaptosome extracts used in the IP assay. (b) Quantification of the IP analysis. The bar diagram shows the average fold of enrichment of TDP-43, pFMRP, drebrin, and cortactin proteins in the cortactin-IP fractions relative to the IgG control after normalization with input. Error bars represent SEM (*N* = 3). **D** RNA-IP analysis of synaptosome extracts isolated from DIV14 primary hippocampal neurons after the indicated treatments. Synaptosome extracts were immune-precipitated with different antibodies, and the immune-precipitated RNAs were analyzed by qRT-PCR using *Rac1* mRNA-specific primers. The normalized values (with respect to the input and amount of protein precipitated in each condition) are presented as fold enrichment relative to the IgG-mock sample (*N* = 3). Error bars represent SEM. One-way ANOVA was performed to compare different treatment conditions in (**C**) and (**D**), and indicated as **q < 0.001, ***q < 0.0001

Next, we investigated how *Rac1* mRNA is transported into the spine of hippocampal neurons in culture during long DHPG treatment mimicking gp1 mGluR-mediated LTD [72]. Continuation of DHPG treatment beyond 1 min activates the mGluR1/5-mTORC1 pathway, leading to S6 kinase (S6K)-mediated rephosphorylation of FMRP [73] that had been initially dephosphorylated by PP2A during brief DHPG treatment [71]. Moreover, prolonged activation of mGluR reduces the electrical impulses in hippocampal neurons over an extended timeframe, diminishing synaptosome generation and initiating structural LTD [13, 74]. In accordance with these studies, our western blotting (WB) analysis of synaptosome extracts from primary hippocampal neurons showed an increase in pERK1/2, but not total ERK1/2, following 5 min DHPG treatment (DHPG-LTD) compared with the brief treatment of about 30 s (DHPG) as well as to the mock condition, indicating the activation of ERK signaling cascade during DHPG-LTD condition (Supplementary Fig. S4A). IF (Supplementary Fig. S4B) and WB (Supplementary Fig. S4A) analysis further revealed an increased level of pFMRP (S499), but not of total FMRP, in DIV14 primary hippocampal neuronal dendrites under DHPG-LTD compared with brief DHPG treatment. However, no significant difference in pFMRP level was detected between mock and DHPG-LTD conditions. Furthermore, co-localization of TDP-43 with FMRP inside of the spine was also elevated under long DHPG treatment (Supplementary Fig. S4C). However, in contrast to brief DHPG treatment, the proportions of TDP-43 or *Rac1* mRNA puncta co-localizing with myosin V inside the spine declined (Fig. 4A(a) and A(c), Supplementary Figs. S5A and S5C). Instead, co-localization of TDP-43 and *Rac1* mRNA puncta with FMRP-associated microtubule motor protein kinesin 1 inside the spine increased significantly under the DHPG-LTD condition when compared with mock and brief DHPG-treated neurons [Fig. 4A(b) and A(d) and Supplementary Fig. S5B, D]. Interestingly, average *Rac1 mRNA* intensity in the spines increased from 72.4 ± 10.4 AU/spine in mock condition to 206.3 ± 26.3 AU/spine under brief DHPG treatment, and to 158.3 ± 12.4 AU/spine under DHPG-LTD condition. These results along with PSD-95 staining (data not shown) further demonstrated transport of *Rac1* mRNP granules in the post-synaptic compartments of the DHPG–treated neurons.

Moreover, before dendritic-to-spine transport of myosin V/*Rac1* mRNA*/*TDP-43 granules, the myosin V intensity inside the spines was 173.6 ± 10.7 AU under mock. It increased by ~ 2.5 fold following brief DHPG treatment. In contrast, the kinesin 1 intensity in the spine was quite low, 26.4 ± 3.7 in mock condition, that is, increased by ~ 3.6 fold following dendrite-to-spine entry of kinesin1/FMRP/TDP-43/*Rac1* mRNA complex in the spines under DHPG-LTD. For further confirmation of spine transport complex formation between TDP-43 and myosin V under brief DHPG treatment and between TDP-43 and FMRP under DHPG-LTD, the high-resolution 3D SIM technique was used. Brief and long-term DHPG treatment resulted in an average distance between TDP-43 and myosin V of ~ 350 nm and between TDP-43 and FMRP of less than 200 nm, respectively, indicating complex formation between these proteins under DHPG treatment (see Supplementary Fig. S5E and Ref. [65]). It is interesting to note that presence of microtubule motor protein kinesin 1inside the spine with TDP-43 and Rac1 mRNA under DHPG-LTD indicated a probable microtubule invasion in the spine. Time-dependent infiltration of dendritic microtubules inside the spines under DHPG treatment beyond 2 min as well as accumulation of FMRP puncta inside the spines under DHPG-LTD (Supplementary Fig. S6A, B) demonstrated occurrence of microtubule invasions in the spines during DHPG-LTD. Noteworthily, though microtubule invasion in the spines were previously reported under global neuronal activation stimulation and spine transport of vesicles [75, 76], the current report is, most probably, the first to evidence spine transport of mRNA through this mechanism.

It was previously shown that, during spine trafficking of vesicles, microtubule invasion is often followed by actin remodeling involving actin binding proteins, e.g., cortactin and drebrin [77]. This prompted us to study any involvement of these proteins in spine transport mechanism of TDP-43/*Rac1* mRNA. Since the release of cellular calcium is extremely crucial for mGluR-mediated LTD as well as required for spine actin dynamics [78, 79], we planned to investigate the effects of cellular Ca^2+^ concentration on the association of cortactin/drebrin with TDP-43, kinesin 1, pFMRP, and with RNA. Interestingly, co-localization of TDP-43, kinesin 1, and the actin-remodeling protein cortactin inside of the spine increased upon co-treatment of the neurons with ionomycin, the Ca^2+^ ionophore known to increase intracellular Ca^2+^ levels, but decreased under co-treatment with the Ca^2+^ chelator EGTA (Fig. 4B). Furthermore, as revealed by IP (Figs. 4C and Supplementary Fig. S6C) and RNA-IP (Fig. 4D) analyses of synaptosomal extracts, associations of TDP-43 with cortactin, cortactin with another actin-binding protein drebrin, and cortactin/drebrin with *Rac1* mRNA, respectively, all increased significantly under long DHPG treatment (DHPG-LTD) of the DIV 14 mouse primary hippocampal neurons, when compared with the mock control. Our IF, IP, and RNA-IP data also showed that associations of the above-mentioned proteins with TDP-43/*Rac1* mRNA were elevated upon co-treatment with ionomycin, but declined under co-treatment with EGTA (Fig. 4B–D). Therefore, co-localization of TDP-43/kinesin 1/cortactin in the spine and association of TDP-43/*Rac1* mRNA with kinesin 1/cortactin/drebrin in synaptosome extracts from hippocampal neuron culture under the DHPG-LTD condition are regulated by a Ca^2+^-dependent mechanism. Moreover, as revealed by IP analysis, the association of TDP-43 with cortactin and kinesin1 (Fig. 4C) and presumably *Rac1* mRNA and drebrin as well (Fig. 4D) also requires presence of FMRP in the system (Supplementary Fig. S6D).

These molecular and microscopy data collectively suggest that the spine entry of TDP-43-bound *Rac1* mRNA associated with pFMRP and the microtubule motor protein kinesin 1, as well as the actin-binding proteins cortactin and drebrin, is driven by the long DHPG treatment, which mimics mGluR1/5-mediated LTD, and it is dependent on the cellular Ca^2+^ level. Interestingly, the NMDAR-mediated spine transport of vesicles in neurons is also regulated by actin remodeling-dependent microtubule entry into the spine [79, 80].

### Translational reactivation of *Rac1* mRNA transported into the spine under short-term DHPG, but not long-term DHPG, treatment

**Fig. 5:**
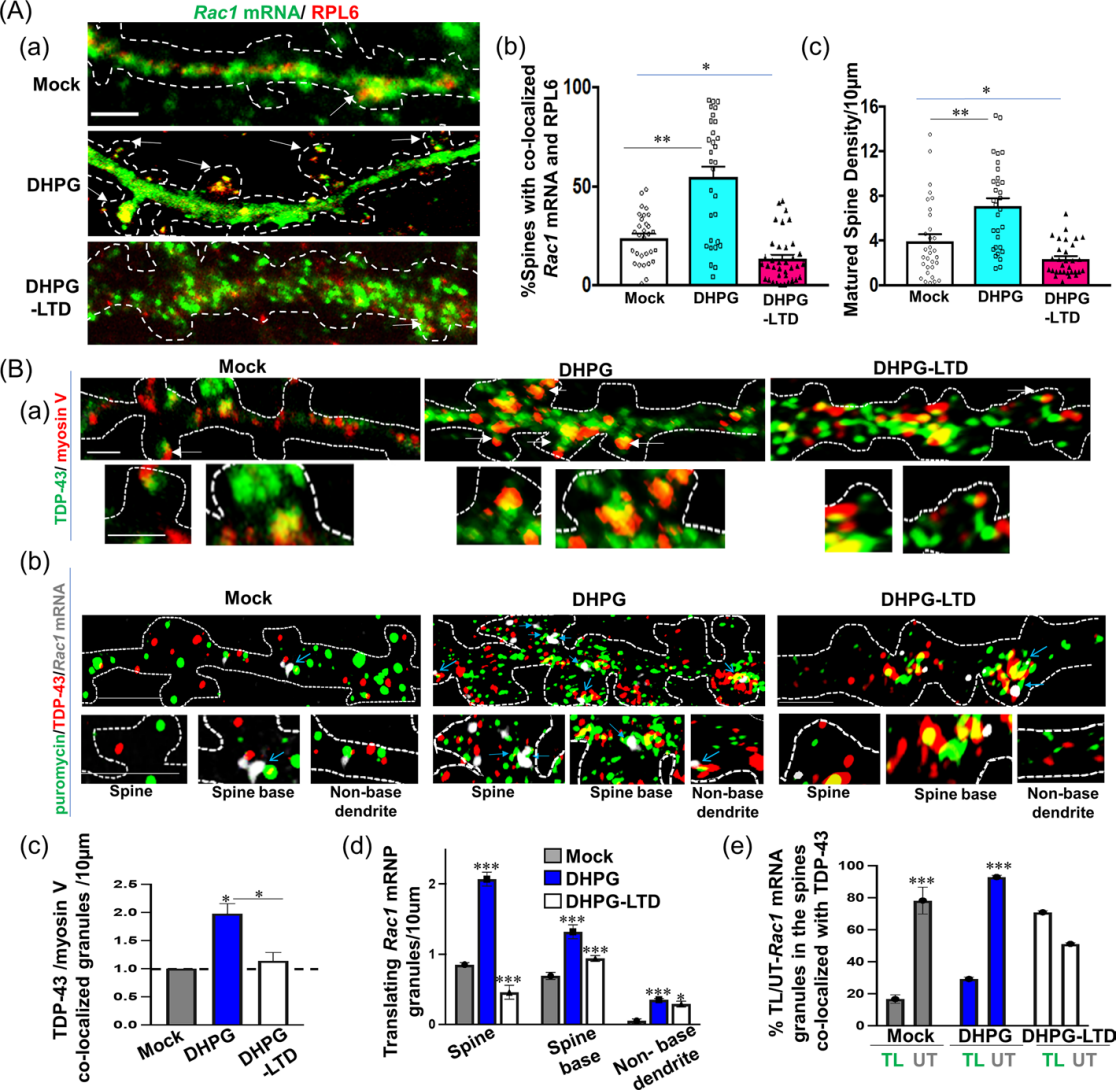
*Rac1* mRNAs are translationally active after being transported into the spine of short-term DHPG-treated, but not of long-term DHPG-treated, hippocampal neurons. **A** Co-localization analysis of the ribosomal protein RPL6 with *Rac1* mRNA in the spine of DIV 14 primary hippocampal neurons under mock, DHPG treatment for brief period (DHPG), and long DHPG (DHPG-LTD) treatment conditions, respectively. Hippocampal neurons were treated with DHPG and subjected to RNA FISH using *Rac1* mRNA-specific oligo probe and co-IF using anti-RPL6. (a) Representative confocal microscopy images showing the co-localization of *Rac1* mRNA with RPL6 inside of the spine as indicated by the arrows. Scale bars, 2 µm. (b) Statistical analysis of the proportion (%) of spine containing *Rac1* mRNA/RPL6 protein co-localized puncta. (c) Bar diagram comparing the numbers of mature spines per 10 µm length of the dendrites. Error bars represent SEM. The data were derived from three sets of independent experiments (a total of 28–32 dendrites were analyzed in each of the treatment conditions). Student’s *t*-test was carried out to compare the means and indicated as *p < 0.05, **p < 0.001. One-way ANOVA was carried out to compare different treatment conditions. ***q < 0.0001, not shown in figure. For identification of the spines, neurons were transfected with pGFP-actin followed by different treatments. Representative dendritic spine pictures under mock, brief DHPG treatment, and DHPG-LTD are shown in Supplementary Fig. S9C. As also exemplified in Supplementary Fig. S1C, GFP-actin protein accumulation inside the spines helped to identify and quantify the spines and filopodia. **B** Co-localization analysis of TDP-43 with myosin V and with untranslating (UT, not associated with puromycin) or translating (TL, associated with puromycin) *Rac1* mRNA in the spine of DIV 14 primary hippocampal neurons under mock, DHPG treatment for a brief period (DHPG), and long-term DHPG (DHPG-LTD) treatment conditions followed by co-treatment with puromycin. (a) Primary hippocampal neurons were subjected to IF analysis using anti-TDP-43 (green) and anti-myosin V (red). Representative 3D SIM high-resolution microscopy images are shown with arrows indicating TDP-43/myosin V co-localized puncta inside of the spine. Magnified images of representative spine are shown in the lower panels. (b) Primary hippocampal neurons were subjected to IF/FISH analysis using anti-TDP-43 (red), anti-puromycin (green), and a *Rac1* mRNA probe (grey). Instances of *Rac1* mRNA with puromycin are shown by solid arrows indicating translating *Rac1* mRNA (grey + green) only, and co-localized puncta of TDP-43 with translating *Rac1* mRNA (red + grey + green) are shown by open arrowheads inside of the spine, at the spine bases, and in the non-spine base regions. Magnified images of representative spine, spine bases, and non-spine base regions are shown in the bottom panels. Scale bars, 2 µm. Statistical analysis from 18–46 granules from three independent experiments is shown as the fold change in density of TDP-43/myosin V co-localized granules in the spine (c), the density of translating *Rac1* mRNA granules in the spine/spine base/non-spine base dendrite regions (d), and the co-localization (%) of TDP-43 with TL or UT *Rac1* mRNA granules in the spine (e). The data are derived from two or three sets of independent experiments (*N* = 3). Technical repeats, *n* = 18–27 dendrites. Error bars represent SEM. Student’s *t*-test was carried out to compare the means and indicated as *p < 0.05, ***p < 0.0001

We examined the fate of *Rac1* mRNPs transported into the dendritic spine under conditions of short- and long-term DHPG treatments to mimic immediate mGluR1/5 activation-mediated potentiation and prolonged activation of mGluR causing long-term depression (LTD), respectively. As shown in Fig. 5A, IF and fluorescence in situ hybridization (FISH) assays revealed a significant increase in the proportion of spine containing *Rac1* mRNA and 60S ribosome-binding protein RPL6 co-localized granules in 30 s DHPG-treated neurons (DHPG) compared with control (mock). Since RPL6 was shown previously to be associated with polysomes in primary hippocampal neurons [44], the data presented in Fig. 5A(c) indicate an increase in the number of spine containing translating *Rac1* mRNAs under the brief DHPG treatment. In contrast, DHPG-LTD treatment did not increase the proportion of spine hosting *Rac1* mRNA/RPL6 co-localized granules (Fig. 5A(a) and A(b)), implying that the *Rac1* mRNA transported into the spine under this condition remained in a translationally silent state. Note that RPL6 amount in the cells did not change significantly as depicted from the IF staining.

To further identify actively translating *Rac1* mRNA, we treated the primary hippocampal neurons with puromycin, which binds to stalled ribosomes at the elongation stage, and then deployed IF/FISH with anti-puromycin antibody to label the actively translating mRNAs (Fig. 5B). High-resolution 3D SIM microscopy revealed that the proportions of TDP-43 (green)/myosin V (red) co-localized granules [exemplified in Fig. 5B(a), quantified in B(c)] as well as puromycin (green)/*Rac1* mRNA (red) co-localized granules [exemplified in Fig. 5B(b), quantified in B(d)] in the spine increased significantly under transient activation of neurons following brief DHPG treatment, but they declined in DHPG-LTD treated neurons compared with mock controls. Therefore, the spine transport of *Rac1* mRNAs associated with myosin V under 30 s DHPG treatment is followed by their translation inside of the spine, thereby enhancing the density of mature spine for these DHPG-treated neurons (Fig. 5A(c)). In contrast, spine transport of *Rac1* mRNA under the DHPG-LTD condition occurred without the involvement of myosin V (Fig. 4A). The mRNA also remained translationally silent, likely owing to the presence of the FMRP-associated translation inhibitory complex. These results were further confirmed by staining analysis of Rac1 protein in the spines and other dendritic substructures following DHPG treatments (data not shown).

Interestingly, a high-resolution microscopy analysis further showed that TDP-43 remained associated with 20–30% of the translating *Rac1* mRNPs and ~ 85% of the untranslating *Rac1* mRNPs, respectively, in the spine of both brief DHPG-treated and control (mock) neurons (Fig. 5B(e)), implying that TDP-43 dissociation from the *Rac1* mRNA might be a prerequisite for their active translation after being transported from the dendrites into the spine. Consistently, the high-resolution microscopy (3D SIM)-based distance measurement revealed that the average distance between TDP-43 and translating *Rac1* mRNA on the same plane was significantly higher compared with that with the untranslating *Rac1* mRNA (Supplementary Fig. S7A). In addition, sectional analysis by high-resolution microscopy showed that myosin V was also distant from the *Rac1* mRNA in the translating mRNPs, but not in the untranslating ones (Supplementary Fig. S7B). In contrast, the distance between *Rac1* mRNA and TDP-43 only changed slightly or remained unchanged within the mRNPs with or without myosin V association and under different treatment conditions (Supplementary Fig. S7C).

### Reduced level of FMRP phosphorylation at S499 and impaired dendrite-to-spine transport of *Rac1* mRNA under steady state (mock), brief mGluR1/5 potentiation, and mGluR-mediated LTD in a mouse model of ASD

Finally, we examined the role of phosphorylation–dephosphorylation of FMRP in the grp I mGluR activation-mediated dendrite-to-spine transport of TDP-43-bound mRNAs, in particular *Rac1* mRNA in a maternally VPA-injected VPA-ASD mouse model. Thus far, mice with gene knock-out of Fmr1, Shank3, Mecp2, etc., or with exposure of their neonates to some chemicals as a result of maternal injection of drugs, e.g., VPA, are some of the most frequently used animal models for studies of ASD [81]. These animal models, although utilizing heterogeneous mechanistic pathways to establish the disease phenotypes, recapitulate many of the behavioral and pathophysiological changes related to ASD [31, 33].

**Fig. 6:**
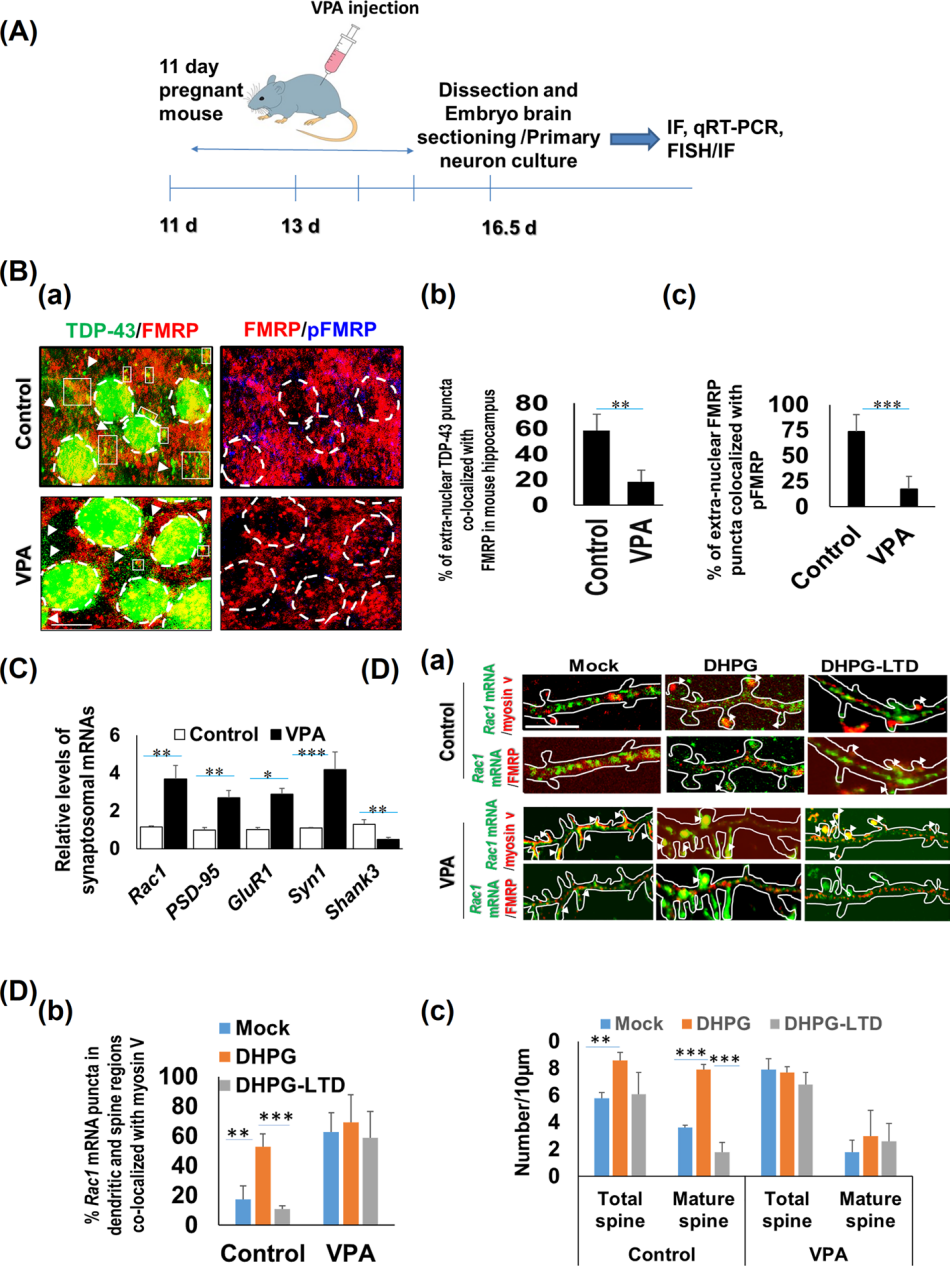
Maternal VPA injection-induced ASD-like molecular and cellular phenotypes in primary hippocampal neurons in culture. **A** Schematic diagram representing the time frame of VPA injections to the pregnant mice, brain tissue sectioning, and culturing of primary hippocampal neurons from the embryos for molecular and imaging analyses by qRT-PCR, IF, and IF/FISH. **B** Reduction in pFMRP level and accompanied decrease of the association between TDP-43 and FMRP in the hippocampus of mouse embryos from VPA-injected mother. Co-immunostaining was performed to analyze the co-localization patterns of TDP-43/FMRP and pFMRP/FMRP, respectively. Representative confocal microscopy images of IF are shown in (a) with the dotted lines to label the nuclei, the white boxes to indicate areas with co-localization of the TDP-43 and FMRP protein puncta, and arrow heads to indicate TDP-43 protein puncta not co-localized with FMRP in the cytoplasm. Scale bars, 5 µm. Statistical analyses are shown as the bar diagrams in (b) and (c), respectively. The data are derived from three sets of independent experiments (*N* = 3) and include a total of 10–15 different tissue sections (*n* = 10–15). Error bars represent SEM. Student *t*-test was used to compare the samples from control (Con) and VPA-injected mice; **p < 0.001, ***p < 0.0001. **C** Comparative analysis of the expression levels of *Rac1*, *PSD-95*, *GluR1*, *Syn1*, and *Shank3* mRNAs in primary hippocampal neurons cultured from saline (Con) or VPA-injected pregnant mice. Synaptosomal extracts isolated from DIV 12 neurons were subjected to qRT-PCR analysis (biological repeats, *N* = 3) using primers specific for the different mRNAs. The data are presented as fold change relative to the control after normalization with *Gapdh* mRNA levels (mean ± SD). Student’s *t*-test; *p < 0.05, **p < 0.001, ***p < *0.0001.* **D** Malfunctioning of the molecular switches controlling the dendrite-to-spine transport of mRNAs under different synaptic transmissions in the ASD model system. Primary hippocampal neurons cultured from embryos of the control and maternally VPA-injected mice were subjected to RNA FISH using probes specific for *Rac1* mRNA and co-IF staining using anti-myosin V or anti-FMRP. (a) Representative confocal microscopy images showing the co-localization (the arrows) of *Rac1* mRNA with myosin V and FMRP in the dendrite and spine regions of DIV 12 primary hippocampal neurons under mock, DHPG treatment for brief period (DHPG), and long DHPG (DHPG-LTD) treatment conditions, respectively. Scale bars, 5 µm. Boundaries of the dendrites and spine were determined from the corresponding DIC images (data not shown). Note that the spine of primary hippocampal neurons cultured from VPA-injected embryos are all long and with a very small head, which is indicative of immature ones compared with the matured mushroom-shaped spine with short stem and bigger head present mostly in control under immediate mGluR activation by brief DHPG treatment. Statistical analyses of the co-localization (%) between *Rac1* mRNA and myosin V (b), and numbers of total spine or mature spine per 10 µm length of the dendrites (c) are represented by the bar diagrams. Ten to 15 dendritic regions (*n* = 10 to 15) from three sets of independent experiments (*N* = 3) were analyzed. Identification and quantification of the spines were carried out mostly using the corresponding DIC images merged with *Rac1* RNA FISH and myosin staining. They were also confirmed by fluorescence patterns of GFP-actin as illustrated in Supplementary Figs. S1C and S9C. Error bars represent SEM. Student’s *t*-test was carried out to compare the means. **p < 0.001, ***p < 0.0001

First, following the time frame schematically represented in Fig. 6A, pregnant FVB mice were peritoneally injected with valproic acid (VPA) or saline water (control). The embryos were then subjected to brain tissue sectioning and IF analysis (Fig. 6B). Alternatively, primary hippocampal neurons from the embryo brains were cultured for further molecular, as well as imaging, analyses. Significantly, the association between TDP-43 and FMRP decreased by ~ 2.5-fold (Figs. 6B(a), B(b) and Supplementary Fig. S8A) as a result of reduction in the level of the cytoplasmic pFMRP (S499) in the hippocampal region of VPA-treated embryos [Fig. 6B(c) and Supplementary Fig. S8A, input]. Similar to previous research on hippocampal and cortical tissue samples from ASD models, including the VPA mice model [82], as well as from the patients [83], VPA treatment significantly increased the levels of *PSD-95* and *Syn1* mRNAs, but decreased that of *Shank3* mRNA (Fig. 6C; second, fourth, and fifth set of bars) in synaptosomal extracts of the primary neuron culture. Importantly, the maternal VPA treatment also increased amount of TDP-43 and FMRP co-regulating *Rac1* and *GluR1* mRNAs (Fig. 6C) and protein (Supplementary Fig. S8A, input, showing Rac1 protein) in the spine regions. Furthermore, in parallel to the data of Fig. 4A(c), primary hippocampal neuron culture from these VPA-treated embryos exhibited increased association, by approximately threefold, of *Rac1* mRNA with myosin V in the dendritic and spine regions compared with the control (Fig. 6D(a), D(b)). The above-mentioned FISH/IF data were also supported by RNA-IP analysis using synaptosomal extracts from primary hippocampal neurons of maternally VPA treated mouse embryos (Supplementary Fig. S8B), indicating dysregulation in the tight control of the mRNA transport/translation scheme in the primary hippocampal neurons of ASD mice. As a consequence, the total spine density significantly increased, while the mature spine formation was severely impaired [Fig. 6D(c); comparing the blue bars]. In marked contrast to the control, there were no significant differences in the association of *Rac1* mRNA with myosin V in the primary neurons cultured from VPA-treated mice embryos under mock, brief, and long DHPG treatment [Fig. 6D(b)]. Moreover, no significant changes in spine number or morphology were detected under these treatment conditions (Fig. 6D), indicating impairment of the mGluR-mediated signaling in the ASD mice. Is it possible to inhibit the spine transport of TDP-43/*Rac1* mRNA by increasing the proportion of phosphorylated FMRP in primary hippocampal neuron culture from VPA-ASD mice? To answer the question, we overexpressed Wt [GFP-FMRP(Wt)] or phosphomimetic mutant [GFP-FMRP(S499D)] of FMRP in DIV 10 primary hippocampal neurons from maternally VPA-treated mouse embryos. Interestingly, FMRP (Wt) and FMRP(S499D) overexpression could decrease the proportion of *Rac1* mRNA puncta co-localized with myosin V in spines by 1.5 fold and 2.4 fold, respectively (Supplementary Fig. S8C).

These data together indicate that the reduced S499 phosphorylation of FMRP and its dissociation from the TDP-43/*Rac1* mRNA complex leads to immature spine formation as well as nonresponsiveness of the molecular switch of dendrite-to-spine transport of *Rac1* mRNA, and likely other TDP-43-bound mRNAs, as well, toward grp1 mGluR-mediated neurotransmissions in the primary hippocampal neurons of the VPA-ASD model. It is interesting to note here that dysregulation of the mGluR-mediated LTD has previously been reported in other ASD animal models, as well as patients with ASD [84, 85], but the mechanisms behind it have been unknown. Grp1 mGluR-dependent signaling is also impaired in a range of neurodegenerative diseases including AD, Parkinson’s disease (PD), etc. [86]. Therefore, the study described in this manuscript has uncovered a new mechanistic pathway involving TDP-43, the misregulation of which would contribute to the pathophysiology of patients with ASD.

## Discussion

Focusing on *Rac1* mRNA encoding the spinogenesis-essential Rac1 protein, we have elucidated the regulatory mechanisms of TDP-43/FMRP complex-mediated switching of the fate of neuronal mRNAs from transporting across the dendrites to spine entry under immediate mGluR activation and under mGluR-mediated LTD, respectively. Aberration of the mGluR activation pathways is closely associated with neurological disorders, including ASD [86]. In particular, this aberration would lead to the alteration of synaptic plasticity, spine maturation, and neuronal connectivity [87]. Interestingly, the mGluR activation mechanism also links ASD with other neurological disorders, such as AD [88]. While neuronal activity-dependent spine translation of mRNAs inside of the spine is well documented [89–92], relatively little is known regarding the molecular switches that initiate the dendrite-to-spine transport of mRNAs upon mGluR-mediated neurotransmission, as well as the molecular mechanisms of spine entry of these mRNAs. Exploration of the above would provide new insights into the regulation of neuronal activities under physiological conditions and their misregulation in the disease state, in particular ASD.

In the present study, we used DHPG to activate grp1 mGluR pathways that increased pERK1/2 in primary hippocampal neurons (Supplementary Fig. S4A) and also turned on an “immediate early signaling cascade” resulting in PP2A-mediated dephosphorylation of pFMRP (Fig. 2Aa(ii)) [53]. Activation of mGluR1/5 by brief exposure to DHPG also induced the spine entry and translation of TDP-43-bound Rac1 mRNA in primary neurons (Fig. 1, Supplementary Videos V1, V2, V3, and V4, and Supplementary Tables 2 and 3) as a result of dephosphorylation of pFMRP (S499) (Fig. 2). Interestingly, the S499-phosphorylated form of FMRP turns out to be the preferred binding partner of TDP-43 since the dephosphomimetic mutant FMRP (S499A) exhibits remarkably reduced interaction with TDP-43, whereas the phosphomimetic mutant FMRP (S499D) displays a higher capability to associate with TDP-43 than its WT counterpart (Fig. 3; see Supplementary Fig. S9A for the corresponding DIC images of the dendrites and spine regions). In neurons, phosphorylation of FMRP is maintained by CK2, while brief DHPG treatment-mediated activation of PP2A causes dephosphorylation of pFMRP [54, 71]. Dephosphorylation of FMRP has been shown to impair its direct binding with certain mRNAs [53, 73] and also to inhibit its association with Dicer, thus activating miRNA biogenesis [93]. FMRP has also been implicated in carrying a group of its target mRNAs from the spine base into spine [29]. In contrast, base-to-spine transport of TDP-43-bound mRNAs, such as *Rac1* mRNA, seems to leave FMRP behind at the spine base (Fig. 1C, Supplementary Videos V3 and V4) [43] and enters into spines (Supplementary Videos V6 and V7; dendritic and spine region identification is exemplified in Supplementary Fig. S9B). The experimental evidence that we have presented herein demonstrates that DHPG treatment for a brief period leads to dephosphorylation of pFMRP by PP2A, followed by dissociation of TDP-43 and FMRP, resulting in the separation of TDP-43/*Rac1* mRNA from the dendritic FMRP-kinesin 1 transport complex. This enables the association of TDP-43 and bound mRNAs with the actin filament motor protein myosin V, resulting in the translocation of TDP-43-associated *Rac1* mRNA into the dendritic spines (Figs. 2, 3A, 4A(a), A(c), and Supplementary Fig. S2). Consistently, spine with polysome-associated *Rac1* mRNA, as well as the density of translating *Rac1* mRNA granules inside of the spine, are increased owing to the absence of FMRP-associated translation repressor complex FMRP/CYFIP1/eIF4G [44]. Consequently, the spine density as well as the proportion of mushroom-like spines also increased (Figs. 5, Supplementary Fig. S9C, D). Noteworthily, the myosin V-assisted translocation of RBP/mRNA cargo transport scheme described in this study is similar to the transport of mRNPs associated with TLS protein [18].

**Fig. 7:**
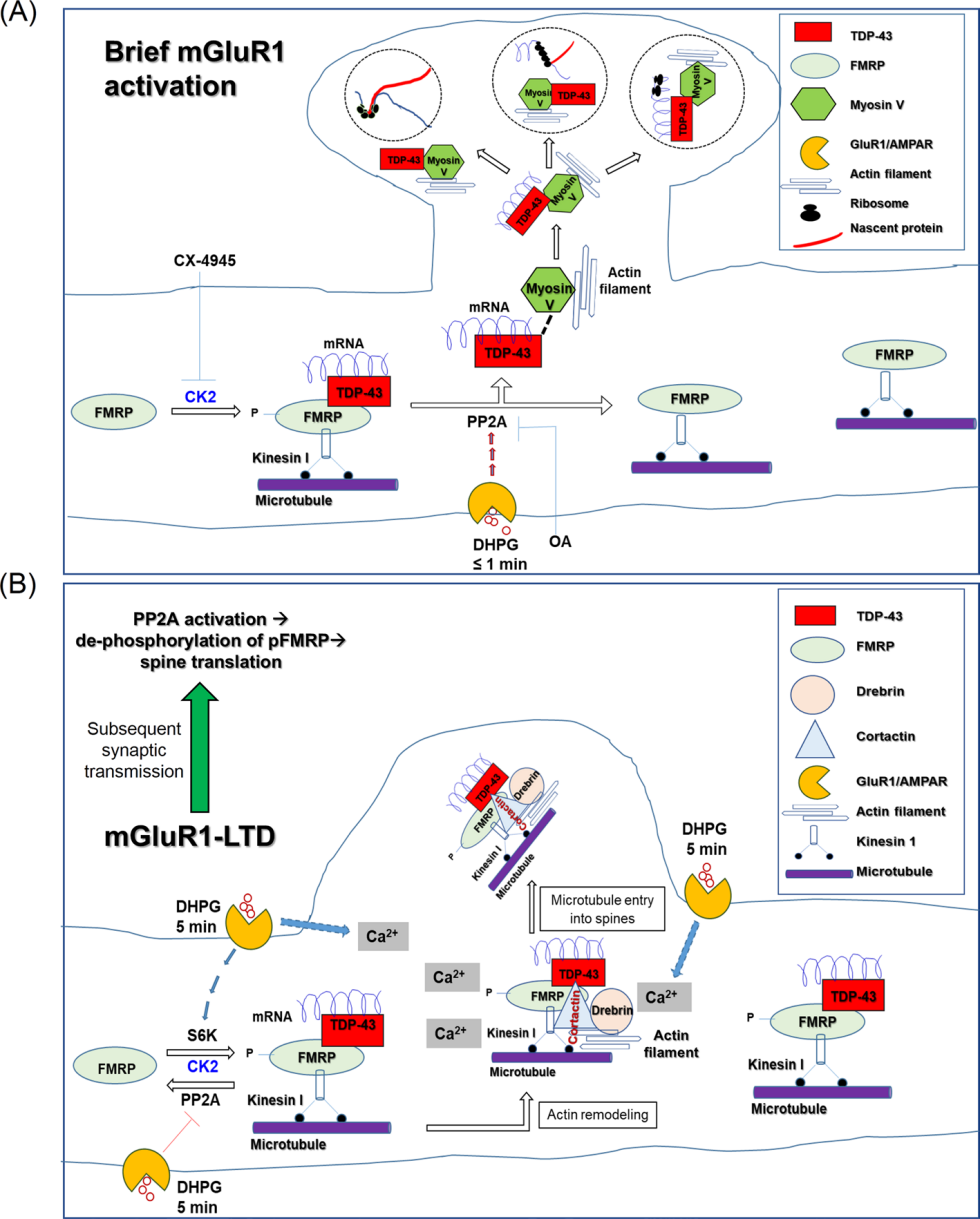
Schematic representation of the dendrite-to-spine transport and translation of TDP-43-bound mRNAs in primary hippocampal neurons as induced by brief mGluR1/5 activation or mGluR-LTD. **A** Illustrated model showing the molecular switches facilitating the dendrite-to-spine transport of TDP-43 and associated mRNA. Phosphorylation of FMRP at S499 is carried out by CK2 in primary hippocampal neuron dendrites [54]. pFMRP exerts a significantly stronger capability than unphosphorylated FMRP to bind TDP-43 (Fig. 3). The complex sits on the microtubule tracks by interacting with kinesin 1, and it is transported mainly in the anterograde direction [43]. The complex remains translationally inactive due to FMRP-driven recruitment of the translational inhibitory complex harboring eIF4E-CYFIP1 (not shown in the illustrated model) [44]. Under immediate stimulation of gp1 mGlu receptors by brief (≤ 1 min) DHPG treatment of the neurons, PP2A is activated, and pFMRP becomes dephosphorylated [[53] (Fig. 2A)], resulting in the dissociation of TDP-43 and bound mRNAs from pFMRP (Fig. 2A, B) and, consequently, from the FMRP-kinesin 1 dendritic transport complex, as well as from the FMRP-associated translation inhibitory complex. TDP-43 and the bound mRNAs then become free to bind with the actin-based motor protein myosin V to enter into the spine (Figs. 1, 2A, C, 4A(a), A(c)). FMRP, on the other hand, continues to move across the dendrites (Fig. 1C and Supplementary Video V3). Therefore, TDP-43 and its bound mRNAs can enter into the spine as a result of the “hand-on transfer” of the mRNP complexes from the FMRP-kinesin 1 dendritic transport complex to the myosin V-associated spine transport complex. This process can be inhibited either by blocking the dephosphorylation of pFMRP by means of the PP2A inhibitor OA (Fig. 2 and Supplementary Video V5) or by blocking the phosphorylation of FMRP using the CK2 inhibitor CX-4945 (Fig. 2 and Supplementary Videos V6 and V7). Inside of the spine, ribosomes associate with these mRNAs, enabling translation of the mRNAs inside of the spine (Fig. 5). Most of the translating mRNA granules inside of the spine are not associated with TDP-43 (Fig. 5B(e)). The average relative distance between TDP-43 or myosin V and the mRNA is also greater within the rest of the translating granules when compared with the untranslating ones (Supplementary Fig. S7), indicating the destabilization of myosin V/TDP-43-mRNA complexes during translation. **B** Illustrated model showing the mechanism of spine transport of TDP-43/mRNA complexes during long DHPG treatment mimicking mGluR-LTD. Prolonged treatment of DHPG inhibits PP2A and activates S6K to phosphorylate FMRP in neurons (see Ref. [73] and Supplementary Fig. S4A). As a result, TDP-43 and bound mRNAs remain associated with FMRP and the dendritic transport complex (Fig. 4A(b), A(d), Supplementary Fig. S4B). Long DHPG treatment also promotes Ca^2+^ entry into the neurons [78], which facilitates association of the actin-binding proteins cortactin and drebrin with kinesin 1, thereby promoting spine entry of the multiple proteins–mRNA complexes (Fig. 4) owing to CYFIP1-assisted and Ca^2+^-dependent actin remodeling followed by microtubule entry into the spine [76, 77, 104]. In contrast to brief mGluR activation, ribosomes cannot associate with mRNAs in the spine of neurons under mGluR-LTD owing to the presence of the FMRP-CYFIP1-eIF4E translational repression complex (data not shown) inhibiting translation in spine (Fig. 5), and resulting in spine shrinkage (Fig. 5A(c)), a hallmark of neuronal LTD [80]. Upon subsequent synaptic transmission, mRNAs inside of the spine become translated owing to dephosphorylation of pFMRP followed by its dissociation from the mRNA

As shown by our illustrated model presented in Fig. 7A, our findings imply a scenario whereby relocation of TDP-43 and its bound mRNAs, such as *Rac1* mRNA, from the dendritic transport complex associated with FMRP-kinesin 1 to the spine transport complex associated with myosin V is orchestrated by dephosphorylation of pFMRP at S499 under the influence of brief DHPG treatment mimicking immediate mGluR1/5 activation. It is noted here that only ~ 20% and ~ 30% of the translating (TL) *Rac1* mRNP granules inside of the spine are associated with TDP-43 under mock and brief DHPG treatment, respectively. In contrast, most (80–90%) of the untranslating (UT) *Rac1* mRNP granules in the spine are associated with TDP-43 (Fig. 5B(e)). In addition, high-resolution SIM microscopy analysis revealed that the distance between translating *Rac1* mRNA and TDP-43 or myosin V is larger than that of the untranslating granules (Supplementary Figs. S7A, B), further indicating that translating *Rac1* mRNA is relatively free of TDP-43 and myosin V protein. Whether dissociation of myosin V/TDP-43 is a prerequisite for translation of *Rac1* mRNA in the spine or if it is a consequence of the translation process remains to be investigated.

Longer treatment with DHPG, on the other hand, is known to elicit mGluR-mediated long-term depression (DHPG-LTD) of neurons. Entry of Ca^2+^ through the transmembrane receptors and release of Ca^2+^from the internal storage of cellular calcium are the hallmarks of gp1 mGluR-mediated synaptic transmission, and have been considered crucial to the prolonged mGluR1/5 activation that results in neuronal LTD [78, 94]. Furthermore, during this latter process, activation of the mTORC1 pathway would lead to deactivation of PP2A and phosphorylation of FMRP by S6 kinase (S6K) [73]. Consistent with the literature, the dendritic pFMRP level under DHPG-LTD condition is similar to that of the mock, but significantly higher than that under brief DHPG treatment (Figs. 4C, Supplementary Figs. S4A, S4B). Interestingly, the association of both FMRP and kinesin 1 with TDP-43/*Rac1* mRNA in the spine increases in long DHPG-treated neurons (Figs. 4A, Supplementary Fig. S4B). In addition, the increase of the associations of TDP-43/*Rac1* mRNA with kinesin 1, cortactin, and drebrin in the spine and synapses of DIV 14 primary hippocampal neurons under the DHPG-LTD condition is Ca^2+^ dependent (Fig. 4B, C). As expected, association of TDP-43 and kinesin1 with cortactin was not altered by CK2 inhibition (data not shown) [73], but decreased upon FMRP depletion (Supplementary Fig. S6D). This confirms the involvement of FMRP in the formation of the cortactin/TDP-43/kinesin1 spine transport complex. Therefore, our findings provide evidence for a mechanism of dendrite-to-spine transport of TDP-43-associated mRNAs in neurons under the DHPG-LTD condition, as illustrated in Fig. 7B. We propose that Ca^2+^-mediated actin remodeling at the dendritic shaft leads to microtubule invasion into the spine, resulting in “direct spine entry” of the microtubule motor protein kinesin 1, RBPs, e.g., TDP-43 and FMRP, and TDP-43-associated mRNAs, such as *Rac1* mRNA.

However, this spine transport mechanism does not operate under the mock condition during restricted neuronal Ca^2+^ entry. In absence of TDP-43/FMRP association, mRNA transport complex consisting of cortactin/TDP-43/kinesin1 cannot be formed (Supplementary Figs. S4C and S6D) even with increased Ca^2+^ entry under brief DHPG treatment. Furthermore, our data indicate that, during long DHPG treatment, mature spine formation is impaired because the TDP-43-bound *Rac1* mRNA remains translationally silent in the spine, likely owing to FMRP-mediated recruitment of a translation repression complex (Fig. 5). This immature spine formation eventually elicits structural LTD. Notably, a previous study has shown that the spine transport and translation of mRNAs, e.g., *PSD-95* mRNA and *CamKII* mRNA, appear to be uncoupled under mGluR-LTD [62]. Although the translation status of different mRNAs transported to the spine under mGluR-LTD may vary, accumulation of some of them, such as *Rac1* mRNA, inside of the spine likely facilitates the rapid attainment of neuronal potentiation during subsequent synaptic transmission without undergoing the dendrite-to-spine transport process.

During LTD, altered phosphorylation status of FMRP also regulates the stress granule (SG) dynamics [95]. Importantly, both TDP-43 and FMRP can modulate the structure/function and components of SG [96]. Specifically, FMRP represses the translation of its target mRNAs [95] and presumably target mRNAs of TDP-43 as well, under stress condition. To understand the co-operative regulation of SG dynamics under mGluR stimulation, further investigation will be needed. Recently it has been established that TDP-43 and FMRP play important roles in pre-synapses and axons [45, 46]. Undoubtedly, this is an important piece of information, but it will not change the interpretations of our results as we carefully chose dendrites to study and often reconfirmed by Map2/Tau [44] or PSD-95 immunostaining [43], used high-resolution microscopy, analyzed granules containing dendritic spine proteins, e.g., cortactin, drebrin, etc., and studied effects of DHPG treatment, the receptor of which is absent in axons.

With respect to the results in Fig. 6, it is interesting to note that a subpopulation of autism patients and several animal models of ASD have been reported to exhibit altered LTP and mGluR-LTD [21, 87]. For example, patients with ASD with Shank3 mutations had disrupted grp1 mGluR function [84]. Moreover, local translation processes are severely impaired in the brains of almost all patients with ASD and mouse models [87]. Since dendritic spine translation is controlled by FMRP and other translation regulatory proteins, the translational abnormalities of ASD disease have been examined in FMRP-deficient Fmr1 KO mice [31] and a Tg eIF4E mouse model [97]. However, whether and how dendrite-to-spine transport of neuronal mRNAs for translation in the spine under different neurotransmissions is hampered was previously unknown.

Using the maternally VPA-injected mouse model of ASD, we demonstrate the misregulation in the molecular switch of the dendrite-to-spine transport of TDP-43-associated mRNAs for translation under immediate mGluR activation and mGluR-mediated LTD conditions, respectively, in ASD neurons (Fig. 6). As observed before [32, 98] by others, the levels of synaptosomal mRNAs, e.g., PSD-95, Syn1, Shank3 (Fig. 6C), proteins, e.g., Rac1 and GluR1 (data not shown), altered in synaptosome extracts from primary hippocampal neuron derived from the VPA-treated embryos. The structures of the spine of these neurons are also abnormal (Fig. 6D), as previously reported [99]. Our analysis of the brain tissue sections from the ASD mouse model further indicates impairment of the association between TDP-43 and FMRP in vivo owing to the decreased phosphorylation of FMRP at S499 (Fig. 6B). Notably, decreased pFMRP level has also been reported in different parts of the brains of patients with ASD [32]. Further analysis of the primary hippocampal neuron culture from maternally VPA-treated embryos has shown that, irrespective of the neurotransmission signals, TDP-43 and associated *Rac1* mRNA are free to associate with myosin V [Figs. 6D(a), D(b)], transported into nearby spine-like structures, and become translated, resulting in an increased number of long immature spine formation (Figs. 6D(c), Supplementary Fig. S9D). In contrast, the primary neuron culture from saline-injected control exhibits changes of the percentage of TDP-43-bound mRNA puncta associated with myosin V, the spine structure, and the spine density under different treatment conditions (Fig. 6D(a), D(b)). Interestingly, overexpression of phosphomimetic mutant [GFP-FMRP (S499D)] in primary neuron culture from VPA-ASD mouse model can decrease spine transport of *Rac1* mRNA and consequently decreases immature thin spine density (Supplementary Figs. S8C and S9D). Therefore, one major contribution to the pathogenesis of a subpopulation of ASD could be the misregulation of neurotransmission-induced dendrite-to-spine transport of TDP-43-associated neuronal mRNAs as the consequence of a reduced level pFMRP (S499). Lower FMRP phosphorylation in VPA-ASD mouse model presumably also results in the alteration of dynamics of some TDP-43-associated SG dynamics that likely further contributes to the disease pathogenicity.

The link between TDP-43 and ASD identified in this study further suggests that mutations and/or malfunction of TDP-43 could play a significant role in the pathogenesis of neurodevelopmental disorders such as ASD. Recently, many patients with ASD have been reported to exhibit neuron loss in different areas of the brain, e.g., cerebellum, fusiform gyrus, and parts of brain with pyramidal neurons, along with other neurodegenerative disease-like symptoms [100, 101]. As observed in different neurodegenerative diseases such as AD and PD [102], abnormalities in grp1 mGluR-dependent signaling are also detected among patients with ASD and ASD mouse models [103]. Our data described above provide a molecular connection between ASD and TDP-43, thus opening up a new perspective for future research to elucidate the neurodegeneration and TDP-43 proteinopathy among patients with ASD.

## Conclusions

Overall, this study has uncovered the molecular switches and the underlying mechanisms facilitating the translocation of TDP-43-associated dendritic mRNA, i.e., *Rac1* mRNA, cargos into the dendritic spine of neurons under grp1 mGluR-mediated different synaptic transmissions. Importantly, the difference in the phosphorylation status of FMRP at S499, resulting in its differential association with TDP-43-bound mRNAs under brief mGluR activation and under mGluR-LTD, as well as the local Ca^2+^ concentration-mediated microtubule entry into the spine under mGluR-LTD, appear to be the major factors determining the distinctive mechanisms of dendrite-to-spine entry and the fates of translation of TDP-43-bound mRNAs inside the spine. Significantly, the analysis of the VPA-induced mouse model of ASD, in combination with the previous finding by others of the low level of pFMRP (S499) in the different brain regions of patients with ASD, suggest that this molecular switch appears to be nonfunctional in patients with ASD with reduced pFMRP level, as the result of dissociation of pFMRP from the TDP-43/mRNA complexes, leading to the loss of control of dendrite-to-spine transport and translation of these mRNAs, immature spine formation, as well as nonresponsiveness toward grp1 mGluR-mediated neurotransmissions.

## Supplementary Information

Supplementary Material 1: Raw images of WB gel pictures shown in Fig. 2A(i), left.

Supplementary Material 2: Raw images of WB gel pictures shown in Fig. 2A(i), right.

Supplementary Material 3: Raw images of WB gel pictures shown in Fig. 2A(ii), left.

Supplementary Material 4: Raw images of WB gel pictures shown in Fig. 2A(ii), right.

Supplementary Material 5: Raw images of WB gel pictures shown in Fig. 3A(a).

Supplementary Material 6: Raw images of WB gel pictures shown in Fig. 4C(a).

Supplementary Material 7: Raw images of WB gel pictures shown in Fig. 4C(a), input.

Supplementary Material 8: Raw images of WB gel pictures shown in Supplementary Fig. S2A(b), B.

Supplementary Material 9: Raw images of WB gel pictures shown in Supplementary Fig. S4A.

Supplementary Material 10: Raw images of WB gel pictures shown in Supplementary Fig. S6D.

Supplementary Material 11: Raw images of WB gel pictures shown in Supplementary Fig. S8A.

Supplementary Material 12: Raw images of WB gel pictures shown in Supplementary Fig. S8A, input.

Supplementary Material 13: Raw images of WB gel pictures shown in Supplementary Fig. S8B.

Supplementary Material 14.

Supplementary Material 15.

Supplementary Material 16.

Supplementary Material 17.

Supplementary Material 18.

Supplementary Material 19.

Supplementary Material 20.

Supplementary Material 21.

Supplementary Material 22.

Supplementary Material 23.

Supplementary Material 24.

Supplementary Material 25.

Supplementary Material 26.

Supplementary Material 27.

Supplementary Material 28.

Supplementary Material 29.

Supplementary Material 30.

Supplementary Material 31.

Supplementary Material 32.

Supplementary Material 33.

Supplementary Material 34.

Supplementary Material 35.

## Data Availability

The corresponding authors are declaring that all data used in this manuscript will be available on request. Raw images of 55 different gel/WB pictures are shown in Additional files as part of Supplementary Material.

## Abbreviations

LTD: Long-term depression
ASD: Autism spectrum disorder
TDP-43: Tar DNA binding protein 43
FMRP: Fragile X mental retardation protein
RBP: RNA binding protein
mGluR: Metabotropic glutamate receptors
gp1 mGluRs: Group 1 mGluRs
LTP: Long-term potentiation
STP: Short-term potentiation
AMPAR: α-Amino-3-hydroxy-5-methyl-4-isoxazole-propionic acid receptor
NMDAR: *N*-methyl-d-aspartic acid receptors
mRNPs: Messenger ribonucleoproteins
TLS: Translocated in liposarcoma
AD: Alzheimer’s disease
ALS: Amyotrophic lateral sclerosis
FTD: Frontotemporal dementia
VPA: Valproic acid
DHPG: 3,5-Dihydroxyphenylglycine
TRICK: Translating RNA imaging by coat protein knockoff
S6K: S6 kinase
