# Supplementary figures and images for: Molecular switch of the dendrite-to-spine transport of TDP-43/FMRP-bound neuronal mRNAs and its impairment in ASD
Source: Cell Mol Biol Lett. 2025 Jan 15;30:6. doi: 10.1186/s11658-024-00684-5 (PMC11737055; doi:10.1186/s11658-024-00684-5)

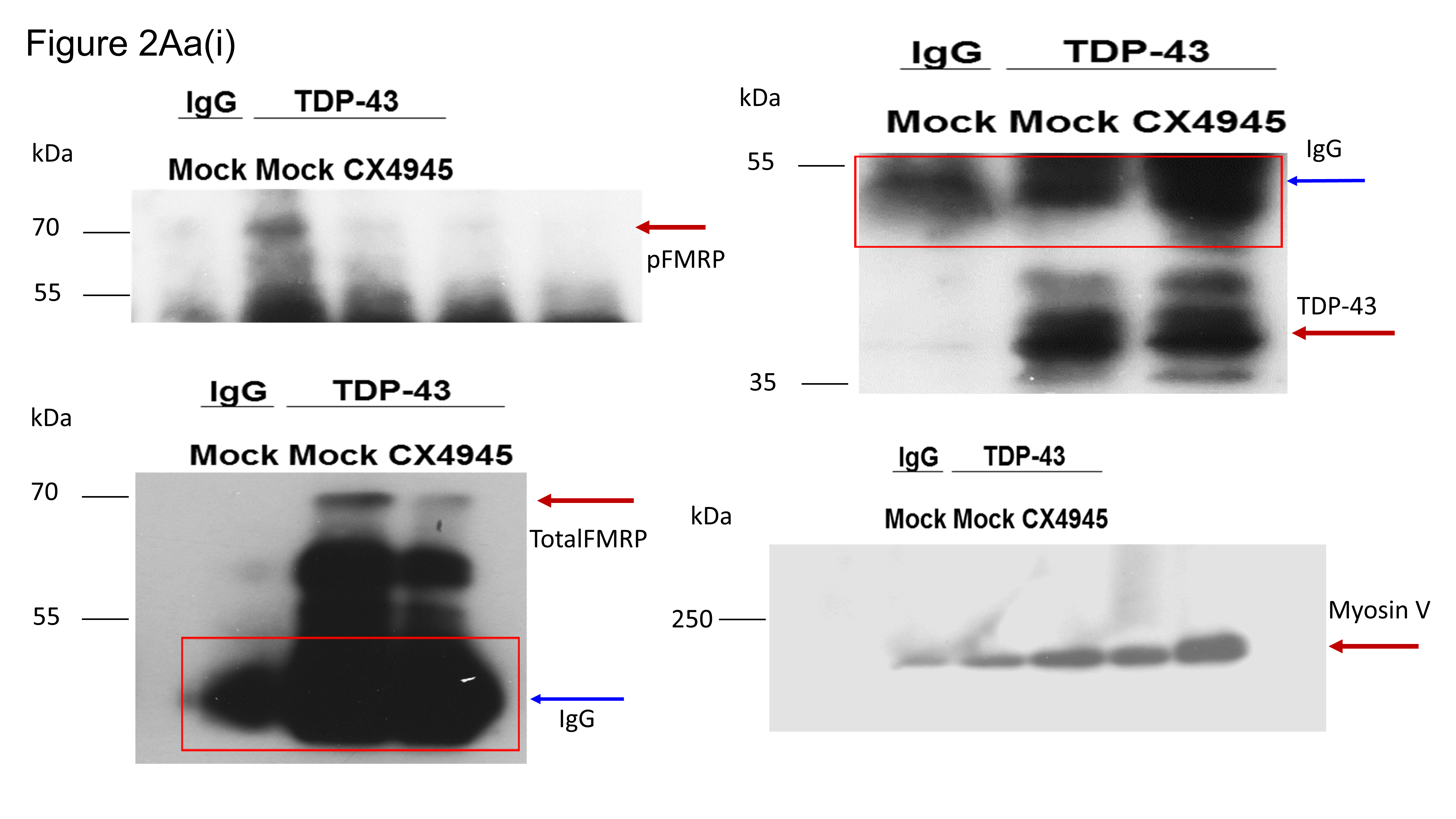

Supplement: Supplementary file 1 — Supplementary Material 1: Raw images of WB gel pictures shown in Fig. 2A(i), left. [file 11658_2024_684_MOESM1_ESM.tif]

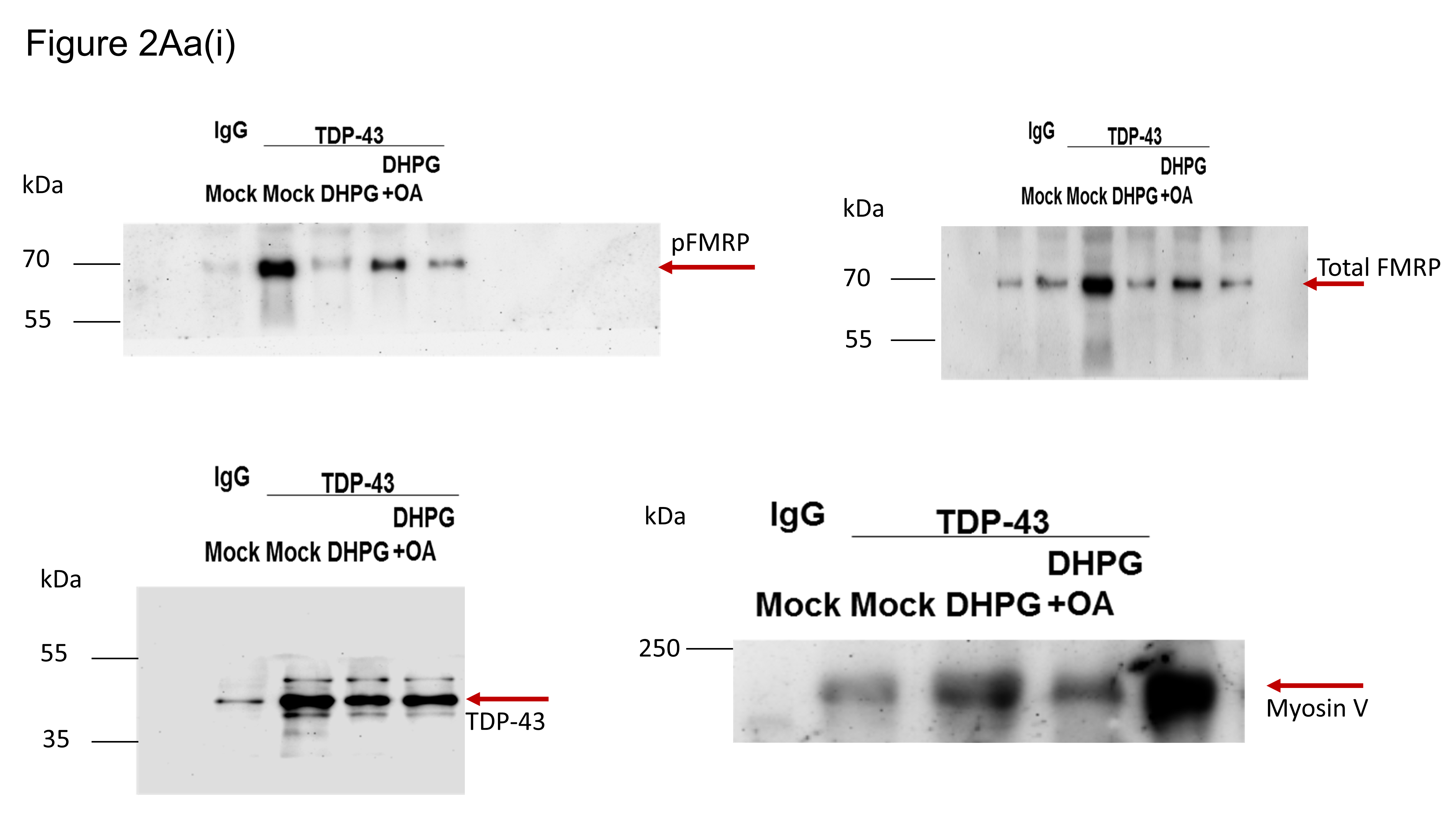

Supplement: Supplementary file 2 — Supplementary Material 2: Raw images of WB gel pictures shown in Fig. 2A(i), right. [file 11658_2024_684_MOESM2_ESM.tif]

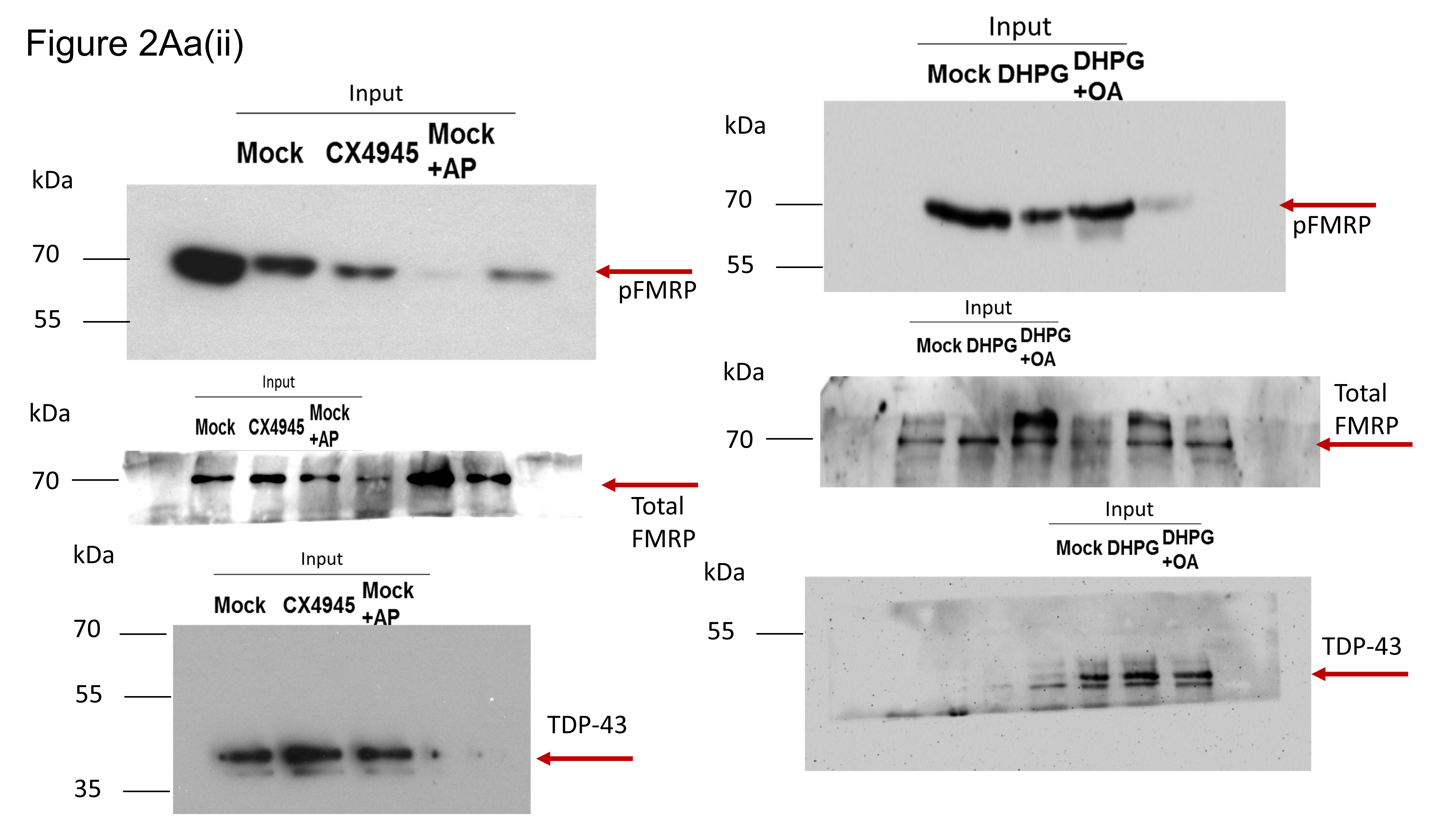

Supplement: Supplementary file 3 — Supplementary Material 3: Raw images of WB gel pictures shown in Fig. 2A(ii), left. [file 11658_2024_684_MOESM3_ESM.tif]

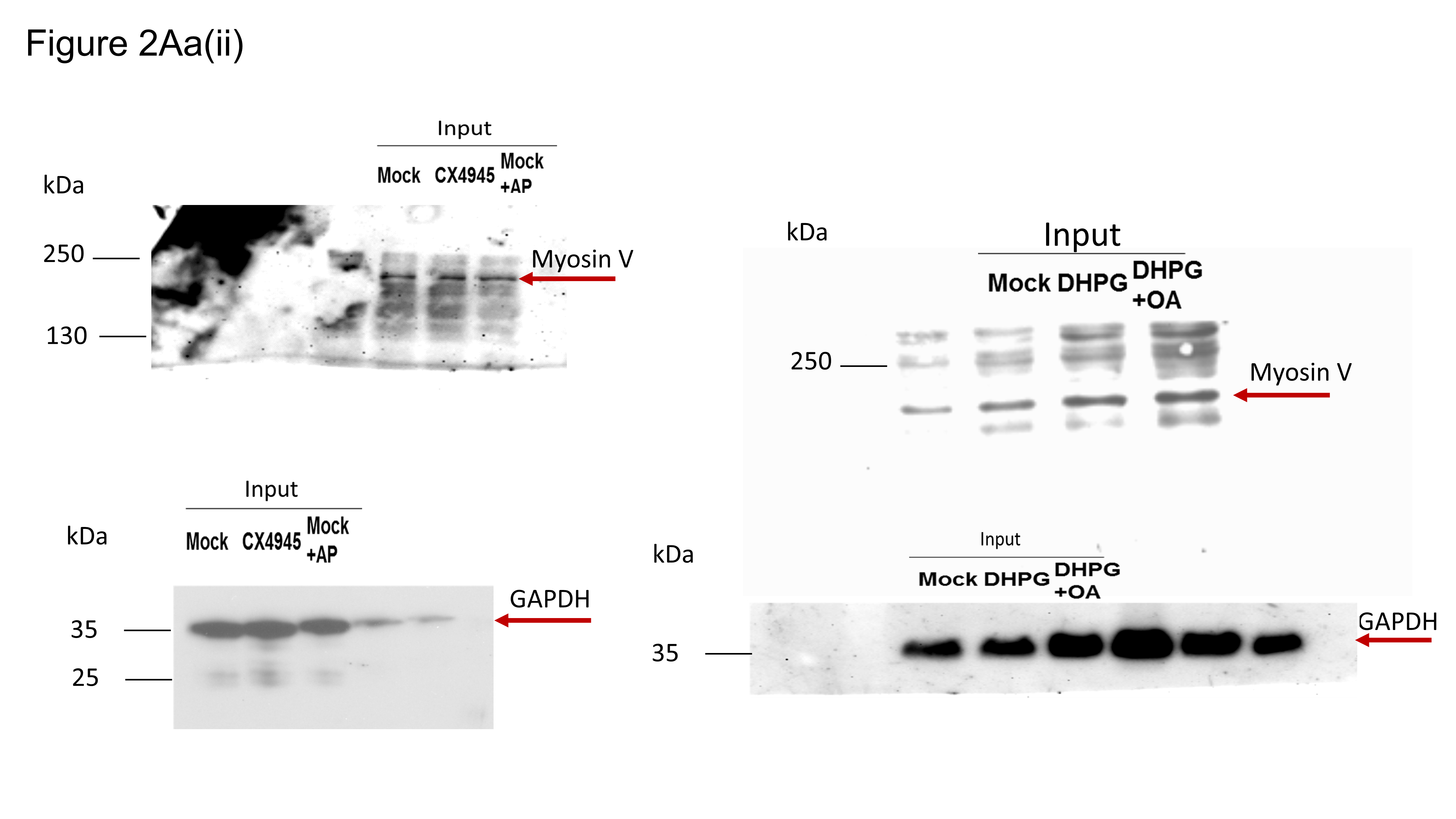

Supplement: Supplementary file 4 — Supplementary Material 4: Raw images of WB gel pictures shown in Fig. 2A(ii), right. [file 11658_2024_684_MOESM4_ESM.tif]

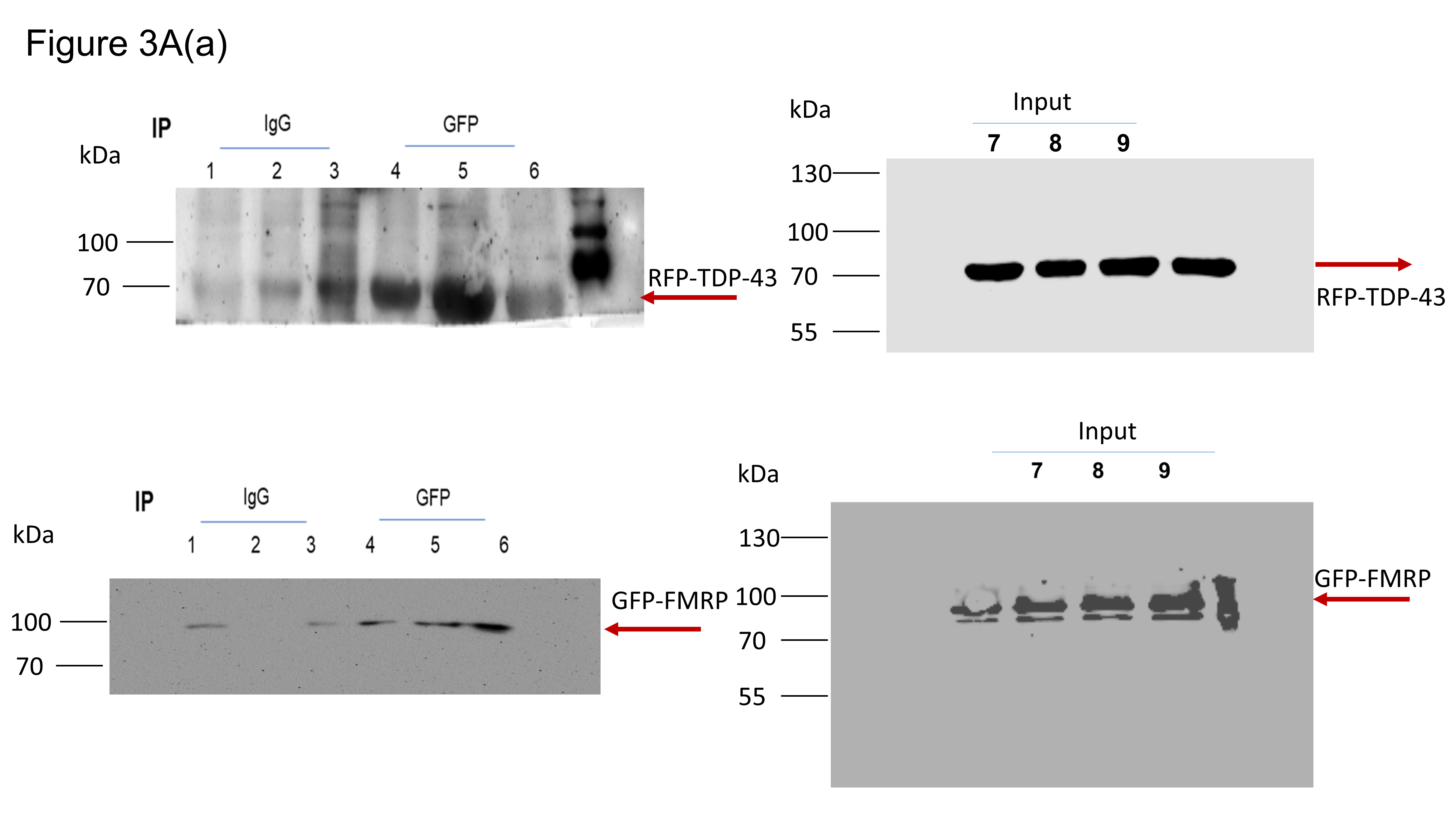

Supplement: Supplementary file 5 — Supplementary Material 5: Raw images of WB gel pictures shown in Fig. 3A(a). [file 11658_2024_684_MOESM5_ESM.tif]

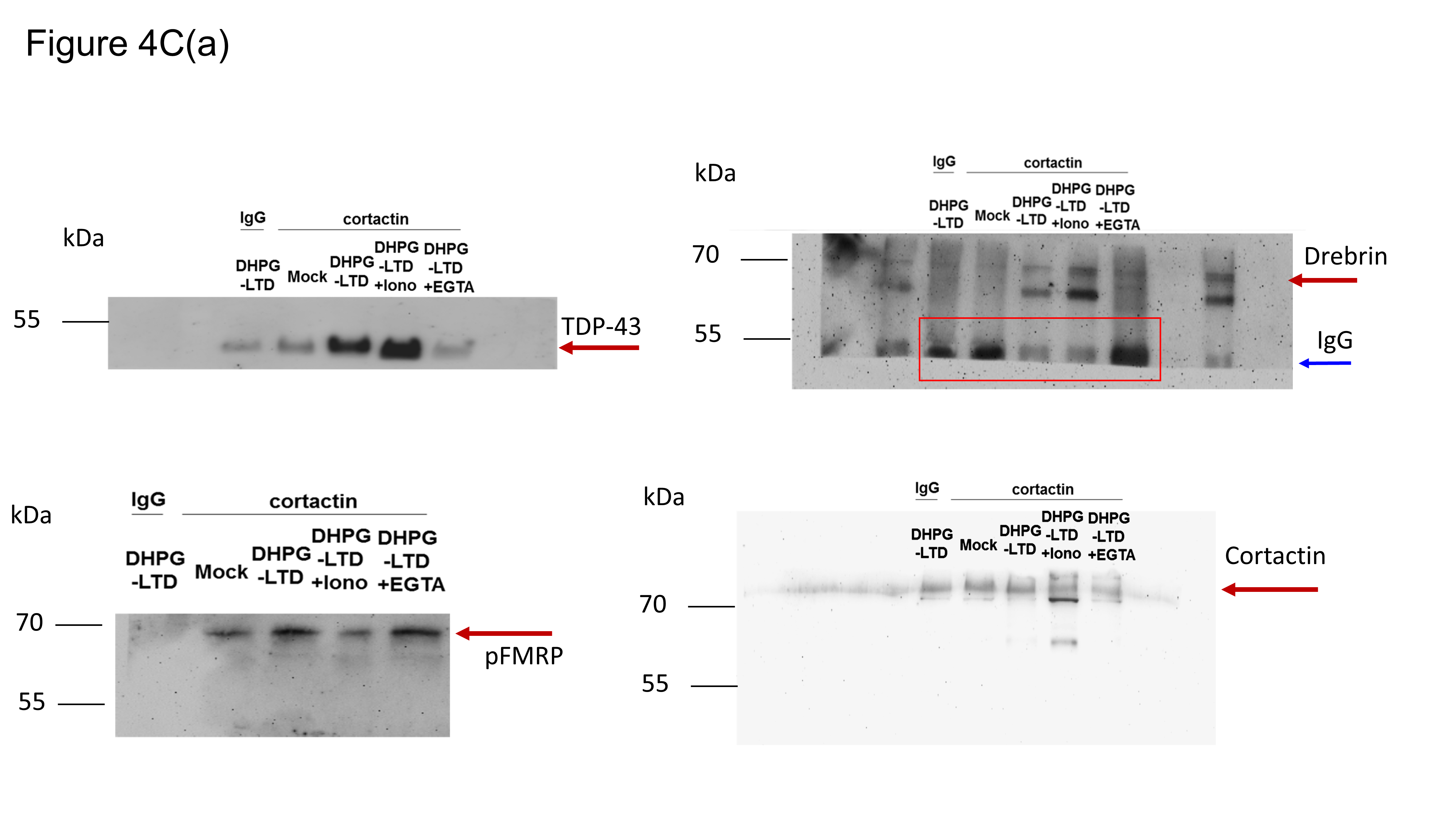

Supplement: Supplementary file 6 — Supplementary Material 6: Raw images of WB gel pictures shown in Fig. 4C(a). [file 11658_2024_684_MOESM6_ESM.tif]

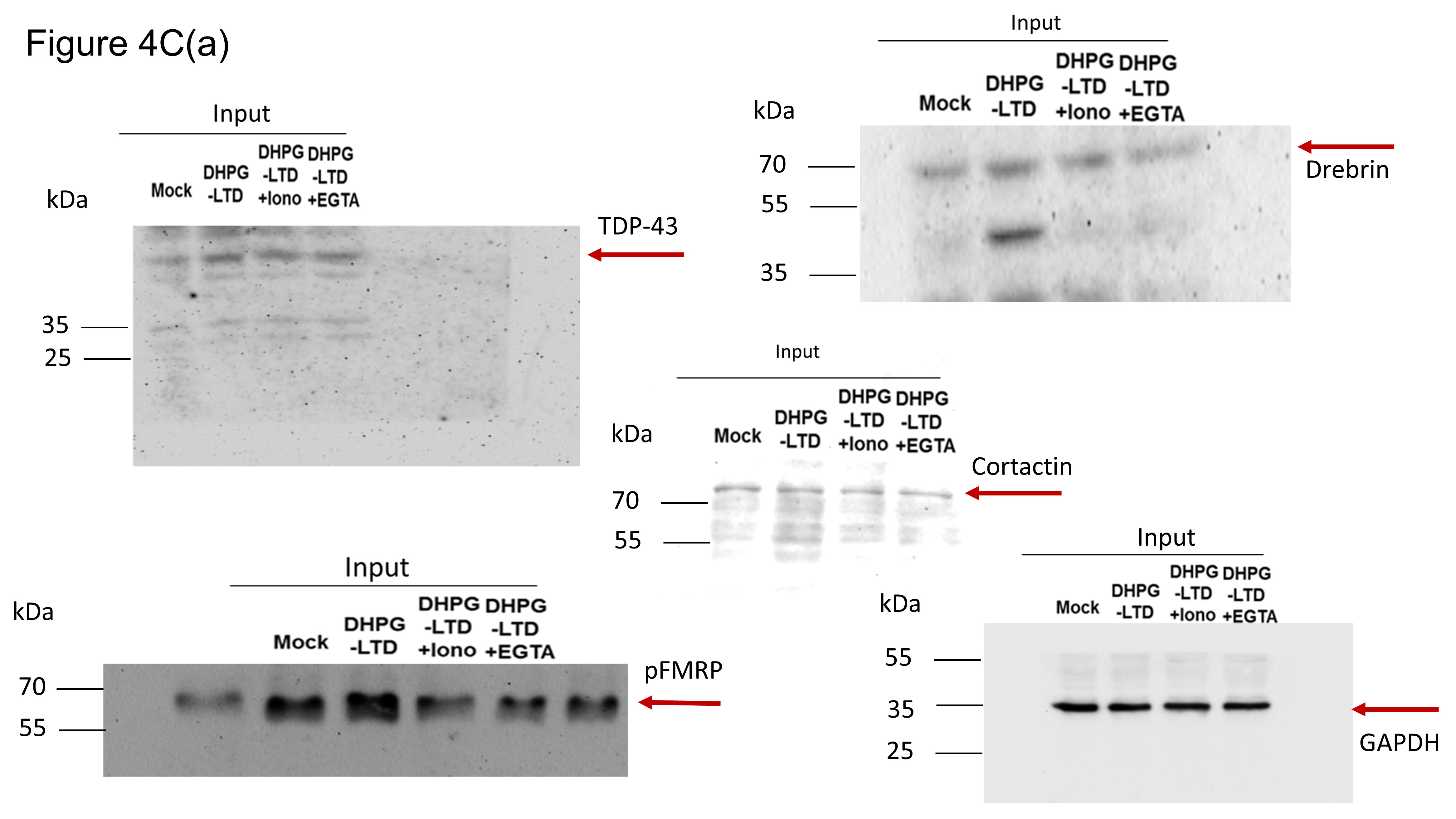

Supplement: Supplementary file 7 — Supplementary Material 7: Raw images of WB gel pictures shown in Fig. 4C(a), input. [file 11658_2024_684_MOESM7_ESM.tif]

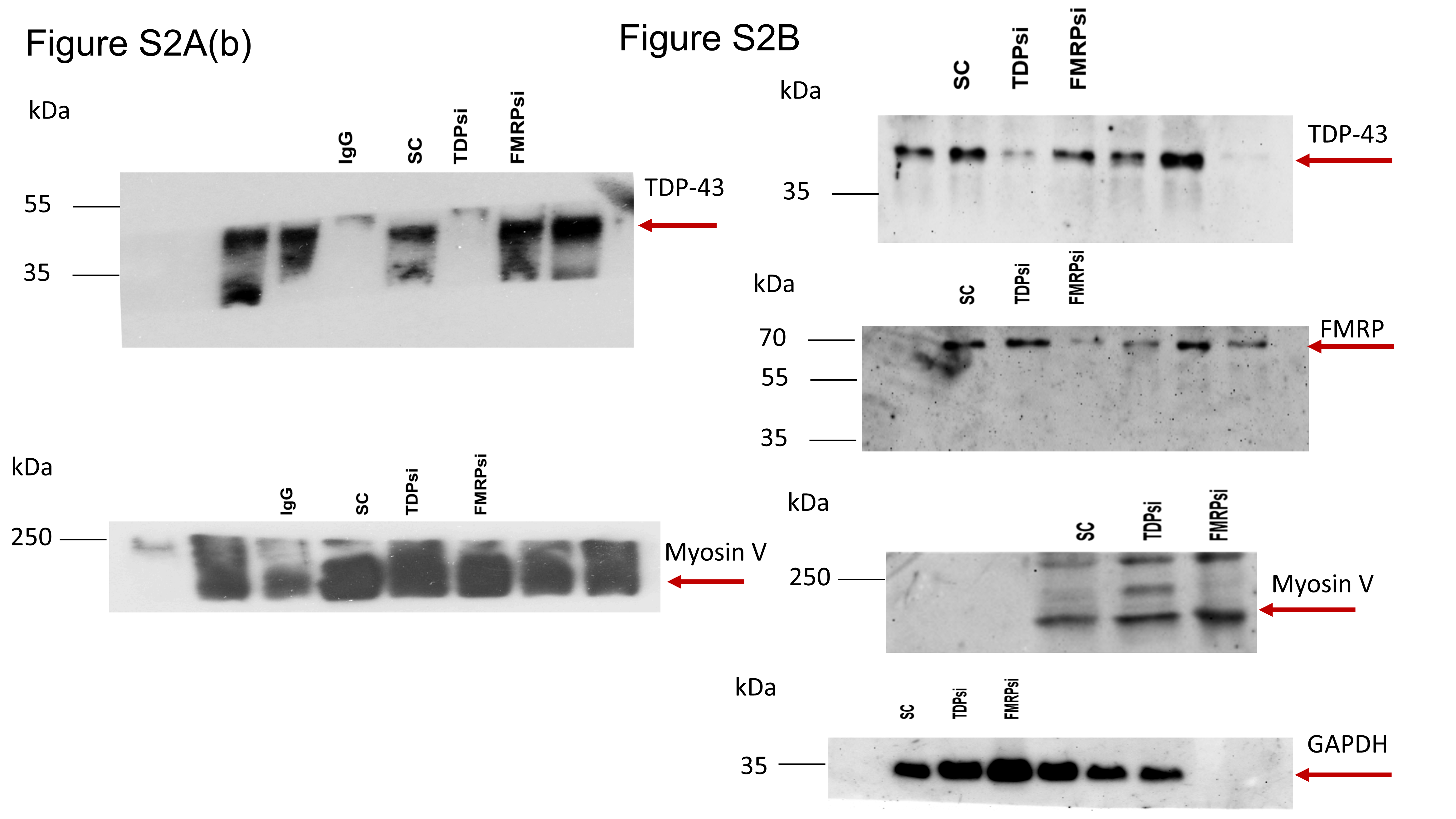

Supplement: Supplementary file 8 — Supplementary Material 8: Raw images of WB gel pictures shown in Supplementary Fig. S2A(b), B. [file 11658_2024_684_MOESM8_ESM.tif]

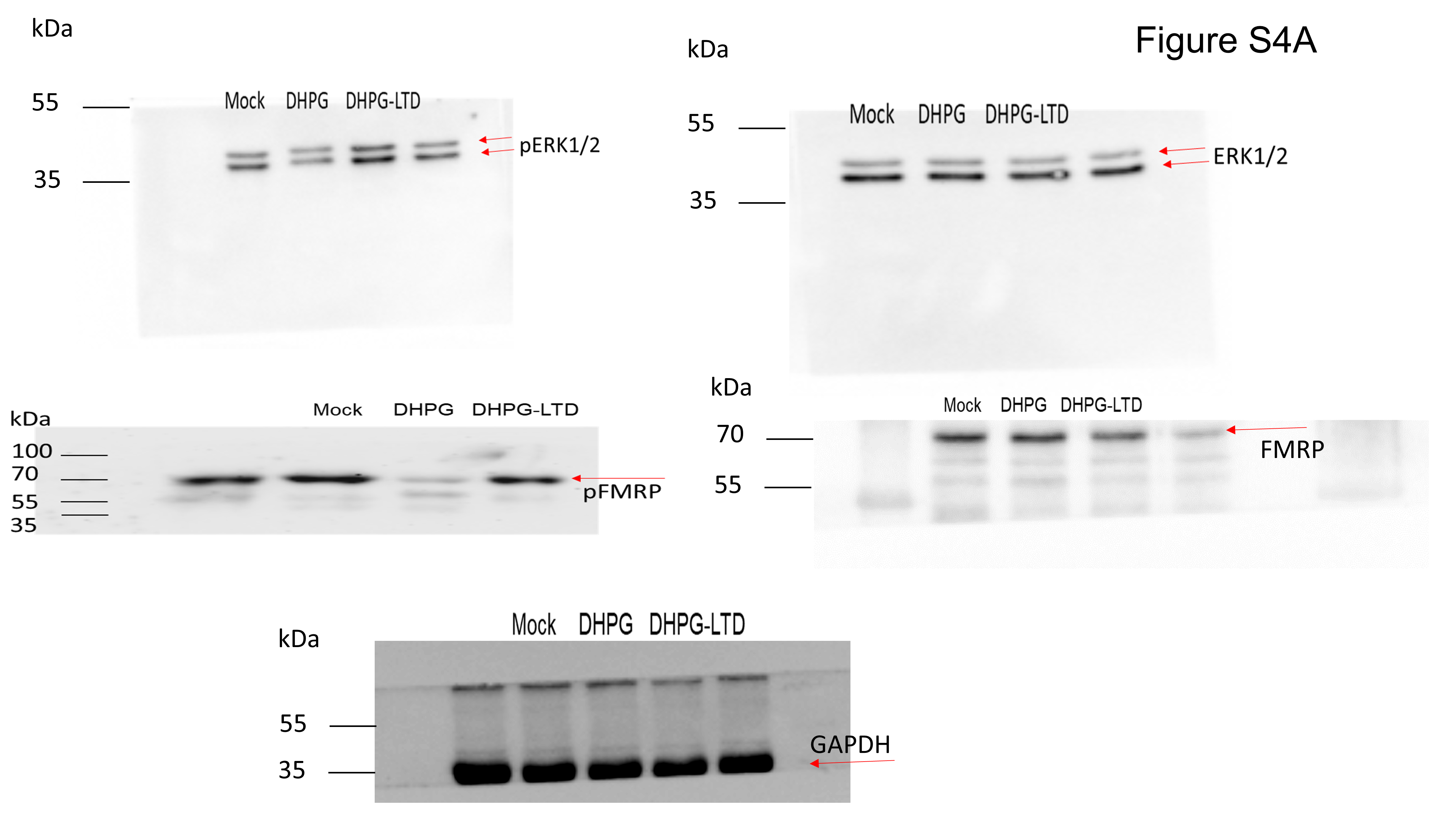

Supplement: Supplementary file 9 — Supplementary Material 9: Raw images of WB gel pictures shown in Supplementary Fig. S4A. [file 11658_2024_684_MOESM9_ESM.tif]

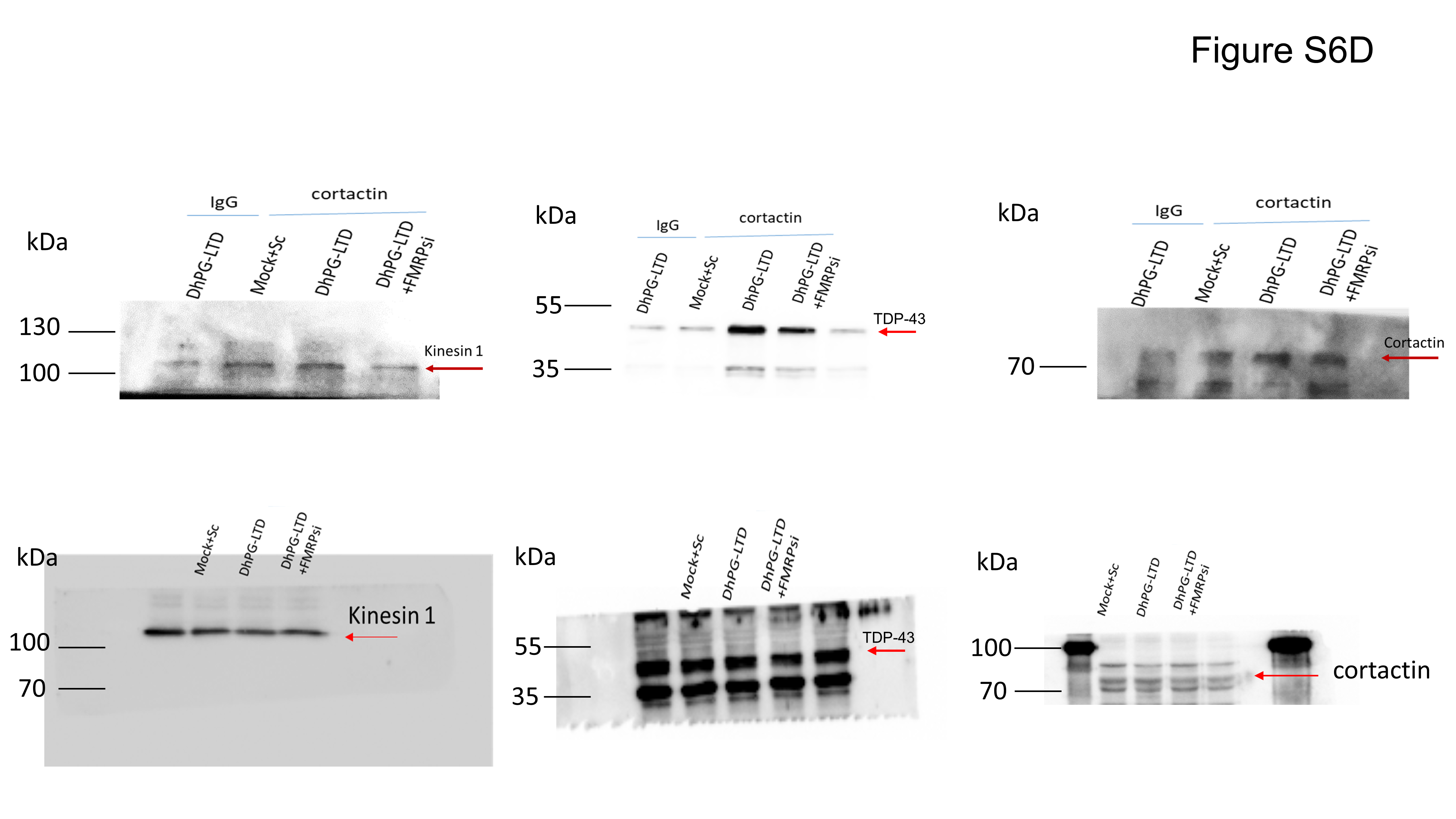

Supplement: Supplementary file 10 — Supplementary Material 10: Raw images of WB gel pictures shown in Supplementary Fig. S6D. [file 11658_2024_684_MOESM10_ESM.tif]

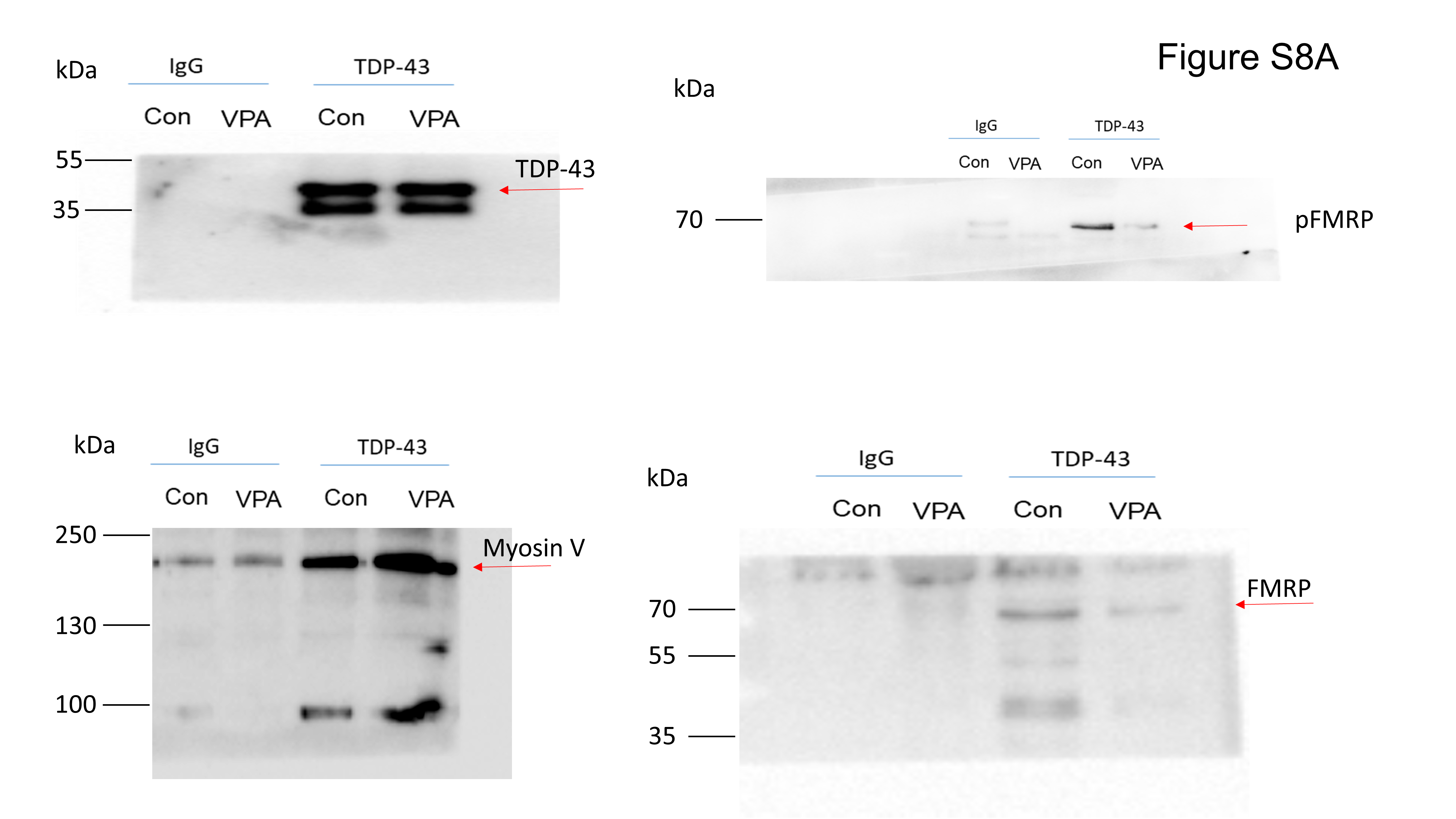

Supplement: Supplementary file 11 — Supplementary Material 11: Raw images of WB gel pictures shown in Supplementary Fig. S8A. [file 11658_2024_684_MOESM11_ESM.tif]

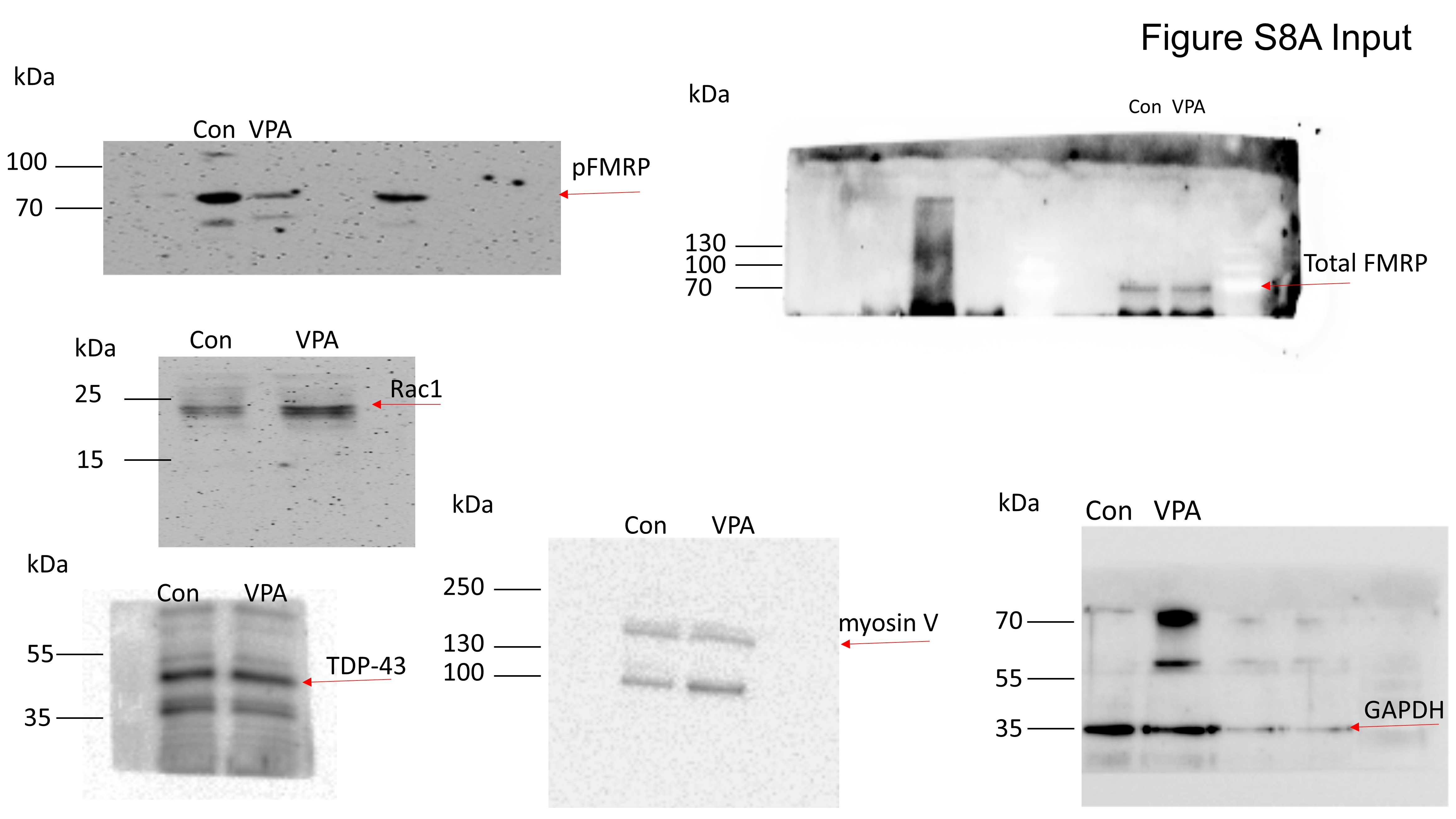

Supplement: Supplementary file 12 — Supplementary Material 12: Raw images of WB gel pictures shown in Supplementary Fig. S8A, input. [file 11658_2024_684_MOESM12_ESM.tif]

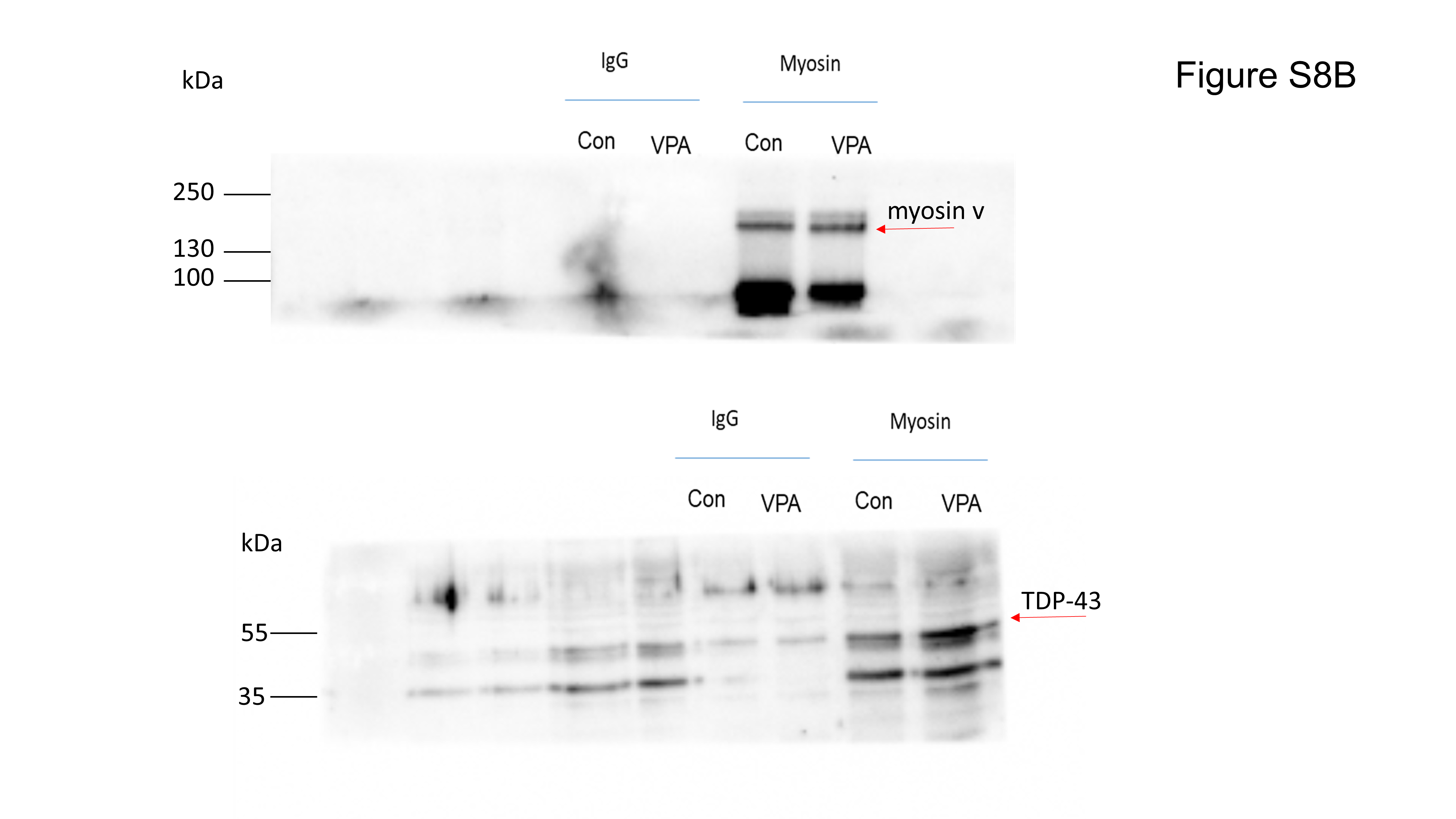

Supplement: Supplementary file 13 — Supplementary Material 13: Raw images of WB gel pictures shown in Supplementary Fig. S8B. [file 11658_2024_684_MOESM13_ESM.tif]

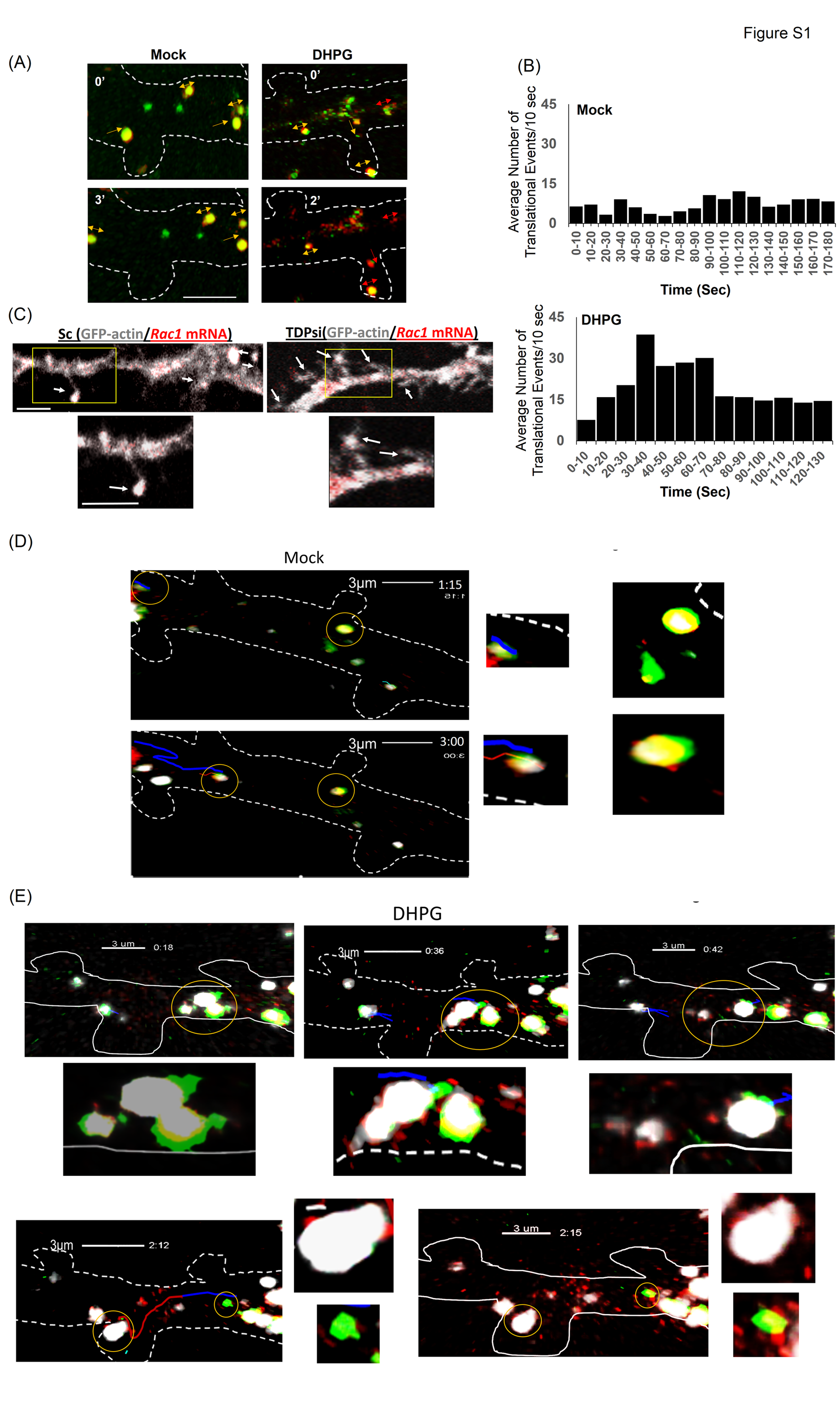

Supplement: Supplementary file 15 — Supplementary Material 15. [file 11658_2024_684_MOESM15_ESM.tif]

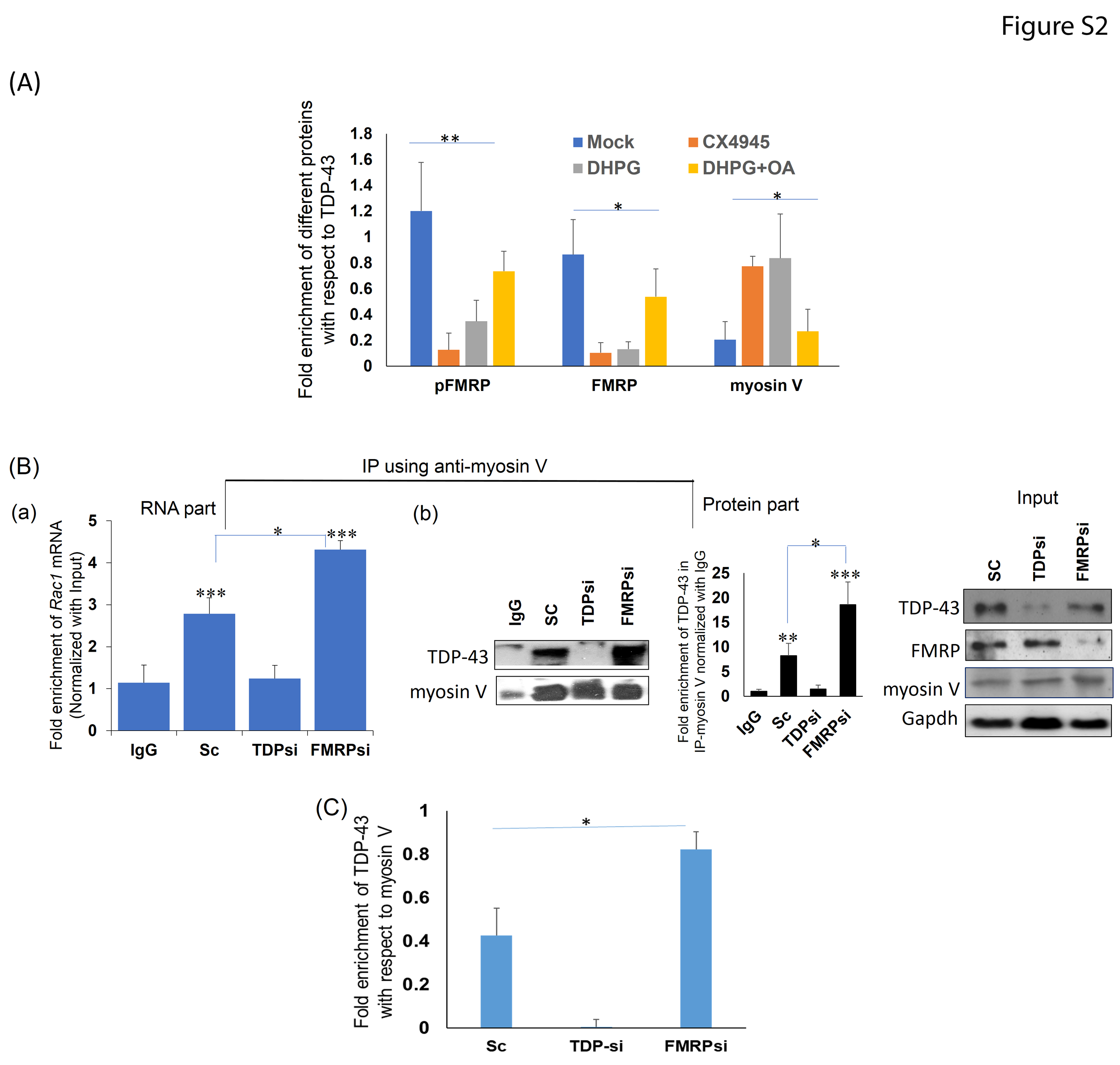

Supplement: Supplementary file 16 — Supplementary Material 16. [file 11658_2024_684_MOESM16_ESM.tif]

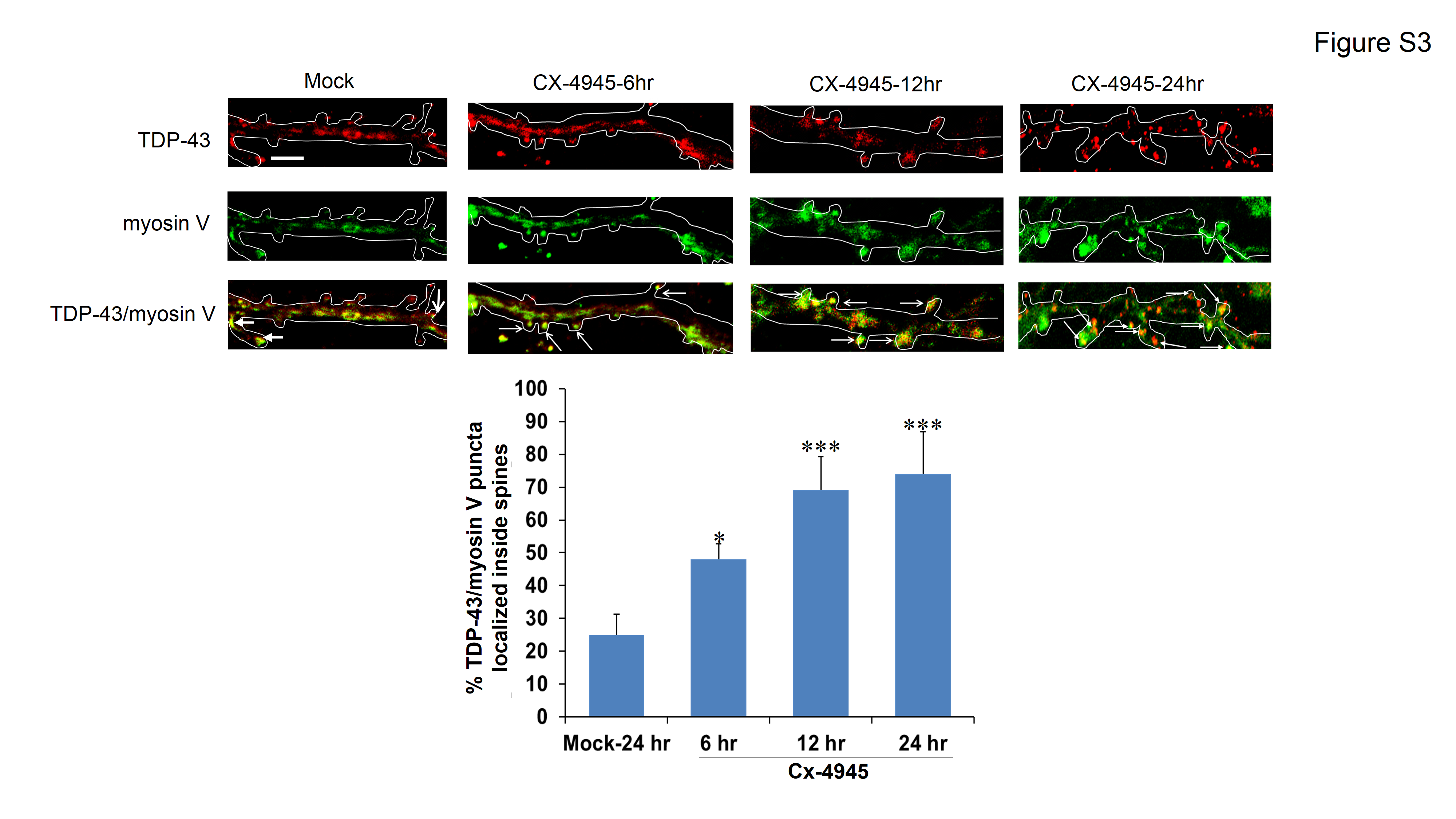

Supplement: Supplementary file 17 — Supplementary Material 17. [file 11658_2024_684_MOESM17_ESM.tif]

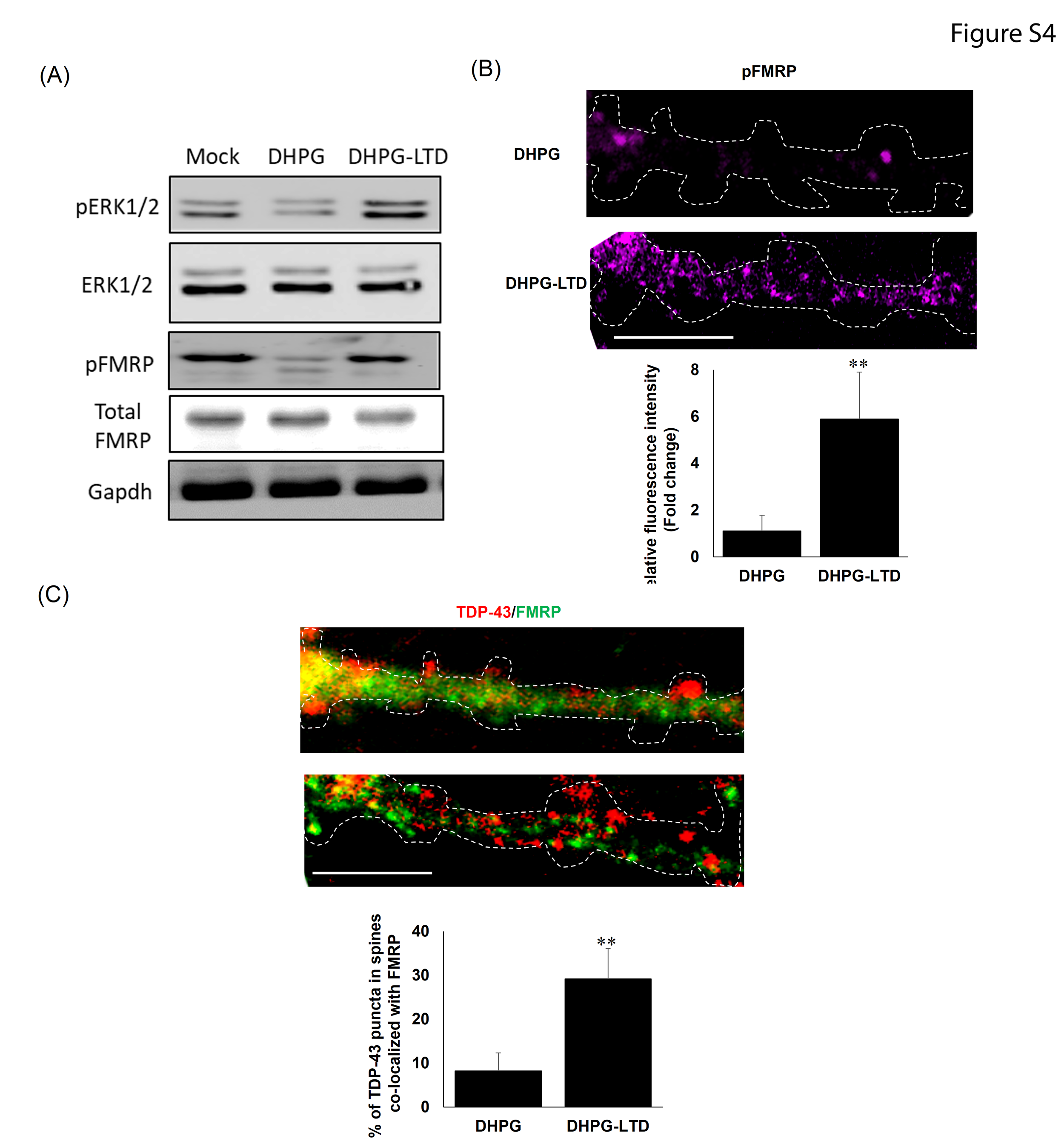

Supplement: Supplementary file 18 — Supplementary Material 18. [file 11658_2024_684_MOESM18_ESM.tif]

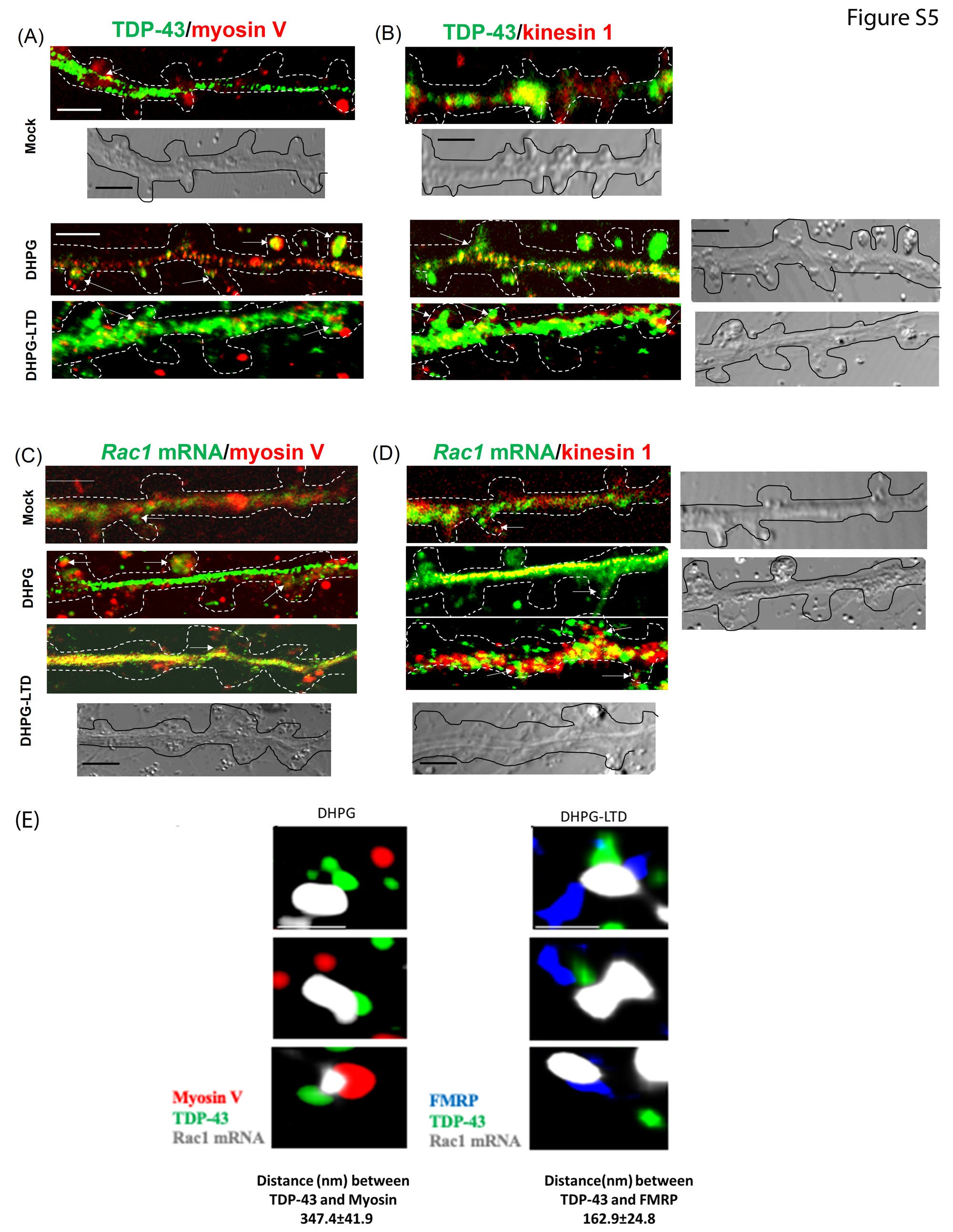

Supplement: Supplementary file 19 — Supplementary Material 19. [file 11658_2024_684_MOESM19_ESM.tif]

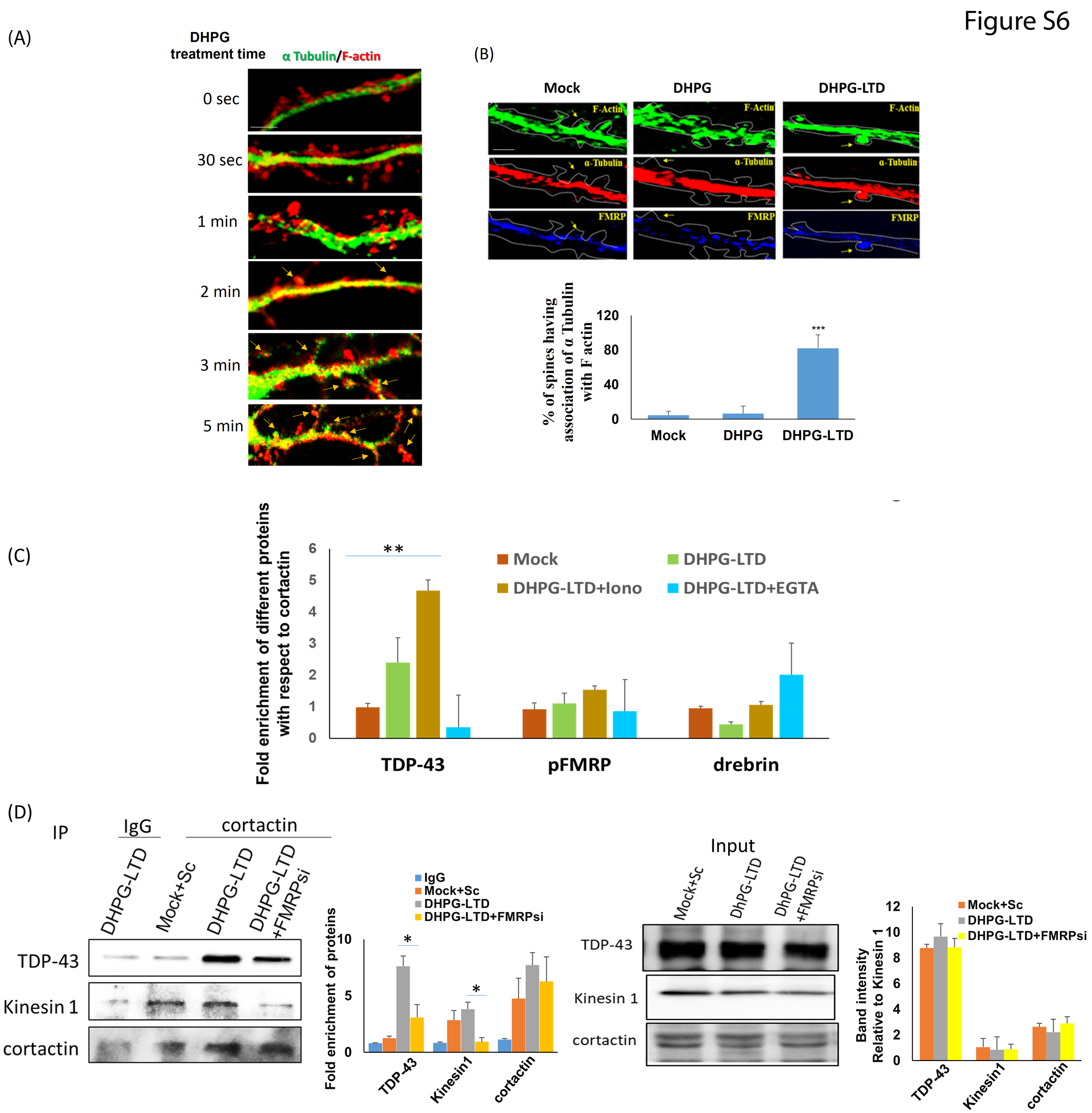

Supplement: Supplementary file 20 — Supplementary Material 20. [file 11658_2024_684_MOESM20_ESM.tif]

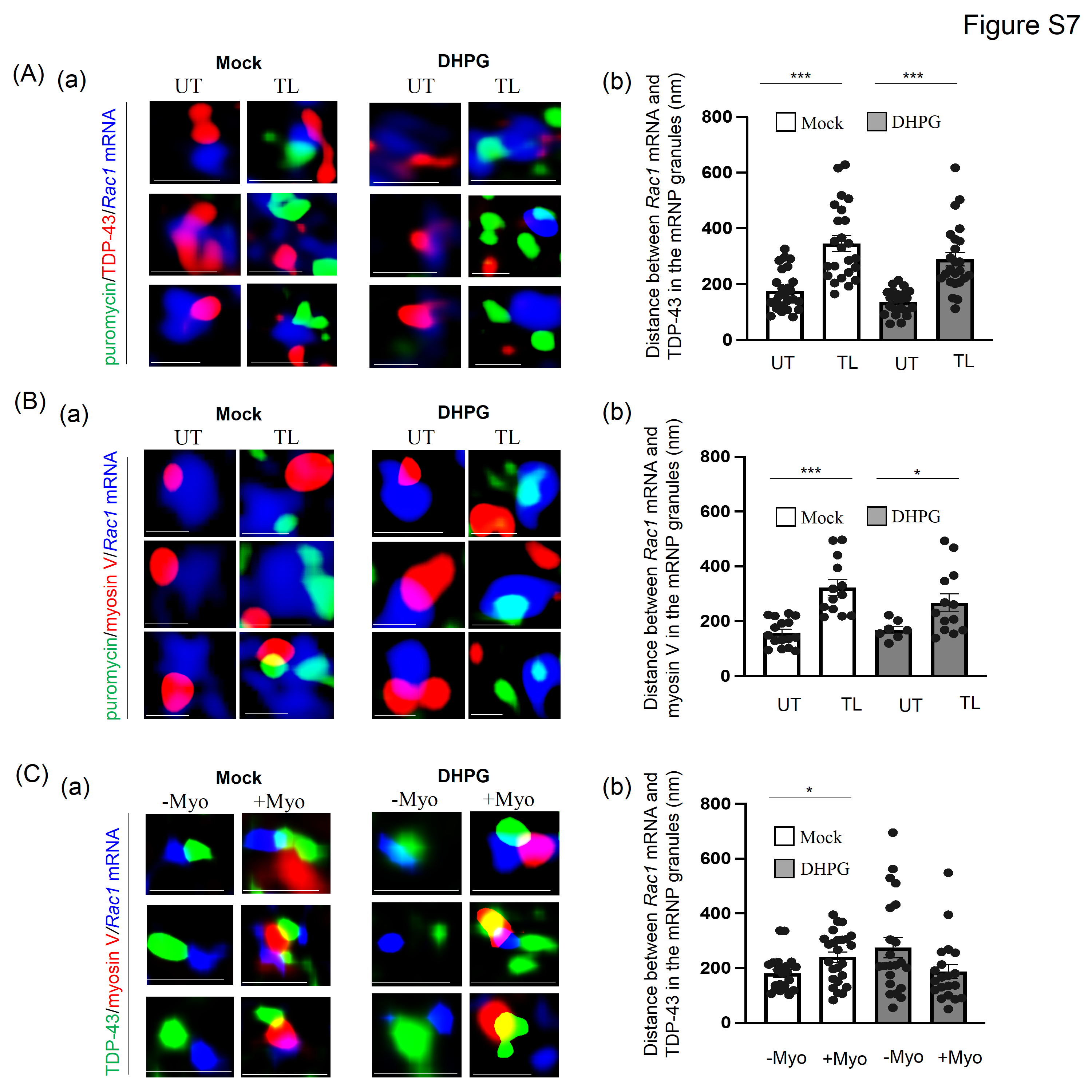

Supplement: Supplementary file 21 — Supplementary Material 21. [file 11658_2024_684_MOESM21_ESM.tif]

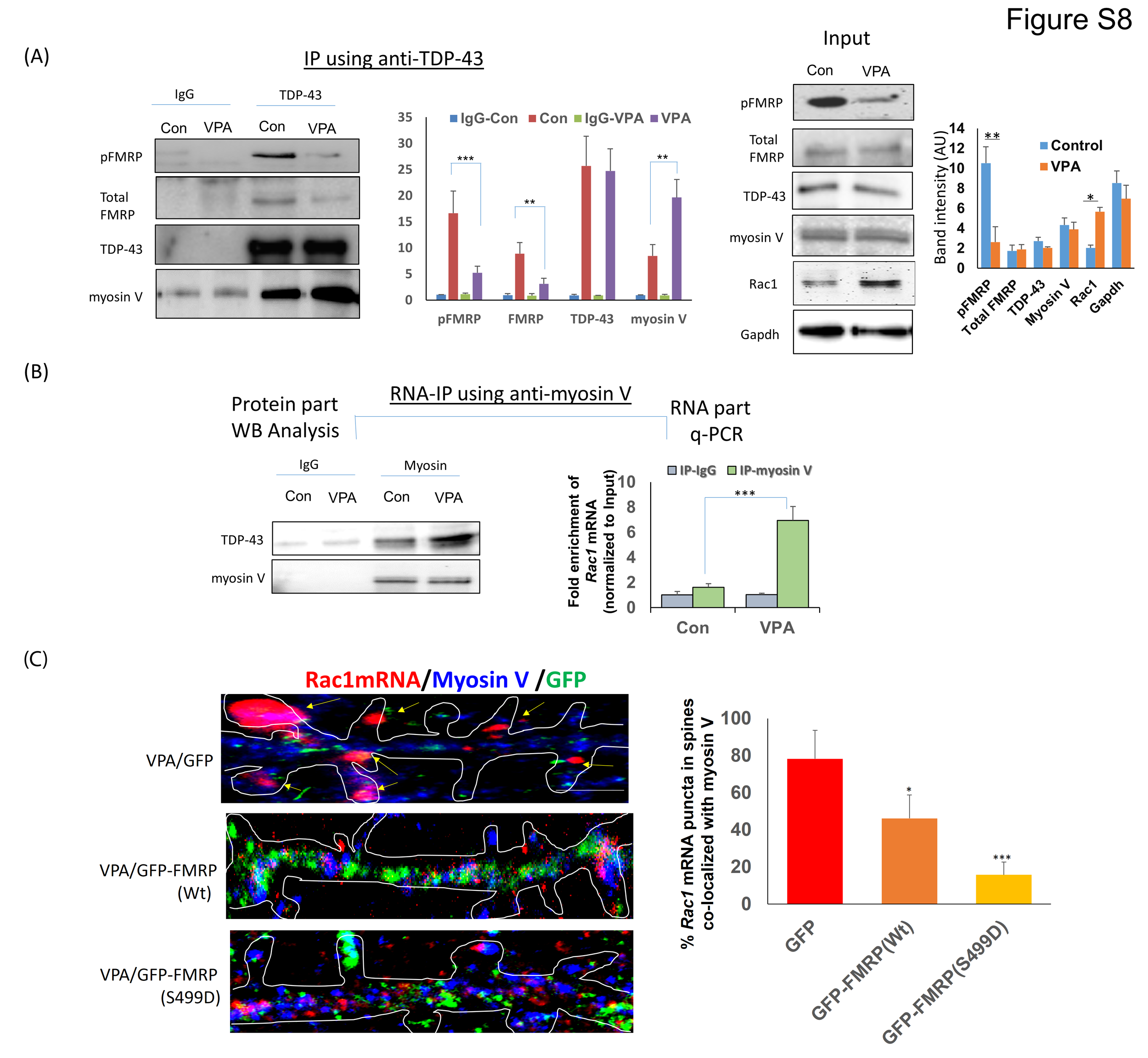

Supplement: Supplementary file 22 — Supplementary Material 22. [file 11658_2024_684_MOESM22_ESM.tif]

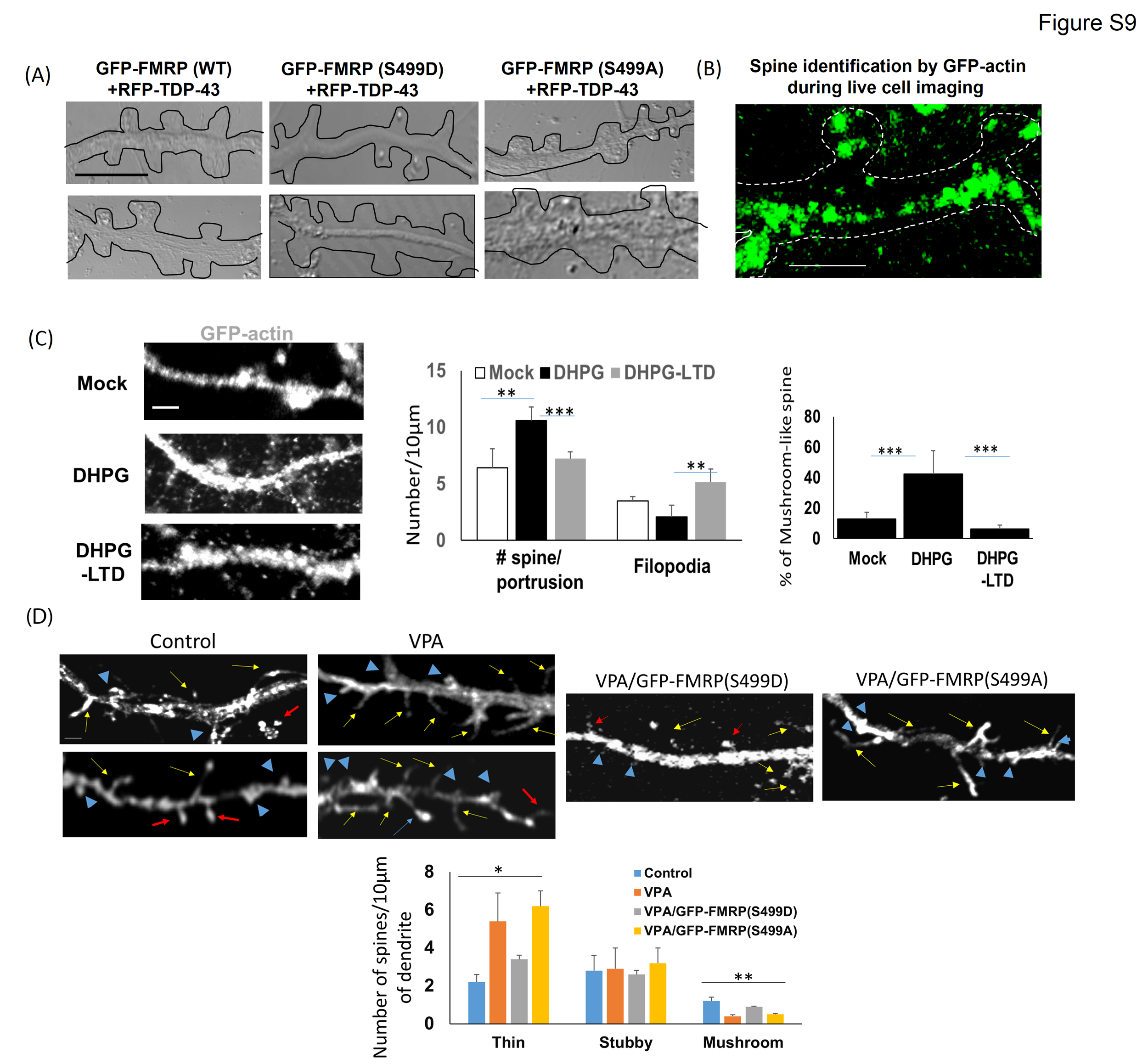

Supplement: Supplementary file 23 — Supplementary Material 23. [file 11658_2024_684_MOESM23_ESM.tif]
