# Supplementary material for: Molecular switch of the dendrite-to-spine transport of TDP-43/FMRP-bound neuronal mRNAs and its impairment in ASD
Source: Cell Mol Biol Lett. 2025 Jan 15;30:6. doi: 10.1186/s11658-024-00684-5 (PMC11737055; doi:10.1186/s11658-024-00684-5)
Supplement: Supplementary file 14 — Supplementary Material 14. [file 11658_2024_684_MOESM14_ESM.docx]

**Supplementary Figure Legends**

**Figure S1 Transport and translation dynamics of *Rac1* RNA granules in the spine region** **of DIV 14 primary hippocampal neurons under Mock and under DHPG treatment for a brief time period. (Related to Fig. 1A)**

(A) Magnified spine regions from the representative time-lapse images of Figure 1A. Initially, un-translating (yellow dot) granules either exhibited movement in the anterograde direction (yellow arrow) or remained stationary (yellow dual headed arrow). During 3 min or 2 min of the recording time, they continued to move further (yellow arrow) along the dendrites and became stationary without (yellow dual headed arrow) or with translation (red dual headed arrow). Note that only under DHPG treatment did the granules enter the spines to start translation. Scale bars, 2µm.

(B) Statistical analysis comparing the time taken for the yellow un-translating granules to become red translating granules in Mock and DHPG treated neurons. The bar diagrams show the average number of the translation events occurring every 10 s during the recording time period of 180 s (Mock) or 130 s (DHPG). The data was derived from 12–16 dendrites. The experiment was repeated for 3 times (N=3). (C) The representative confocal microscopy images showing co-localization of *Rac1* mRNA(red) and GFP-actin(white) in the dendrite and spine regions of DIV 14 primary hippocampal neurons co-transfected with pGFP-actin and different siRNA oligos and also subjected to RNA FISH by using *Rac1* mRNA-specific probes. Spines are indicated by arrows. Magnified picture of spine is also shown. Scale bars, 2 µm. (D) and (E) Indicated white/(white+red)/(white+red+green) granules are magnified from the snapshots from the supplementary video V3 (D) and V4 (E). (D) Two different snapshots are shown from video V3 at 1’15” to 3’00” (E) Five different snapshots from supplementary video V4 at 0.18", 0.36", 0.42", 2´12" and 2´15" are shown. Note the dissociation of three-colored granules (white+red+green) into green and (red+white) granules.

**Figure S2 TDP-43-dependent association of *Rac1* mRNA with myosin V. (Related to Fig. 2A)**

(A) Histograms showing relative quantification of the indicated immunoprecipitated proteins with respect to immunoprecipitated TDP43 as shown in Fig. 2A(a). Error bars represent SEM (N = 3). One-way ANOVA was used to compare different treatment conditions; *q < 0.05, **q < 0.001.

(B) RNA-IP analysis of synaptosome extracts isolated from DIV14 primary hippocampal neurons transfected with Sc, TDPsi, or FMRPsi oligo. (a) Synaptosome extracts from cultured hippocampal neurons were immunoprecipitated with anti-myosin V, and the immunoprecipitated mRNAs were analyzed by qRT-PCR using *Rac1* mRNA-specific primers. The data were normalized with the respective inputs and were represented as fold-enrichment relative to the Sc-IgG control (mean ± SD; N = 3). (b) The immunoprecipitates were also analyzed for associations of myosin V with TDP-43 by Western Blotting. Left, representative Western blotting patterns showing differential association between myosin V and TDP-43 under different knock down conditions. Right, bar diagram showing the average fold enrichment of TDP-43 protein derived from three independent protein IP experiments, using anti-myosin V compared to the IgG control. Error bars represent SEM. Student’s t test was carried out to compare the means and indicated as ***p < 0.0001. Inputs are the expression level of TDP-43, FMRP and myosin V under different conditions.

(C) Histograms showing the average ratios of immunoprecipitated TDP43 to immunoprecipitated myosin V under different knock down conditions from three independent IP experiments (N=3). Error bars represent SEM. One-way ANOVA was used to compare different knockdown conditions; *q < 0.05.

**Figure S3 Co-immunofluorescence (co-IF) staining analysis of the distribution patterns of TDP-43 and myosin V in dendritic sub-compartments of CX-4945 treated DIV12 primary hippocampal neurons. (Related to Fig. 2)**

The neurons were treated with DMSO (Mock) or with the CK2 inhibitor CX-4945 for the indicated time followed by IF-staining with anti-myosin V (red) or anti-TDP-43 (green). In the lower panels of the representative confocal microscopy images, the arrows indicate TDP-43 and myosin V co-localized puncta. Scale bar, 2 μm. The statistical bar diagram (bottom) shows the proportion (%) of TDP-43/ myosin V co-localized dendritic puncta located in the spines of the primary hippocampal neurons under different conditions (N=3; n=18 to 26 dendrites for each condition). Error bars represent SEM. Student’s t test was carried out to compare the means. *p < 0.05, ***p < 0.0001.

**Figure S4 DHPG-LTD-mediated ERK1/2 activation, FMRP phosphorylation, and increased co-localization of FMRP with TDP-43 in the spines of DIV 14 primary hippocampal neurons. (Related to Fig. 4)**

(A) The synaptosomal extracts harvested from DIV10 primary hippocampal neurons were subjected to treatment with DMSO (Mock) and DHPG for 30 s (DHPG) or 5 min (DHPG-LTD) followed by western blotting using indicated antibodies.

(B) IF staining was carried out using anti-pFMRP. Representative confocal microscopy images are shown (top). Statistical analysis comparing the relative fluorescence intensities representing the relative amounts of pFMRP is shown in the bar diagram below (N=3).

(C) Co-IF analysis was conducted using anti-TDP-43 and anti-FMRP. Representative confocal microscopy images are shown with the arrows indicating TDP-43/FMRP co-localized puncta inside the spines (top). Scale bars, 5 µm. Statistical analysis of the % of TDP-43 granules co-localized with FMRP in spines is shown in the bar diagram (N=3). Error bars represent SEM. Student’s t test was carried out to compare the means. **p < 0.001.

**Figure S5 Identification and characterization of TDP-43/*Rac1* mRNP granules and their association with other proteins under brief DHPG treatment and DHPG-LTD. (Related to Fig. 4)**

Co-IF staining analysis was carried out using (A) anti-TDP-43 and anti-myosin V; (B) anti-TDP-43 and anti-kinesin 1. Representative confocal microscopy images of the co-localization of TDP-43 with myosin V or kinesin 1 are shown with the arrows indicating the co-localized puncta.

The hippocampal neurons were also subjected to RNA FISH by using probes specific for *Rac1* mRNA and co-IF staining using anti-myosin V (C) or anti- kinesin1 (D). Representative confocal microscopy images of *Rac1* mRNA co-localized with myosin V or kinesin 1 are shown with arrows indicating the co-localized puncta. For (A), (B), (C), and (D), the corresponding DIC images are shown either below or beside the fluorescence image panels and used to predict the boundary of the dendrites and spines in the representative images. Scale bars, 2µm. Magnified images of specific spine regions and the statistical analysis of co-localization (%) are presented in Fig. 4A.

(E) The DIV14 primary hippocampal neurons treated with brief DHPG treatment for 30 s (DHPG) or for long DHPG treatment for 5 min (DHPG-LTD) were subjected to IF/FISH. Representative high-resolution 3D SIM images of co-staining using anti-TDP-43 (green) antibody and Rac1 mRNA probe (grey) together with anti-myosin V antibody (red, left) or anti-FMRP antibody (blue, right) are shown. Distance between TDP-43 and myosin V (n=12 mRNPs), and TDP-43 and FMRP(n=11 mRNPs) are measured under brief DHPG treatment and DHPG-LTD, respectively and shown.

**Figure S6 Microtubule invasion in the spines followed by FMRP dependent and Ca^2+^-mediated spine localization of TDP-43/kinesin 1/cortactin granules. (Related to Fig. 4)**

(A) The primary hippocampal neurons at DIV10 were treated without or with DHPG for 30s, 1 min, 2 min, 3 min and 5 min followed by immunofluorescence staining with α-tubulin and F-actin antibodies. The representative images without or with DHPG-treatment at above time points are shown. The yellow arrows indicate association of α-tubulin with F-actin inside the dendritic spines.

(B) The primary hippocampal neurons at DIV10 were treated without (Mock) or with DHPG for 30 s (DHPG) and 5 min (DHPG-LTD) followed by co-staining with anti-α-tubulin, anti-F-actin and anti-FMRP antibodies. The representative images of the Mock, DHPG and DHPG-LTD treated neurons are shown in the top panels. The bottom panel shows the histograms representing the percentage of spines with microtubule insertion under Mock, DHPG and DHPG-LTD conditions. The standard deviations under each condition were calculated from 15-17 dendrites. *** indicates p<0.0001.

(C) Histograms showing the quantification of the indicated immunoprecipitated proteins with respect to immunoprecipitated cortactin as shown in Fig. 4C(a). Error bars represent SEM (N = 3). One-way ANOVA was used to compare different treatment conditions; q** < 0.001.

(D) IP analysis of synaptosome extracts isolated from DIV10-12 primary hippocampal neurons under conditions of transfection of Sc oligo followed by mock treatment (mock+sc), transfection of Sc oligo followed by DHPG-LTD (DHPG-LTD), and FMRP siRNA oligo transfection followed by DHPG-LTD (DHPG-LTD+FMRPsi) . Synaptosome extracts are immunoprecipitated with anti-cortactin, and the pulled-down proteins are analyzed by Western blotting (N = 2 to 5) to identify the association of cortactin with TDP-43 and kinesin 1. Bar diagram showing the average fold enrichment of different proteins using anti-myosin cortactin compared to the IgG control. Error bars represent SEM. Student’s t test was carried out to compare the means and indicated as *p < 0.05. Input panels show the levels of different proteins in the synaptosome extracts used in the IP assay. Bar diagram represent average band intensity relative to kinesin 1. Error bars represent SEM.

**Figure S7 Characterization of un-translating and translating *Rac1* mRNP granules by 3D SIM microscopy. (Related to Fig. 5)**

(A) High-resolution images and distance analysis of TDP-43 /*Rac1* mRNA in un-translating (UT, un-bound with puromycin) and translating (TL, bound with puromycin) granules in the spines of DIV14 hippocampal neurons. Representative 3D SIM images from co-IF staining with use of *Rac1* mRNA probes (blue), anti-TDP-43 (red), and anti-puromycin (green) under Mock and short DHPG conditions are shown in (a). Bar diagram in (b) represents the statistical analysis of the distance variations between *Rac1* mRNA and associated TDP-43 in the UT and TL mRNP granules, respectively. Data was derived from 22-25 mRNP granules (n=22-25) in the dendritic spines from two biological repeats (N=2). Error bars represent SEM.

(B) High-resolution images and comparative analysis of the distance between myosin V and *Rac1* mRNA in UT and TL granules in the spines of DIV14 hippocampal neurons. Representative 3D SIM images are shown in (a) and statistical analysis of the distance variations between *Rac1* mRNA and associated myosin V in the UT and TL mRNP granules, respectively, is shown in (b). A total of 7-13 mRNP granules in the spines was analyzed for each data set (n=7-13). The experiment was repeated twice (biological repeats, N=2). Error bars represent SEM.

(C) High-resolution images and comparative analysis of the distance between TDP-43 and *Rac1* mRNA in granules without (-Myo) and with (+Myo) myosin V in the spines of DIV14 hippocampal neurons. The representative images are shown in (a). *Rac1* mRNA probes (blue), anti-TDP-43 (green) and anti-myosin V (red). Scale bars, 200 nm. (b) Bar diagrams representing statistical analysis of the distance variations between *Rac1* mRNA and associated TDP-43 in (-Myo) and (+Myo) mRNP granules. Data was derived from 20-25 mRNP granules (n=20-25) in the dendritic spines from two biological repeats (N=2). Error bars represent SEM. Student’s t test was carried out to compare the means and indicated as *p < 0.05, ***p < 0.0001.

**Figure S8 Reduced phosphorylation of FMRP and Impairment in TDP-43/FMRP guided spine transport mechanism in VPA-ASD mouse model(Related to Fig. 6)**

(A) IP analysis of synaptosome extracts isolated from DIV14 primary hippocampal neurons derived from control and VPA-ASD mouse models. Synaptosome extracts were immunoprecipitated either with non-specific IgG or anti TDP-43 and analyzed by Western blotting (N = 3) to identify the associations of pFMRP, total FMRP, and myosin V proteins, respectively, with TDP-43. Representative western blot gel picture is shown at left. The statistical analysis of fold enrichment of different proteins are shown as bar diagram at right; Input: Synaptosome extracts were directly analyzed by Western blotting to identify the levels of the indicated proteins without any further treatment. Bar diagram represent band intensity in AU. Error bars represent SEM. Student’s *t*-test was carried out to compare the means. **p* < 0.05, ***p* < 0.001, ****p* < 0.0001. (B) RNA-IP analysis of synaptosome extracts isolated from DIV14 primary hippocampal neurons derived from control and VPA-ASD mouse models. Synaptosome extracts from cultured hippocampal neurons were immunoprecipitated either with non-specific IgG or anti-myosin V, and the immunoprecipitated mRNAs were analyzed by qRT-PCR using *Rac1* mRNA-specific primers (right panel). The data were normalized with the respective inputs and were represented as fold-enrichment relative to the Sc-IgG control (mean ± SD; N = 3). The immunoprecipitates were also analyzed for associations of myosin V with TDP-43 by Western Blotting (left panels).

(C) Primary hippocampal neurons derived from the control mice and VPA-ASD mouse model were transiently-transfected with constructs expressing GFP, GFP-FMRP, or GFP-FMRP(S499D) followed by RNA FISH analysis using probes specific for *Rac1* mRNA, IF staining using anti-myosin V, and monitoring of the GFP fluorescence. Representative confocal microscopy images (left panels) showing the co-localization (the arrows) of *Rac1* mRNA with myosin V in the spine regions. Scale bars, 5 µm. Boundaries of the dendrites and spine were determined from the corresponding DIC images (data not shown). Statistical analyses of the co-localization (%) between *Rac1* mRNA and myosin V in spines from 6 to 9 dendritic regions (n=6-9) from 2-3 sets of independent experiments (N = 2-3) are represented by the bar diagrams (right panel). Error bars represent SEM. Student’s *t*-test was carried out to compare the means. **p* < 0.05, ***p* < 0.001.

**Figure S9** Identification and characterization of different kinds of dendritic spines (**Related to Fig. 3B(A), Supplementary video V6 and Fig. 5A)**

(A) Detection of dendrite and spine boundary from DIC images of DIV 14 primary hippocampal neurons co-expressing RFP-TDP-43 and GFP-tagged Wt or mutant FMRP proteins. The spine localization and boundary of the dendritic regions are indicated by black lines. Scale bars, 5µm

(B) Detection of dendrite and spine boundaries from the distribution of GFP-actin in DIV 14 primary hippocampal neurons co-transfected with pGFP-actin and pRFP-TDP-43 followed by incubation with molecular beacon against Rac1 mRNA. Snapshot picture of the video was shown when only green channel is open. The spine localization and boundary of the dendritic regions are determined by DIC image and GFP-actin accumulation inside the spine and indicated by white lines. Scale bars, 3µm.

(C) Increase of the density of mushroom-like spines in cultured hippocampal neurons upon brief DHPG treatment, but not under long DHPG treatment. Primary hippocampal neurons at DIV 14 were transfected with pGFP-actin followed by different treatments. The total number of spines/protrusions and filopodia as well as the proportions of mushroom-like spines, as defined by the GFP-actin patterns, were determined. Representative confocal microscopy images are shown. Scale bars, 2µm. Bar diagrams represent statistical analysis of the average number of spines and filopodia, respectively, in 10µm of dendritic regions and the % of mushroom–like spines present under the indicated treatment conditions. Data were derived from 20-30 dendritic regions analyzed in three biological repeats (N=3). Error bars represent SEM. Student’s t test was carried out to compare the means and indicated as **p < 0.001, ***p < 0.0001.

(D) Increase of the density of thin spines in cultured hippocampal neurons from VPA-ASD mouse model compared to Control. This phenomenon can be overturned upon over expression of phosphomimetic mutant FMRP(S499D), whereas dephosphomimetic mutant FMRP(S499A) shows no significant effect. Primary hippocampal neurons at DIV 12 were transfected with pGFP-actin The number of different kinds of spines as defined by the GFP-actin patterns: thin spine (yellow arrow), stubby spine (blue arrow head), and mushroom-like spine (red arrow) were determined. Representative confocal microscopy images are shown. Scale bars, 2µm. Bar diagrams represent statistical analysis of the average number of different spines in 10µm of dendritic regions from 8 to 10 different neurons (N=2). Error bars represent SEM. One-way ANOVA was carried out to compare the means and indicated as *q < 0.05, **q < 0.001.
