# Supplementary material for: Molecular switch of the dendrite-to-spine transport of TDP-43/FMRP-bound neuronal mRNAs and its impairment in ASD
Source: Cell Mol Biol Lett. 2025 Jan 15;30:6. doi: 10.1186/s11658-024-00684-5 (PMC11737055; doi:10.1186/s11658-024-00684-5)
Supplement: Supplementary file 24 — Supplementary Material 24. [file 11658_2024_684_MOESM24_ESM.docx]

**Table 1. Transport dynamics of TRICK-*Rac1* 3’UTR reporter RNA granules in DIV14 mouse primary hippocampal neurons under Mock and under DHPG treatment for brief period of time**

|  | % of moving granules | % of moving granules with anterograde displacement | % of moving granules with retrograde displacement | Net displacement in the anterograde direction (µm) | Net displacement in the retrograde direction (µm) | Avg. anterograde velocity (µm/s) | Avg. retrograde velocity (µm/s) |
| --- | --- | --- | --- | --- | --- | --- | --- |
| Mock | 29.6 | 51.4 | 48.6 | 8.8 | -5.8 | 0.33 | -0.24 |
| DHPG | 26.6 | 47.5 | 52.5 | 4.3* | -5.1 | 0.12** | -0.21 |

DIV14 primary hippocampal neurons expressing TRICK-*Rac1* 3’UTR reporter RNA were subjected to Mock or DHPG treatment for ~ 1 min. The transport dynamics of the reporter RNA granules from the dendrites under Mock (41 granules analyzed from 18 to 22 dendrites) or DHPG treatment condition (29 granules analyzed from 18 to 24 dendrites) were then analyzed by live-cell imaging with manual tracking using Imaris software. Significant differences were observed in the net displacement in the anterograde direction (*p<0.01) and for the average anterograde velocity (**p<0.001). The experiment was repeated three times (N=3).
