# Supplementary material for: Molecular switch of the dendrite-to-spine transport of TDP-43/FMRP-bound neuronal mRNAs and its impairment in ASD
Source: Cell Mol Biol Lett. 2025 Jan 15;30:6. doi: 10.1186/s11658-024-00684-5 (PMC11737055; doi:10.1186/s11658-024-00684-5)
Supplement: Supplementary file 25 — Supplementary Material 25. [file 11658_2024_684_MOESM25_ESM.docx]

|  | **% of granules pausing near the spine base** | **% of the pausing granules that enter into the spines** | **% of granules pausing and entering into the spines that get translated** | **Average time of pausing before entering the spines(s)** |
| --- | --- | --- | --- | --- |
| Mock | 51 | 82 | 91 | 186 |
| DHPG | 84** | 85 | 95 | 130* |

**Table 2. Alterations of the transport and translation dynamics of TRICK-*Rac1* 3’UTR reporter RNA granules in the spine area of DIV14 mouse primary hippocampal neurons upon brief DHPG treatment.**

The transport and translation dynamics of the reporter RNA granules in the spine area of DIV 14 primary hippocampal neurons was analyzed by live-cell imaging technique. The data show the significant differences in the proportion of granules pausing near the spine bases (**p<0.001) and the average pausing time (*p<0.01) of granules between Mock-treated (number of granules, n=34 from 18 to 20 dendrites) and DHPG-treated (number of granules, n=22 from 20 to 22 dendrites) neurons. The experiment was repeated three times (N=3).
