# Supplementary material for: Molecular switch of the dendrite-to-spine transport of TDP-43/FMRP-bound neuronal mRNAs and its impairment in ASD
Source: Cell Mol Biol Lett. 2025 Jan 15;30:6. doi: 10.1186/s11658-024-00684-5 (PMC11737055; doi:10.1186/s11658-024-00684-5)
Supplement: Supplementary file 26 — Supplementary Material 26. [file 11658_2024_684_MOESM26_ESM.docx]

**Table 3**. **The composition and dynamics of dendrite-to-spine transport of *Rac1* mRNP granules in DIV 14 primary hippocampal neurons expressing exogenous GFP-FMRP and RFP-TDP-43 under different conditions.**

*

**

|  | **% of three-colored GFP-FMRP/RFP-TDP-43/*Rac1* mRNA granules getting dissociated from GFP-FMRP protein before entering the spines** |
| --- | --- |
| **Mock** | **28.2** |
| **DHPG** | **68.1** |
| **DHPG+OA** | **36.9** |

DIV 14 primary hippocampal neurons co-transfected with pGFP-FMRP and pRFP-TDP-43 were subjected to live-cell imaging of endogenous *Rac1* mRNA using a specific FISH probe (white), GFP-FMRP (green) and RFP-TDP-43 (red) under Mock (Mock, n=25 from 22 dendrites), DHPG treatment for 30 s (DHPG, n=28 granules from 17 dendrites) and DHPG and OA co-treatment for 30 s (DHPG+OA, n=17 from 18 dendrites) conditions described in ‘Materials and Methods’. The proportions (%) of three-colored GFP-FMRP/RFP-TDP-43/*Rac1* mRNA granules dissociated from GFP-FMRP protein before the spine entrance during the tracking time period are listed. The data show the significant differences among the Mock, short DHPG, and (DHPG+OA) treatment conditions. Student’s t test was carried out to compare the means. **p<0.001, *p<0.01. The experiment was repeated three times (N=3).
