# Supplementary material for: Molecular switch of the dendrite-to-spine transport of TDP-43/FMRP-bound neuronal mRNAs and its impairment in ASD
Source: Cell Mol Biol Lett. 2025 Jan 15;30:6. doi: 10.1186/s11658-024-00684-5 (PMC11737055; doi:10.1186/s11658-024-00684-5)
Supplement: Supplementary file 27 — Supplementary Material 27. [file 11658_2024_684_MOESM27_ESM.docx]

|  | **% of granules pausing near the spine base**  **(Rests are passerby)** | **% of the pausing granules that enter into the spines** | **Average time of pausing before entering the spines(s)** |
| --- | --- | --- | --- |
| Mock | 37 | 89 | 99 |
| CX4945-6 h | 28 | 86 | 87 |
| CX4945-12 h | 51 | 88 | 68 |
| CX4945-24 h | 71^*^ | 95 | 47^*^ |

**Table 4. Changes of the dynamics of the dendrite-to-spine transport of exogenous RFP-tagged TDP-43 / endogenous *Rac1* mRNA co-localized granules in DIV14 mouse primary hippocampal neurons treated with CX4945.**

DIV14 primary hippocampal neurons transfected with pRFP-TDP-43 followed by treatment with CX4945 for 6 h, 12 h, or 24 h were subjected to live-cell imaging experiment, following the parameters listed in ‘Materials and Methods’ section, to study the transport dynamics of the RFP-TDP-43/*Rac1* mRNA co-localized granules near the spine area. Significant differences were detected in the proportion of granules pausing near the spine base (*p<0.01) and the average pausing time (*p<0.01) between the Mock (number of granules, n=28 from 20 to 23 dendrites) and CX4945-24 h (number of granules, n=25 from 16 to 20 dendrites) treatment conditions. The experiment was repeated three times (N=3).
