# Supplementary material for: Molecular switch of the dendrite-to-spine transport of TDP-43/FMRP-bound neuronal mRNAs and its impairment in ASD
Source: Cell Mol Biol Lett. 2025 Jan 15;30:6. doi: 10.1186/s11658-024-00684-5 (PMC11737055; doi:10.1186/s11658-024-00684-5)
Supplement: Supplementary file 28 — Supplementary Material 28. [file 11658_2024_684_MOESM28_ESM.docx]

**Supplementary Movie Legends**

**Supplementary Video V1_mock.** Real-time imaging of TRICK-*Rac1* 3′UTR reporter RNA granules in a dendrite and spines of a DIV14 primary hippocampal neuron under Mock treatment condition. Related to Fig. 1A. This video shows un-translating (yellow) granules moving in the anterograde direction across the dendrites without entering into the spines. Most of these granules did not translate within 3 min of starting the recording. Dendritic and spine boundary were determined from DIC images.

**Supplementary Video V2_DHPG.** Real-time imaging of TRICK-*Rac1* 3′UTR reporter RNA granules in a dendrite and spines of a DIV14 primary hippocampal neuron under DHPG treatment for ~1 min. Related to Fig. 1A. This video shows the un-translating (yellow) granules moving slowly across the dendrite, accumulating near the spine base, with one granule entering the spine. Many of these granules were translating (yellow granule becoming red) inside the spine or at the spine base within 2 min of starting the recording. Dendritic and spine boundary were determined from DIC images.

**Supplementary Video V3 _mock.** Co-trafficking of GFP-FMRP (green), RFP-TDP-43 (red), and *Rac1* mRNA (white, probed with a molecular beacon against *Rac1* 3′UTR and tagged with Cy5) in pGFP-FMRP- and pRFP-TDP-43-transfected DIV14 primary hippocampal neurons under the Mock. Related to Fig. 1C and Table 3. This video shows three-colored (white+green+red) granules moving across the dendrite in the anterograde direction without entering into the spines. Dendritic and spine boundary were determined from DIC images.

**Supplementary Video V4_DHPG.** Movement and dissociation of GFP-FMRP (green), RFP-TDP-43 (red), and *Rac1* mRNA (white, probed with a molecular beacon against *Rac1* 3′UTR and tagged with Cy5) colocalized granules in pGFP-FMRP- and pRFP-TDP-43-transfected DIV14 primary hippocampal neurons under DHPG treatment for 1 min. Related to Fig. 1C and Table 3. One of the three-colored granules is dissociated at the spine base. The video shows the dissociated (white+red) granule entering into the spine, while the green granule (FMRP) continues moving along the dendrite in the anterograde direction. Meanwhile, a yellow granule moves along the dendrite in the anterograde direction without any GFP-FMRP dissociation. Dendritic and spine boundary were determined from DIC images.

**Supplementary Video V5_DHPG+OA.** Co-trafficking of GFP-FMRP (green), RFP-TDP-43 (red), and *Rac1* mRNA (white, probed with a molecular beacon against *Rac1* 3′UTR and tagged with Cy5) in pGFP-FMRP- and pRFP-TDP-43-transfected DIV14 primary hippocampal neurons under co-treatment with DHPG and OA for 1 min. Related to Table 3. Similar to the Mock treatment condition, this video shows the granules moving across the dendrite predominantly in the anterograde direction without entering into the spines. Dendritic and spine boundary were determined from DIC images.

**Supplementary Video V6.** Time-lapse imaging of RFP-TDP-43 and *Rac1* mRNA (white, probed with a molecular beacon against *Rac1* 3′UTR and tagged with Cy5) granules in pRFP-TDP-43- and pGFP-actin co-transfected DIV14 primary hippocampal neurons under the Mock treatment condition. Only red (RFP-TDP-43) and white (*Rac1* mRNA) granule movement were shown in the movie. GFP-actin localization was used to determine the boundary of dendrites and spine regions, exemplified in Fig. S7B. Related to Supplementary Fig. S3 and Table 4. Colocalized (white+red) granules show anterograde and retrograde movement in the dendrites without entering into spines.

**Supplementary Video V7.** Time-lapse imaging of RFP-TDP-43 and *Rac1* mRNA (white, probed with a molecular beacon against *Rac1* 3′UTR and tagged with Cy5) granules in pRFP-TDP-43 and pGFP-actin co-transfected DIV14 primary hippocampal neurons subjected to CX-4945 treatment for 12 h. Only red (RFP-TDP-43) and white (*Rac1* mRNA) granule movement were shown in the movie. GFP-actin localization was used to determine the boundary of dendrites and spine regions, exemplified in Fig. S7B. Related to Supplementary Fig. S3 and Table 4. In contrast to the Mock treatment condition, colocalized (white+red) granules enter into the spine or accumulate near the spine base.
